# Peptide Conjugates of a 2′-*O*-Methoxyethyl Phosphorothioate Splice-Switching Oligonucleotide Show Increased Entrapment in Endosomes

**DOI:** 10.1021/acsomega.3c05144

**Published:** 2023-10-17

**Authors:** Alyssa C. Hill, J. Philipp Becker, Daria Slominski, François Halloy, Christoffer Søndergaard, Jacob Ravn, Jonathan Hall

**Affiliations:** † Institute of Pharmaceutical Sciences, Department of Chemistry and Applied Biosciences, Eidgenössische Technische Hochschule Zürich (ETH Zürich), Zürich 8093, Switzerland; ‡ Roche Innovation Center Copenhagen (RICC), Hørsholm 2970, Denmark

## Abstract

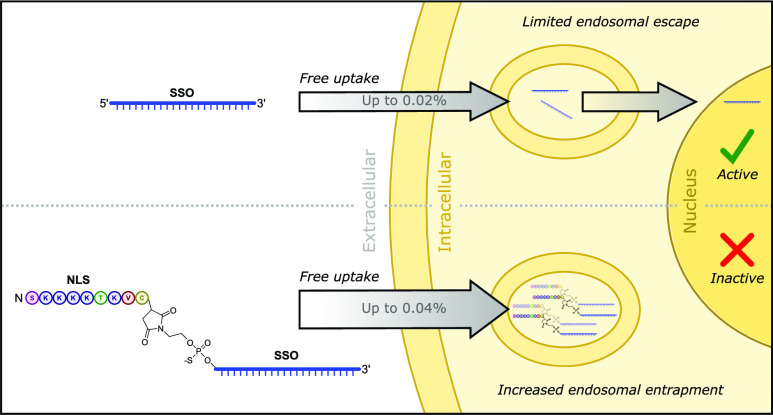

Antisense oligonucleotides (ASOs) are short, single-stranded nucleic acid molecules that alter gene expression. However, their transport into appropriate cellular compartments is a limiting factor in their potency. Here, we synthesized splice-switching oligonucleotides (SSOs) previously developed to treat the rare disease erythropoietic protoporphyria. Using chemical ligation-quantitative polymerase chain reaction (CL-qPCR), we quantified the SSOs in cells and subcellular compartments following free uptake. To drive nuclear localization, we covalently conjugated nuclear localization signal (NLS) peptides to a lead 2′-*O*-methoxyethyl phosphorothioate SSO using thiol–maleimide chemistry. The conjugates and parent SSO displayed similar RNA target-binding affinities. CL-qPCR quantification of the conjugates in cells and subcellular compartments following free uptake revealed one conjugate with better nuclear accumulation relative to the parent SSO. However, compared to the parent SSO, which altered the splicing of the target pre-mRNA, the conjugates were inactive at splice correction under free uptake conditions *in vitro*. Splice-switching activity could be conferred on the conjugates by delivering them into cells via cationic lipid-mediated transfection or by treating the cells into which the conjugates had been freely taken up with chloroquine, an endosome-disrupting agent. Our results identify the major barrier to the activity of the peptide–oligonucleotide conjugates as endosomal entrapment.

## Introduction

Antisense oligonucleotides (ASOs) are short (18–25 nucleotide; nt), single-stranded nucleic acid molecules that alter gene expression via hybridization to a target RNA.^1^ The two main classes of ASOs either promote target RNA degradation by recruiting RNase H (i.e., gapmers) or modulate target RNA splicing or translation without triggering degradation (e.g., splice-switching oligonucleotides; SSOs).^2^ Such RNA-targeted therapies are a platform for drug discovery. However, in unmodified form, nucleic acids have poor drug-like properties and pose a two-part delivery problem.^3^ To arrive at the tissue of interest, an ASO must survive nuclease digestion in the blood, traverse the vascular endothelial barrier, evade the reticuloendothelial system, and avoid renal clearance.^4^ To arrive at the right intracellular compartment, the ASO must gain entry into a cell, navigate a complex and dynamic intracellular trafficking network, and breach the endosomal barrier to access the cytosol or nucleus.^5^

The first challenge has largely been met with the advent of structural modifications that improve the drug-like properties of oligonucleotides,^6^ especially the phosphorothioate (PS) backbone modification.^7^ PS ASOs benefit from broad tissue distribution, extended circulation times, and reduced urinary excretion.^8^ Interestingly, the PS modification also promotes the free uptake of ASOs into cells in a process termed gymnosis.^9,10^ Gymnotic delivery is attributed to the interaction of PS ASOs with heparin-binding proteins on the cell surface^11^ but remains an incompletely understood phenomenon, with dependence on oligonucleotide sequence,^9^ length,^9,12^ chemistry,^12,13^ and concentration^9,12,14^ as well as cell type^9,15^ and physiological state.^16^ Second-generation (e.g., 2′-*O*-methoxyethyl, or MOE,^17^ and locked nucleic acid, or LNA^18,19^) modifications further increase the nuclease stability of PS ASOs and offset losses in RNA target-binding affinity caused by the PS groups (Δ*T*_m_ of −0.5 °C per substitution).^20^ Presently, ASOs of the MOE chemical class have been evaluated in thousands of humans,^21^ and five MOE PS ASOs are approved drugs.^22^

Relative to small molecules, which passively diffuse across the cell membrane’s lipid bilayer,^23^ ASOs are large, hydrophilic, and polyanionic molecules, and they do not readily cross the cellular membrane.^24^ Today, the cellular uptake of ASOs is achieved via gymnosis^9^ or by conjugation to a triantennary *N*-acetylgalactosamine (GalNAc) ligand.^25^ Strategies to achieve uptake into extrahepatic cell types, including conjugating lipids,^26^ peptides,^27,28^ and antibodies^29^ to ASOs, are emerging.^30^ However, following endocytosis, ASOs in endocytic vesicles either recycle back to the extracellular space or progress to lysosomes, where they are degraded.^12^ Moreover, although RNase H-recruiting gapmers display some activity in the cytoplasm,^31^ both RNase H-mediated cleavage and steric block modes of action primarily occur in the nucleus, where ASO accumulation is limited.^32^ Buntz et al. have shown that approximately 100,000 gapmer molecules are required to suppress gene expression by >50%,^33^ and Pendergraff et al. have shown that 247,000 to 693,000 gapmers per nucleus result in similar target reduction for different sequences.^34^ We reasoned that there is significant potential to increase ASO potency by driving nuclear localization.

In this study, we aimed to identify nuclear localization signal (NLS) peptides that, when conjugated to an oligonucleotide, improve its nuclear localization and biological activity. As model ASOs, we selected SSOs previously developed to treat erythropoietic protoporphyria (EPP).^35,36^ EPP is a rare disease caused by a deficiency in the enzyme ferrochelatase (FECH), which catalyzes the incorporation of ferrous iron into protoporphyrin IX (PPIX) to form heme.^37^ In 90% of patients with EPP, the disease results from an alteration of both FECH alleles: on one, a deleterious mutation, and on the other, a single nucleotide polymorphism (SNP; c.315–48T > C).^38^ The c.315-48C allele bolsters the use of a cryptic splice site between exons 3 and 4 and increases the production of an aberrant FECH transcript; this transcript carries a premature stop codon and is degraded.^35^ In EPP patients, PPIX accumulates in circulating red blood cells.^39^ On exposure to visible light, PPIX produces reactive oxygen species, and patients experience acute phototoxic reactions. Our selected SSOs target the c.315-48C polymorphism and 3′ cryptic acceptor splice site in FECH pre-mRNA and increase the level of correctly spliced FECH transcript.^35,36^

Here, we employed chemical ligation-quantitative polymerase chain reaction (CL-qPCR), a technique suitable for the detection of chemically modified oligonucleotides and oligonucleotide conjugates,^36,40,41^ to quantify the SSOs in cells and subcellular compartments following free uptake. Then, we covalently conjugated different NLS peptides^42,43^ to a lead MOE PS SSO using thiol–maleimide chemistry.^44^ Using CL-qPCR, we quantified the conjugates’ cellular uptake and subcellular localization following free uptake and then evaluated the conjugates’ splice-switching activities *in vitro*. Compared to the parent, unconjugated SSO, all conjugates were inactive at splice correction under free uptake conditions. However, the conjugates’ splice-switching activities could be rescued by transfecting them into cells or by treating the cells into which the conjugates had been freely taken up with chloroquine, an endosome-disrupting agent.^45^ Our results identify the major barrier to the activity of the peptide–oligonucleotide conjugates as endosomal entrapment—a long-standing, rate-limiting, and recalcitrant problem in the field^46^—and may help explain the results of previous studies on peptide–oligonucleotide conjugates that show modest or no improvement in potency over unconjugated oligonucleotides.^28,36^

## Materials and Methods

### Oligonucleotide Synthesis

Oligonucleotide synthesis was performed on a MerMade 12 Oligonucleotide Synthesizer (BioAutomation) using UnyLinker controlled-pore glass (CPG) with a pore size of 500 Å (ChemGenes N-4000-05) unless indicated otherwise. Bz-A-LA-CE Phosphoramidite (Glen Research 10-2000), dmf-G-LA-CE Phosphoramidite (Glen Research 10-2029), T-LA-CE Phosphoramidite (Glen Research 10-2030), 2′-MOE-A Phosphoramidite (Thermo Scientific 27-1019), 5-Me-MOE-C Phosphoramidite (Thermo Scientific 27-1020), 2′-MOE-G Phosphoramidite (Thermo Scientific 27-1022), and 2′-MOE-T Phosphoramidite (Thermo Scientific 27-1021) were prepared at 0.1 M in anhydrous acetonitrile (Acros Organics). Bz-5-Me-C-LA-CE Phosphoramidite (Glen Research 10-2011) was prepared in anhydrous acetonitrile/dichloromethane 1:1 (v/v). Activation of the phosphoramidites for coupling was achieved using 0.24 M 5-benzylthio-1*H*-tetrazole (Carbosynth FB02611) in acetonitrile. Cleavage of the dimethoxytrityl (DMT) group was achieved using 3% dichloroacetic acid (Sigma-Aldrich D54702) in dichloromethane. Oxidation was carried out using 0.02 M iodine in tetrahydrofuran (THF)/pyridine/water 7:2:1 (v/v) (Biosolve 15032402). Sulfurization was carried out using 0.1 M 3-[(dimethylaminomethylene)amino]-3*H*-1,2,4-dithiazole-5-thione (DDTT) in pyridine. Capping of failed sequences was performed either before oxidation or after sulfurization using 10% acetic acid anhydride, 10% lutidine in THF (Biosolve 03272402), and 16% *N*-methylimidazole in THF (Biosolve 03282402) unless indicated otherwise. Oligonucleotides were cleaved from the solid support in ammonium hydroxide solution (Sigma-Aldrich 221228) at 55 °C for 16 h and evaporated to dryness at 45 °C following the addition of 5 μL of 0.5 M Tris base. The DMT-on crude product was resuspended in ultrapure water for purification by reversed-phase high-performance liquid chromatography (RP-HPLC; Agilent 1200 Series) on an XBridge OST C18, 2.5 μm, 10 mm × 50 mm column (Waters 186003954) at 50 °C. The flow rate was 5 mL/min, and the gradient was 10–50% methanol in 0.1 M aq. triethylammonium acetate (TEAA) buffer, pH 8.0, in 5 min. The DMT group was removed using 20% aq. acetic acid at ambient temperature for 15 min. Oligonucleotides were evaporated to dryness and purified by RP-HPLC using a flow rate of 5 mL/min and a gradient of 5–20% methanol in 0.1 M aq. TEAA buffer, pH 8.0, in 5 min. Collected fractions were pooled, evaporated to dryness, and resuspended in ultrapure water.

### Synthesis of CL-qPCR Ligators

The biphenylsulfonyl (BPS) and phosphorothioate (PS) ligators used for CL-qPCR were synthesized on a MerMade 12 Oligonucleotide Synthesizer (BioAutomation) as previously described.^36,40^ Briefly, the synthesis of the PS ligator was performed on a 3′-Phosphate-ON solid support (ChemGenes N9977-05) by sulfurization of the coupling between the support and first nucleotide, following standard DNA synthesis. The BPS ligator was synthesized under “UltraMild” conditions using dT-Q-CPG support (Glen Research 21-2030). The dA and dG amidites were exchanged for Pac-dA-CE (Glen Research 10-1601) and iPr-Pac-dG-CE (Glen Research 10-1621), respectively. Capping of failed sequences was performed using 5% (w/v) phenoxyacetic anhydride (TCI Chemicals P0111) in THF/pyridine 90:10 (v/v) in place of acetic anhydride. Cleavage and deprotection were performed using 30% ammonium hydroxide at ambient temperature for 4 h. The PS ligator bears a 3′ terminal PS group that enables dimerization. To prevent spontaneous sulfur–oxygen exchange, the PS ligator was stored as a dimer. Immediately prior to use, an aliquot of the PS ligator was reduced by adding tris(carboxyethyl)phosphine hydrochloride (TCEP HCl; Fluorochem M02624) or Bond-Breaker TCEP Solution, Neutral pH (Thermo Scientific 77720) to a final concentration of 50 μM.

### Synthesis of a 5′-Capped-Maleimide-Modified Oligonucleotide

A 5′-capped-maleimide-modified oligonucleotide was synthesized using 5′-maleimide-modifier phosphoramidite (Glen Research 10–1938) as previously described.^47^ The oligonucleotide was cleaved from the solid support in ammonium hydroxide solution (Sigma-Aldrich 221228) at 35 °C for 18 h. The CPG was pelleted, and the supernatant was collected. The CPG was washed with 50% aq. ethanol. The supernatants were pooled, vacuum-concentrated at 35 °C, and diluted in ultrapure water for purification by RP-HPLC (Agilent 1200 Series) on an XBridge OST C18, 2.5 μm, 10 mm × 50 mm column (Waters 186003954) at 50 °C. The flow rate was 5 mL/min, and the gradient was 20–60% methanol in 0.1 M aq. TEAA buffer, pH 8.0, in 5 min. Collected fractions were pooled, vacuum-concentrated at 35 °C, and diluted in ultrapure water.

### Determination of Oligonucleotide Yield and Purity

To calculate the yield, the absorbance at 260 nm of each oligonucleotide was measured on a NanoDrop 2000 Spectrophotometer (Thermo Fisher Scientific). For oligonucleotides with DNA/LNA ribose chemistry, molar extinction coefficients at 260 nm were determined using the online OligoAnalyzer Tool available at https://eu.idtdna.com/calc/analyzer. The absorbances and molar extinction coefficients were used to calculate the concentrations according to the Beer–Lambert law. For oligonucleotides with MOE ribose chemistry, the absorbance was input into OligoCalc, an online tool available at http://biotools.nubic.northwestern.edu/OligoCalc.html, which outputs the concentration. Oligonucleotide purity was assessed by liquid chromatography–mass spectrometry (LC–MS) on an XBridge OST C18 column (Waters) at 65 °C. Injections were 1 nmol of oligonucleotide prepared in ultrapure water. The flow rate was 0.3 mL/min, and the gradient was 5–80% methanol in 0.4 M hexafluoroisopropanol-15 mM aq. triethylamine buffer in 15 min. The masses found were compared against the masses calculated using the Oligowizard Nucleic Acid Calculator, an online tool available at http://oligowizard.com/.

### Oligonucleotide–Peptide Conjugation

Peptides were purchased from GenScript (Piscataway, New Jersey) with N-terminal acetylation and dissolved in ultrapure water according to the manufacturer’s instructions. Conjugation reactions were performed under microwave irradiation at 90 °C and 90 W for 90 min using 100 μM 5′-capped-maleimide-modified oligonucleotide and 600 μM peptide (i.e., 6 mol equiv) in 8 mM TEAA buffer, pH 6.8. Conjugates were purified by RP-HPLC as described above. Collected fractions were pooled, vacuum-concentrated, and diluted in ultrapure water. The absorbance at 260 nm of each conjugate was measured as described above, and concentrations were calculated as previously described.^47^ Purity was assessed by LC–MS as described above.

### UV Melting

UV melting was performed as previously described.^47^ Briefly, oligonucleotides and conjugates were prepared at 2 μM in 100 mM NaCl, 10 mM phosphate, and 0.1 mM Na_2_EDTA, pH 7.0, in the presence of 2 μM RNA target with the sequence 5′-r(AGAAAACAUUUCUCAGGCUGCU)-3′ (Microsynth AG, Balgach, Switzerland). Absorbance at 260 nm was measured over the temperature range of 20 to 90 °C on a Cary 300 spectrophotometer (Varian) using a heating rate of 0.5 °C/min and a data collection interval of 0.5 °C. Hold periods of 5 min at both 20 and 90 °C were used to ensure thermal equilibrium. Mean melting temperatures (*T*_m_s) were determined from first derivative analyses of nonlinear fit melting curves in Microsoft Excel.

### Cell Culture

All cells were maintained at 37 °C with 5% CO_2_ in a humidified incubator. HEL cells were maintained in Roswell Park Memorial Institute (RPMI) 1640 Medium (Gibco 61870010) and 10% Fetal Bovine Serum (FBS; Gibco 10270106). K562 cells were maintained in Iscove’s Modified Dulbecco’s Medium (IMDM; Sigma-Aldrich I3390) supplemented with GlutaMAX (Gibco 35050038) and 10% FBS (Gibco 10270106). 293 cells were maintained in Dulbecco’s modified Eagle’s medium (DMEM; Gibco 31966021) and 10% FBS (Gibco 10270106).

### Preparation of Cell Lysates for CL-qPCR

HEL cells were counted using a Countess Automated Cell Counter (Invitrogen) or Countess 3 Automated Cell Counter (Invitrogen) and seeded in 24-well plates (Techno Plastic Products 92024) at 115,000 live cells per mL and 500 μL per well in complete medium. After 3–4 h, treatments were performed by diluting oligonucleotides or conjugates in 50 μL of Opti-MEM (Gibco 31985047) and directly adding the diluted oligonucleotides or conjugates to the cells. After 24 h, the cells were transferred to clean tubes and pelleted by centrifugation. The cells were washed with ice-cold phosphate-buffered saline (PBS; Gibco 10010015), ice-cold 0.1 mg/mL heparin (Sigma-Aldrich H3149) in PBS, and then with ice-cold PBS. The cells were lysed in Clarity OTX Lysis-Loading Buffer v2.0 (Phenomenex AL0-8579). Cell lysates were centrifuged at 13,000 rpm and 4 °C for 10 min, and supernatants were transferred to clean tubes prior to storage at −80 °C. Lysates were thawed on ice and diluted 1:75 in ultrapure water prior to analysis by CL-qPCR.

### Preparation of Subcellular Fractions for CL-qPCR

Subcellular fractions were prepared using the Rapid, Efficient, And Practical (REAP) method^48^ with minor changes. Briefly, HEL cells were counted, seeded, and treated as described above. After 24 h, the cells were transferred to clean tubes and pelleted by centrifugation. The cells were washed with ice-cold PBS (Gibco 10010015), ice-cold 0.1 mg/mL heparin (Sigma-Aldrich H3149) in PBS, and then with ice-cold PBS. Cellular membranes were lysed with ice-cold 0.1% (v/v) Nonidet P 40 (Fluka 74385) in PBS. Samples were centrifuged at 1000*g* and 4 °C for 1 min, and supernatants were transferred to clean tubes prior to storage at −80 °C (cytoplasmic fractions). Nuclear pellets were washed with ice-cold 0.1% (v/v) Nonidet P 40, followed by ice-cold PBS. The nuclei were lysed in Clarity OTX Lysis-Loading Buffer v2.0 (Phenomenex AL0-8579). Nuclear lysates were centrifuged at 13,000 rpm and 4 °C for 10 min, and supernatants were transferred to clean tubes prior to storage at −80 °C (nuclear fractions). All fractions were thawed on ice. Cytoplasmic fractions were diluted 1:1 in Clarity OTX Lysis-Loading Buffer v2.0. Then, diluted cytoplasmic fractions and nuclear fractions were diluted 1:75 in ultrapure water prior to analysis by CL-qPCR.

### CL-qPCR

Calibration curves were prepared by spiking 1.5 μL of 25 μM oligonucleotide or conjugate into 13.5 μL of ultrapure water or diluted lysate (i.e., 1:75 diluted cell lysate, 1:75 diluted nuclear lysate, or 1:150 diluted cytoplasm). Serial dilutions were carried out in ultrapure water or diluted lysate in 10-fold steps thereafter. For CL reactions, a master mix containing the following reagents per sample was prepared: 0.11 μL of 9 μM PS ligator and 50 μM TCEP HCl (Fluorochem M02624) or Bond-Breaker TCEP Solution, Neutral pH (Thermo Scientific 77720); 0.10 μL of 10 μM BPS ligator; 1.00 μL of 10X PCR Reaction Buffer with 20 mM MgCl_2_ (Roche 12032929001); 1.00 μL of 1 mg/mL PolyA (GE Healthcare 27–4110–01); and 5.79 μL of ultrapure water. The sequence of the PS ligator was 5′-d(TTAAACCAAGAAAACATTt)-3′, where the lowercase letter indicates a 3′ terminal PS group. The sequences of the BPS ligators were 5′-d(CTCAGGCTGCTAACCACGT)-3′ (BPS ligator C) and 5′-d(CTCAGGTTGCTAACCACGT)-3′ (BPS ligator T). BPS ligator (C) was used for all experiments unless indicated otherwise. Reactions containing 8 μL of master mix and 2 μL of sample were run in PCR strips (Sarstedt 72.991.002) or 96-well plates (Sarstedt 72.1978) on a C1000 Touch Thermal Cycler (Bio-Rad) or S1000 Thermal Cycler (Bio-Rad) at 33 °C for 1 h. For qPCR reactions, a master mix containing the following reagents per sample was prepared: 0.50 μL of 10 μM 5′ Cyanine 3 (Cy3)-labeled forward primer (Microsynth AG, Balgach, Switzerland), 0.50 μL of 10 μM reverse primer (Microsynth AG, Balgach, Switzerland), 2.00 μL of 5X Q5 Reaction Buffer (New England BioLabs B9027S), 0.10 μL of 2000 U/mL Q5 High-Fidelity DNA Polymerase (New England BioLabs M0491L), 0.10 μL of 25 mM dNTPs (Thermo Scientific R0181), 0.43 μL of 28.28 μM 3′ Black Hole Quencher-2 (BHQ-2)-labeled primer (Microsynth AG, Balgach, Switzerland), and 6.37 μL of ultrapure water. Reactions containing 10 μL of qPCR master mix and 2 μL of CL sample were run in 384-well plates (Life Systems Design/Starlab 4ti-0382) on a LightCycler 480 (Roche) using the following program: 95 °C for 30 s (one cycle) and 95 °C for 3 s, 50 °C for 30 s, and 72 °C for 10 s (50 cycles). The sequence of the 5′ Cy3-labeled forward primer was 5′-d(CTCCCTCCCTCGATTTAAACCAAGAAAACAT)-3′, the sequence of the reverse primer was 5′-d(ACGTGGTTAGCAGCCTGAGAA)-3′, and the sequence of the 3′ BHQ-2-labeled primer was 5′-d(AAATCGAGGGAGGGAG)-3′. Calibration curves were plotted in GraphPad Prism and analyzed by linear regression. qPCR efficiencies were calculated using the Thermo Fisher Scientific qPCR Efficiency Calculator, an online tool available at https://www.thermofisher.com/ch/en/home/brands/thermo-scientific/molecular-biology/molecular-biology-learning-center/molecular-biology-resource-library/thermo-scientific-web-tools/qpcr-efficiency-calculator.html.

### Splint Ligation-qPCR

We performed Splint ligation-qPCR according to the methods of Shin et al.^49^ Ligators were purchased from Microsynth AG (Balgach, Switzerland). The sequence of Ligator A was 5′-d(TTAAACCAAGAAAACATTT)-3′, the sequence of Ligator B was 5′-d(CTCAGGCTGCTAACCACGT)-3′, the sequence of Ligator A* was 5′-d(TTAAACCAAGAAAACATTTC)-3′, and the sequence of Ligator B* was 5′-d(TCAGGCTGCTAACCACGT)-3′. Both Ligators B and B* bear a 5′ terminal phosphate group. For Splint ligation reactions, a master mix containing the following reagents per sample was prepared: 2.00 μL of 100 nM Ligator A or A*, 2.00 μL of 100 nM Ligator B or B*, 2.00 μL of 10X SplintR ligase reaction buffer (New England BioLabs M0375), and 4.00 μL of ultrapure water. Reactions containing 10 μL of master mix and 2 μL of sample were run in PCR strips (Sarstedt 72.991.002) or 96-well plates (Sarstedt 72.1978) on a C1000 Touch Thermal Cycler (Bio-Rad) or S1000 Thermal Cycler (Bio-Rad) at 95 °C for 5 min and cooled to 33 °C. Then, 8 μL of SplintR ligase (New England BioLabs M0375) was added to achieve 2.5 U/reaction for DNA/LNA-modified oligonucleotides or 25 U/reaction for MOE-modified oligonucleotides. Ligations were carried out at 33 °C for 30 min. Reactions were heat inactivated at 65 °C for 20 min and then cooled to 20 °C. For qPCR reactions, a master mix containing the same reagents described above for CL-qPCR was prepared with the amount of ultrapure water adjusted to 4.37 μL. Reactions containing 8 μL of qPCR master mix and 4 μL of Splint ligation sample were run as described for CL-qPCR.

### Quantification of 18S gDNA and HPRT1

We quantified 18S gDNA as previously described.^40^ Briefly, a master mix containing the following reagents per sample was prepared: 0.20 μL of Hs03003631_g1 (Thermo Fisher Scientific 4331182), 1.00 μL of 25 mM MgCl_2_ (Roche 12032929001), 1.00 μL of GeneAmp 10X PCR Buffer (Applied Biosystems N8080129), 0.10 μL of 5 U/μL FastStart Taq DNA Polymerase (Roche 12032929001), 0.15 μL of 25 mM dNTPs (Thermo Scientific R0181), and 6.55 μL of ultrapure water. Reactions containing 9 μL of master mix and 1 μL of sample (i.e., 1:75 diluted cell lysate, 1:75 diluted nuclear lysate, or 1:150 diluted cytoplasm) were run in 384-well plates (Life Systems Design/Starlab 4ti-0382) on a LightCycler 480 (Roche) using the following program: 95 °C for 10 min (one cycle) followed by 95 °C for 3 s and 60 °C for 30 s (50 cycles). To quantify HPRT1, cDNA was generated from 1:75 diluted nuclear lysate and 1:150 diluted cytoplasm using the PrimeScript RT Reagent Kit (Perfect Real Time) (Takara Bio RR037A) according to the manufacturer’s instructions. The program was 37 °C for 15 min and 98 °C for 8 s. A master mix containing the following reagents per sample was prepared: 0.20 μL of Hs99999909_m1 (Thermo Fisher Scientific 4453320), 1.00 μL of 25 mM MgCl_2_ (Roche 12032929001), 1.00 μL of GeneAmp 10X PCR Buffer (Applied Biosystems N8080129), 0.10 μL of 5 U/μL FastStart Taq DNA Polymerase (Roche 12032929001), 0.15 μL of 25 mM dNTPs (Thermo Scientific R0181), and 6.55 μL of ultrapure water. Reactions containing 9 μL of master mix and 1 μL of cDNA were run in 384-well plates (Life Systems Design/Starlab 4ti-0382) on a LightCycler 480 (Roche) using the same program described above.

### Generation of FECH Minigenes and FECH-3-C-5 Stable Cell Lines

The genomic region spanning exons 3 to 5 of the human FECH gene was PCR amplified from K562 cell genomic DNA using primers comprising 5′ restriction sites for *Eco*RI and NotI, respectively (forward: CCGCATATCCTGAATTCGAAGCCGAAAACTGGAATATTAATGCTAAAC, reverse: CAGGATATGCGGGCGGCCGCCTGTGGTGGAGCAGCTGTAC, TA = 59 °C, extension: 3:30). The PCR product and target vector (pCI-neo, Promega E1841) were digested using *Eco*RI and NotI (New England Biolabs). The vector was dephosphorylated with Shrimp Alkaline Phosphatase (New England Biolabs) to reduce self-ligation in accordance with the manufacturer’s protocol. The insert and vector were ligated and transformed into NEB 5-α Competent *Escherichia coli* (High Efficiency). Plasmids with a successfully integrated FECH minigene were fully sequenced by Oxford Nanopore sequencing (plasmidsaurus). The resulting plasmid was designated as the FECH-T minigene plasmid (Figure S26A). The EPP-associated c.315-48C polymorphism was introduced using the Q5 Site-Directed Mutagenesis Kit (New England Biolabs) and mutagenic PCR primers (forward: ATTTCTCAGGCTGCTAAGCTG, where the underlined letter indicates the mutagenesis site; reverse: GTTTTCTACTCAATAAAAAAGAAAAAAAG). Selection and production of the final product were conducted as described above, yielding the FECH-C minigene plasmid (Figure S26A).

Stable transfection of K562 cells was performed based on the Technical Reference Guide “Guideline for Generation of Stable Cell Lines” by Lonza.^50^ Briefly, K562 cells were kept at 37 °C with 5% CO_2_ in a humidified incubator. Cells were seeded at approximately 1 × 10^5^ cells/mL in IMDM (Sigma-Aldrich) containing 10% FBS (Thermo Fisher) and 1% GlutaMax (Thermo Fisher). At 1 × 10^6^ cells/mL, cells were transfected with the FECH-C minigene plasmid using Lipofectamine 2000 (Thermo Fisher) according to the manufacturer’s specifications and given 48 h to recover before being subjected to selection media. Untransfected cells were also seeded, serving as the kill curve to determine suitable selection conditions. For both transfected and untransfected cells, selection media contained Geneticin G418 Sulfate (powder; potency: 721 μg/mg, Thermo Fisher) at the following concentrations: 0 mg/mL (control), 0.4, 0.6, 0.8, 1.0, and 1.2 mg/mL. G418 was freshly prepared and sterile-filtered (Whatman syringe filter, FP 30/0.2, 0.2 μm) prior to each use. G418-containing media were exchanged every 2–3 days, and cell viability was monitored via trypan blue staining in a Countess 3 Automated Cell Counter (Invitrogen). Kill curve cells were discarded at day 11, when a suitable selection condition was determined. Cells seeded in ≥0.6 mg/mL G418 were maintained in selection media. At day 16 in the selection medium, cells in 1.2 mg/mL G418 were transferred into selection medium containing 1.0 mg/mL G418. At day 22, the cells were frozen in Recovery Cell Culture Freezing Medium (Thermo Fisher). For expedience, no clonal selection was performed and the cells were used as batch culture. The cell line was designated as K562 FECH-3-C-5.

The generation of a corresponding 293 cell line was performed analogously with minor adjustments for adherent cells. 293 cells were kept at 37 °C with 5% CO_2_ in a humidified incubator. Cells were seeded at 10% confluency in DMEM (DMEM + GlutaMax; Thermo Fisher) and 10% FBS (Thermo Fisher). They were passaged approximately every 2–3 days at around 100% confluency. Cells were transfected at approximately 80% confluency with the FECH-C minigene plasmid using Lipofectamine 2000 according to the manufacturer’s specifications and given 24 h to recover before being subjected to selection media. Untransfected cells were also seeded, serving as the kill curve to determine suitable selection conditions. For both transfected and untransfected cells, selection media contained G418 at the following concentrations: 0 mg/mL (control), 0.2, 0.4, 0.6, 0.8, 1.0, 1.2, and 1.4 mg/mL. G418 was freshly prepared and sterile-filtered prior to each use. G418-containing media were exchanged every 2–3 days without detaching the cells, and cell viability was followed visually (Figure S26C). Kill curve cells were discarded at day 9, when a suitable selection condition was determined. Cells seeded in ≥0.8 mg/mL G418 were reseeded in a bigger volume and maintained in selection media. At day 19, the different conditions were separately transferred into selection medium at 1.0 mg/mL G418 to allow for faster growth. At day 27, cells initially kept in 1.4 mg/mL G418 were frozen in Recovery Cell Culture Freezing Medium (Thermo Fisher). The cell line was designated as 293 FECH-3-C-5.

### Splice-Switching Assays

Splice-switching assays were carried out in K562 FECH-3-C-5 and 293 FECH-3-C-5 cells. For free uptake experiments, cells were counted as described above and seeded in 24-well plates (Techno Plastic Products 92024) at 115,000 live cells per mL and 500 μL per well in complete medium. After 3–4 h, treatments were performed by diluting oligonucleotides or conjugates in 50 μL of Opti-MEM (Gibco 31985047) and directly adding the diluted oligonucleotides or conjugates to the cells. At 48 h post-treatment, K562 cells were transferred to clean tubes and pelleted by centrifugation. Supernatants were discarded, and the cells were lysed and homogenized in 500 μL of TRIzol Reagent (Ambion 15596018) prior to storage at −20 °C. At 48 h post-treatment, 293 cells were lysed and homogenized in 500 μL of TRIzol Reagent (Ambion 15596018), and then the samples were transferred to clean tubes prior to storage at −20 °C. All samples were thawed at ambient temperature. Following the addition of 100 μL of cold chloroform, samples were incubated for 1 min at ambient temperature, vortexed, and then centrifuged at 12,000*g* and 4 °C for 15 min. The aqueous phase was transferred to a clean tube, to which one volume of ethanol was added. The samples were then loaded onto Zymo-Spin IC Columns (Zymo Research C1004-50) and centrifuged at 16,000*g* and ambient temperature for 1 min. Samples were washed twice with 80% (v/v) ethanol, 100 mM NaCl, 10 mM Tris, pH 7.5, and eluted in 40–50 μL ultrapure water at ambient temperature or 60 °C. Absorbance at 260 nm was measured as described above. cDNA was generated using the PrimeScript RT Reagent Kit (Perfect Real Time) (Takara Bio RR037A) according to the manufacturer’s instructions. The program was 37 °C for 15 min and 98 °C for 8 s. PCR of FECH transcripts was performed using 9 μL of GoTaq Green Master Mix (Promega M712C) according to the manufacturer’s instructions with the addition of 1 mM MgCl_2_ and 1 μL of cDNA in PCR strips (Sarstedt 72.991.002) on a C1000 Touch Thermal Cycler (Bio-Rad) or S1000 Thermal Cycler (Bio-Rad) using the following program: 95 °C for 2 min (one cycle); 95 °C for 30 s, 69 °C for 30 s, and 72 °C for 30 s (25–30 cycles); and 72 °C for 5 min. PCR primers amplifying the FECH exon 3-exon 4 junction were purchased from Microsynth AG (Balgach, Switzerland). The sequence of the forward primer was 5′-d(GGACCGAGACCTCATGACACTTCC)-3′, and the sequence of the reverse primer was 5′-d(TTCATCCAGCAGCTTCACCATGC)-3′.

For transfection experiments, 293 cells were counted as described above and seeded in 6-well plates (Techno Plastic Products 92006) at 300,000 live cells per mL and 2 mL per well in complete medium. After 24 h, the cells were transfected with 10 nM of oligonucleotide or conjugate using Lipofectamine 2000 Reagent (Invitrogen 11668019) according to the manufacturer’s instructions for RNA transfection. At 48 h post-transfection, the cells were lysed and homogenized in 1 mL of TRIzol Reagent (Ambion 15596018), and then the samples were transferred to clean tubes. Samples were stored at −20 °C and thawed at ambient temperature. Following the addition of 200 μL of cold chloroform, the samples were incubated for 1 min at ambient temperature, vortexed, and then centrifuged at 12,000*g* and 4 °C for 15 min. The aqueous phase was transferred to a clean tube to which one volume of ethanol was added. The samples were then loaded onto Zymo-Spin IIICG Columns (Zymo Research C1006-50-G) and centrifuged at 16,000*g* and ambient temperature for 1 min. Samples were washed twice with 80% (v/v) ethanol, 100 mM NaCl, 10 mM Tris, pH 7.5, and eluted in 100 μL ultrapure water at ambient temperature or 60 °C. Absorbance measurements, cDNA generation, and PCR were performed as described above.

For free uptake experiments with chloroquine, we followed the protocol of Du Rietz et al.^45^ Briefly, 293 cells were counted as described above and seeded in 24-well plates (Techno Plastic Products 92024) at 200,000 live cells per mL and 500 μL per well in complete medium. Initially, treatments were performed after 3–4 h; to increase the RNA yield in subsequent experiments, we treated cells after 18–20 h. Treatments were performed by diluting oligonucleotides or conjugates in 50 μL of Opti-MEM (Gibco 31985047) and directly adding the diluted oligonucleotides or conjugates to the cells. After 24 h, the cells were washed with PBS (Gibco 10010015) and then treated with 60 μM chloroquine diphosphate salt (Sigma-Aldrich C6628) prepared in complete medium. At 24 h post-chloroquine treatment, the cells were washed with PBS and lysed and homogenized in 500 μL of TRIzol Reagent (Ambion 15596018). The samples were transferred to clean tubes, stored at −20 °C, and thawed at ambient temperature. Following the addition of 100 μL of cold chloroform, samples were incubated for 1 min at ambient temperature, vortexed, and centrifuged at 12,000*g* and 4 °C for 15 min. The aqueous phase was transferred to a clean tube to which one volume of ethanol was added. The samples were then loaded onto Zymo-Spin IC Columns (Zymo Research C1004-50) and centrifuged at 16,000*g* and ambient temperature for 1 min. Samples were washed twice with 80% (v/v) ethanol, 100 mM NaCl, 10 mM Tris, pH 7.5, and eluted in 40–50 μL ultrapure water at ambient temperature or 60 °C. Absorbance measurements, cDNA generation, and PCR were performed as described above.

For all experiments, PCR reactions (9–9.5 μL) were directly loaded onto a 2% (w/v) agarose (Invitrogen 16500-500) gel stained with GelRed Nucleic Acid Stain, 10,000X in Water (Biotium 41003) and run in 1X Tris-acetate-EDTA (TAE) electrophoresis buffer (Thermo Scientific B49) at 150 V and ambient temperature for 1.5 h. The ladder was 5 μL of Quick-Load Purple 100 bp DNA Ladder (New England BioLabs N0551S). DNA was visualized by UV transillumination on a GelDoc XR System (Bio-Rad), and bands were quantified in ImageJ.^51^

## Results and Discussion

### Synthesis and Characterization of Oligonucleotides

**Figure 1:**
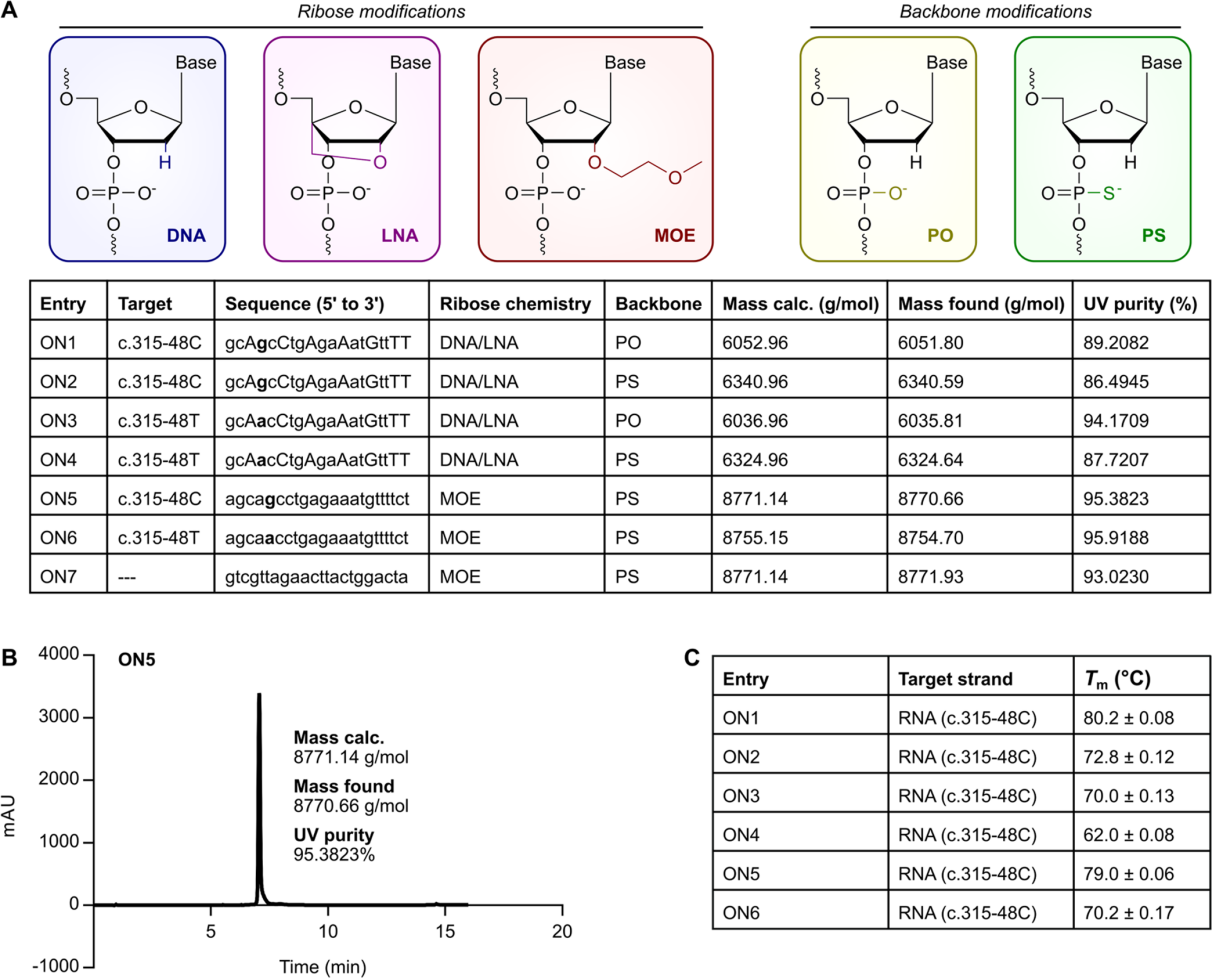
Synthesis and characterization of oligonucleotides. (A) Top, The chemical modifications employed in this study. Modifications are grouped according to their location: the three leftmost modifications occur at the 2′ position of the ribose, while the two rightmost modifications occur at the internucleotide linkage or backbone. Bottom, Table of oligonucleotides synthesized in this study. In sequences with DNA/LNA ribose chemistry, lowercase letters are DNA, and uppercase letters are LNA. The nucleotide in bold is complementary to the targeted SNP. Masses were calculated using the Oligowizard Nucleic Acid Calculator, an online tool available at http://oligowizard.com/. ON, oligonucleotide; DNA, deoxyribonucleic acid; LNA, locked nucleic acid; MOE, 2′-*O*-methoxyethyl; PO, phosphodiester; and PS, phosphorothioate. (B) Representative LC–MS chromatogram. In (A) and (B), UV purity is expressed as the percent area under the peak. (C) *T*_m_s for the oligonucleotides paired with a 22-nt RNA representing the c.315-48C FECH pre-mRNA. Data are mean ± standard deviation (SD) for three technical replicates. *T*_m_, melting temperature.

For our study, we selected SSOs previously developed to treat EPP.^35,36^ Oligonucleotide (ON) targets, sequences, and chemistries are listed in Figure 1A. ON1–4 were adapted from Oustric et al.,^35^ and ON5–7 are from Halloy et al.^36^ ON1, ON2, and ON5 target the EPP-associated c.315-48C polymorphism, while ON3, ON4, and ON6 target the healthy c.315-48T polymorphism. ON7 is a negative control molecule with the same nucleotide composition as ON5, but the sequence is scrambled. As per Oustric et al.,^35^ we synthesized ON1–4 with mixed DNA and LNA ribose chemistries. However, we changed the ribose chemistry at the 3′ nucleotide from DNA to LNA for increased nuclease stability.^19^ DNA/LNA oligonucleotides were synthesized with phosphodiester (PO) or phosphorothioate (PS) backbones. As per Halloy et al.,^36^ we synthesized ON5–7 with full MOE ribose chemistry and PS backbones. Oligonucleotide masses and purities are listed in Figure 1A. A representative liquid chromatography–mass spectrometry (LC–MS) chromatogram is provided in Figure 1B; all chromatograms are provided in Figure S1.

To measure the oligonucleotides’ RNA target-binding affinities, we performed UV melting. As a target, we used a 22-nt RNA representing the c.315-48C FECH pre-mRNA. Melting data are provided in Figure S2. As expected, the oligonucleotides showed distinct melting temperatures (*T*_m_s; Figure 1C). Specifically, the *T*_m_s spanned the range 62.0 to 80.2 °C, or over 18 °C (Figure 1C). For oligonucleotides with DNA/LNA ribose chemistry, a change from a PO backbone to a PS backbone reduced the *T*_m_ by an average of 7.7 °C (Figure 1C). The average Δ*T*_m_ of −0.43 °C per PS modification is consistent with the Δ*T*_m_ of −0.5 °C per PS modification reported for several uniformly modified sequences.^20^ Additionally, for oligonucleotides with DNA/LNA ribose chemistry, a change from a fully complementary duplex to a duplex with a mismatch at a DNA:RNA base pair reduced the *T*_m_ by an average of 10.5 °C (Figure 1C). For oligonucleotides with MOE ribose chemistry, the same mismatch at an MOE:RNA base pair reduced the *T*_m_ by 8.8 °C (Figure 1C). This value falls within the expected range for MOE:RNA mismatches (i.e., Δ*T*_m_ of −3.6 to −9.2 °C per mismatch).^17^ Of note, the c.315-48C-targeting MOE PS ON5 displayed a higher *T*_m_ than the c.315-48C-targeting DNA/LNA PS ON2 (Figure 1C).

### Detection of Oligonucleotides Using CL-qPCR

**Figure 2:**
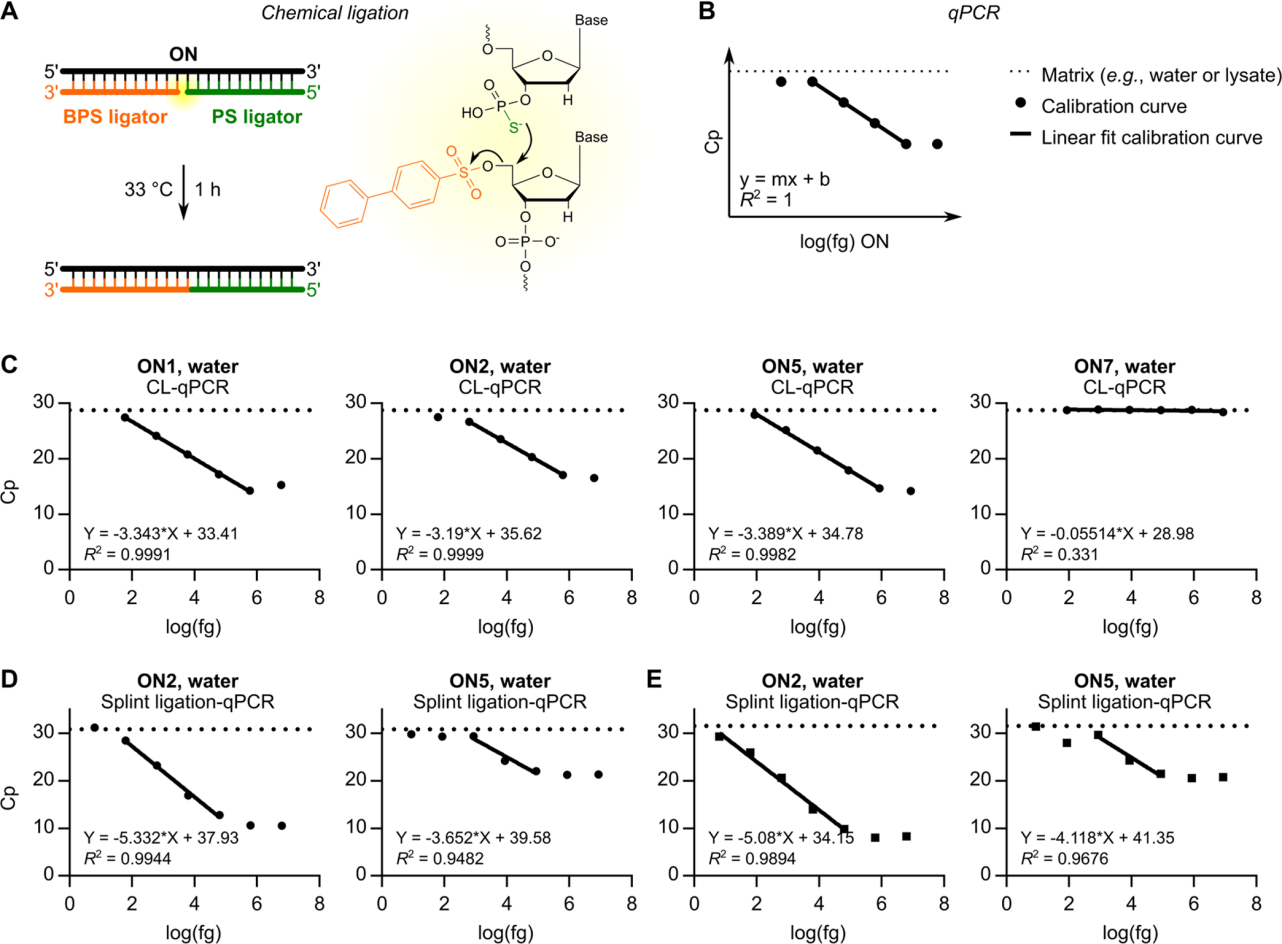
Principle of CL-qPCR and comparison to Splint ligation-qPCR. (A) In the first step, two DNA ligators hybridize to an ON of interest and are ligated. (B) In the second step, the newly ligated DNA molecule serves as a template for qPCR. The equation of a linear fit calibration curve, which relates Cp values with known amounts of an ON in a given matrix, is used to calculate an unknown amount of the ON in the same matrix. (C) Linear fit CL-qPCR calibration curves for selected oligonucleotides in water. (D) Linear fit Splint ligation-qPCR calibration curves for selected oligonucleotides in water using DNA ligators A and B. (E) Linear fit Splint ligation-qPCR calibration curves for selected oligonucleotides in water using redesigned DNA ligators A* and B*. In parts C–E, data are mean Cp values ± SD for three technical replicates.

To quantify the oligonucleotides in cells and subcellular compartments, we employed CL-qPCR.^36,40,41^ This technique consists of two steps (Figure 2A,B).^36,40^ In the first step, a DNA strand with a 5′ biphenylsulfonyl (BPS) group (i.e., a BPS ligator) and an equimolar amount of a second DNA strand bearing a 3′ PS group (i.e., a PS ligator) is added to a sample containing an ON of interest. The ligators hybridize to the ON by base complementarity, whereupon they are ligated after the nucleophilic displacement of the BPS group by the terminal PS group (Figure 2A). In the second step, the newly ligated DNA molecule serves as a template for hybridization probe qPCR (Figure 2B). Importantly, a calibration curve is generated by diluting an ON in a given matrix over a concentration range and performing CL-qPCR. Using the equation of the linear fit calibration curve, an unknown amount of ON in the same matrix can then be calculated (Figure 2B).

We first performed CL-qPCR for ON1–7 diluted in water in 10-fold steps over the concentration range of 83 nM to 0.83 pM. We used a BPS ligator with the sequence 5′-CTCAGGCTGCTAACCACGT-3′ (BPS ligator (C)), where the underlined nucleotide is complementary to the SNP-targeting nucleotide of ON1, ON2, and ON5 (Figure 1A). We used a PS ligator with the sequence 5′-TTAAACCAAGAAAACATTT-3′. LC–MS chromatograms for the chemical ligators are listed in Figure S3. The upper limit of detection in this assay was approximately 17 nM, which reflects the concentration of each ligator (BPS and PS) in the reaction. Linear fit CL-qPCR calibration curves for ON1, ON2, and ON5 are shown in Figure 2C. A linear fit CL-qPCR calibration curve for ON7, which has a scrambled sequence, is also shown in Figure 2C. All curves are provided in Figure S4. For the DNA/LNA PO ON1 and MOE PS ON5, the linear range spanned 4 orders of magnitude, from the highest detectable concentration of 8.3 nM to the lowest tested concentration of 0.83 pM (*R*^2^ > 0.99; Figure 2C). For the DNA/LNA PS ON2, the linear range spanned 3 orders of magnitude, from 8.3 nM to 8.3 pM (*R*^2^ = 0.9999; Figure 2C). ON7 was not detected at any concentration tested (Figure 2C).

We then performed the same assay using a BPS ligator with the sequence 5′-CTCAGGTTGCTAACCACGT-3′ (BPS ligator (T)), where the underlined nucleotide is complementary to the SNP-targeting nucleotide of ON3, ON4, and ON6 (Figure 1A). Linear fit CL-qPCR calibration curves are provided in Figure S5, and a comparison of results using the different BPS ligators is provided in Figure S6. As expected, the detection of ON1, ON2, and ON5 was better than the detection of ON3, ON4, and ON6, respectively, using the BPS ligator (C), because this ligator forms a Watson–Crick base pair (G:C) with the former oligonucleotides but a mismatch (A:C) with the latter oligonucleotides at the SNP site (Figure S6). On the other hand, the detection of ON1, ON2, and ON5 was similar to the detection of ON3, ON4, and ON6, respectively, using the BPS ligator (T), because this ligator forms a wobble base pair (G:T) with the former oligonucleotides and a Watson–Crick base pair (A:T) with the latter oligonucleotides at the SNP site (Figure S6). Indeed, rG:dT wobble pairs are particularly stable;^52^ the affinity of an ASO for an off-target RNA with an rG:dT pair may be similar to the affinity of the ASO for its intended RNA target.^53^

### Comparison of CL-qPCR to Splint Ligation-qPCR

Next, we compared CL-qPCR to a technique for oligonucleotide quantification that relies on an enzymatic, rather than a chemical, ligation step. The technique, which was developed by Shin et al.,^49^ utilizes SplintR ligase and thus is known as Splint ligation-qPCR. We started by using a DNA molecule with the sequence 5′-TTAAACCAAGAAAACATTT-3′ (ligator A) and a DNA molecule with a 5′ phosphate group with the sequence 5′-CTCAGGCTGCTAACCACGT-3′ (ligator B), which are identical to the PS ligator and BPS ligator (C) sequences, respectively, used in our CL-qPCR experiments. The enzymatic ligation step was performed according to the methods of Shin et al.,^49^ where 10-fold more SplintR ligase was used to detect MOE oligonucleotides relative to DNA/LNA oligonucleotides. The upper limit of detection in this assay was approximately 3.3 nM, which reflects the concentration of each ligator (A and B) in the reaction. Because the sensitivity of the technique for the oligonucleotides was unclear, we performed Splint ligation-qPCR for ON2 and ON5 diluted in water in 10-fold steps over the concentration range of 83 nM to 83 fM, which extended the concentration range used in our CL-qPCR experiments by an order of magnitude at the low end. Linear fit Splint ligation-qPCR calibration curves for ON2 and ON5 are shown in Figure 2D. Notably, the linear range for ON2 spanned 3 orders of magnitude, from the highest detectable concentration of 0.83 nM to 0.83 pM (*R*^2^ = 0.9944; Figure 2D), indicating a higher sensitivity for the DNA/LNA PS ON2 using Splint ligation-qPCR relative to CL-qPCR. However, the sensitivity for the MOE PS ON5 was worse: the linear range spanned only 2 orders of magnitude, from 0.83 nM to 8.3 pM (*R*^2^ = 0.9482; Figure 2D).

Notably, Shin et al. observed that SplintR ligase is less efficient for MOE oligonucleotides.^49^ To compensate for this loss of efficiency, the authors used 10-fold more enzyme to detect MOE oligonucleotides, as noted above. Additionally, the manufacturer’s information for SplintR ligase notes that while the enzyme tolerates all base pairs at the ligation junction, it is partially inhibited by C/G base pairs at the phosphorylated side of the junction, particularly when there is a C/G base pair two positions downstream. We reasoned the combined effect of MOE chemistry, a C:G base pair at the ligation junction, and a C:G base pair two positions downstream of the ligation junction put ON5 at a significant disadvantage of being detected by Splint ligation-qPCR. To overcome the latter two limitations, we redesigned our ligators A and B such that the ligation junction was shifted by one base pair. We used a DNA molecule with the sequence 5′-TTAAACCAAGAAAACATTTC-3′ (ligator A*) and a DNA molecule with a 5′ phosphate group with the sequence 5′-TCAGGCTGCTAACCACGT-3′ (ligator B*). Linear fit Splint ligation-qPCR calibration curves for ON2 and ON5 using these redesigned ligators are shown in Figure 2E. Under these conditions, the sensitivity for the DNA/LNA PS ON2 was even better: the linear range covered 4 orders of magnitude, from 0.83 nM to the lowest tested concentration of 83 fM (*R*^2^ = 0.9894; Figure 2E). However, the sensitivity for the MOE PS ON5 was not improved: the linear range still spanned only 2 orders of magnitude, from 0.83 nM to 8.3 pM (*R*^2^ = 0.9676; Figure 2E). We concluded that while Splint ligation-qPCR is superior to CL-qPCR at detecting DNA/LNA PS-modified oligonucleotides, CL-qPCR is superior to Splint ligation-qPCR at detecting MOE PS-modified oligonucleotides, at least with these sequences and under these conditions.

### Quantification of Oligonucleotides in Cells and Subcellular Compartments Following Free Uptake

**Figure 3:**
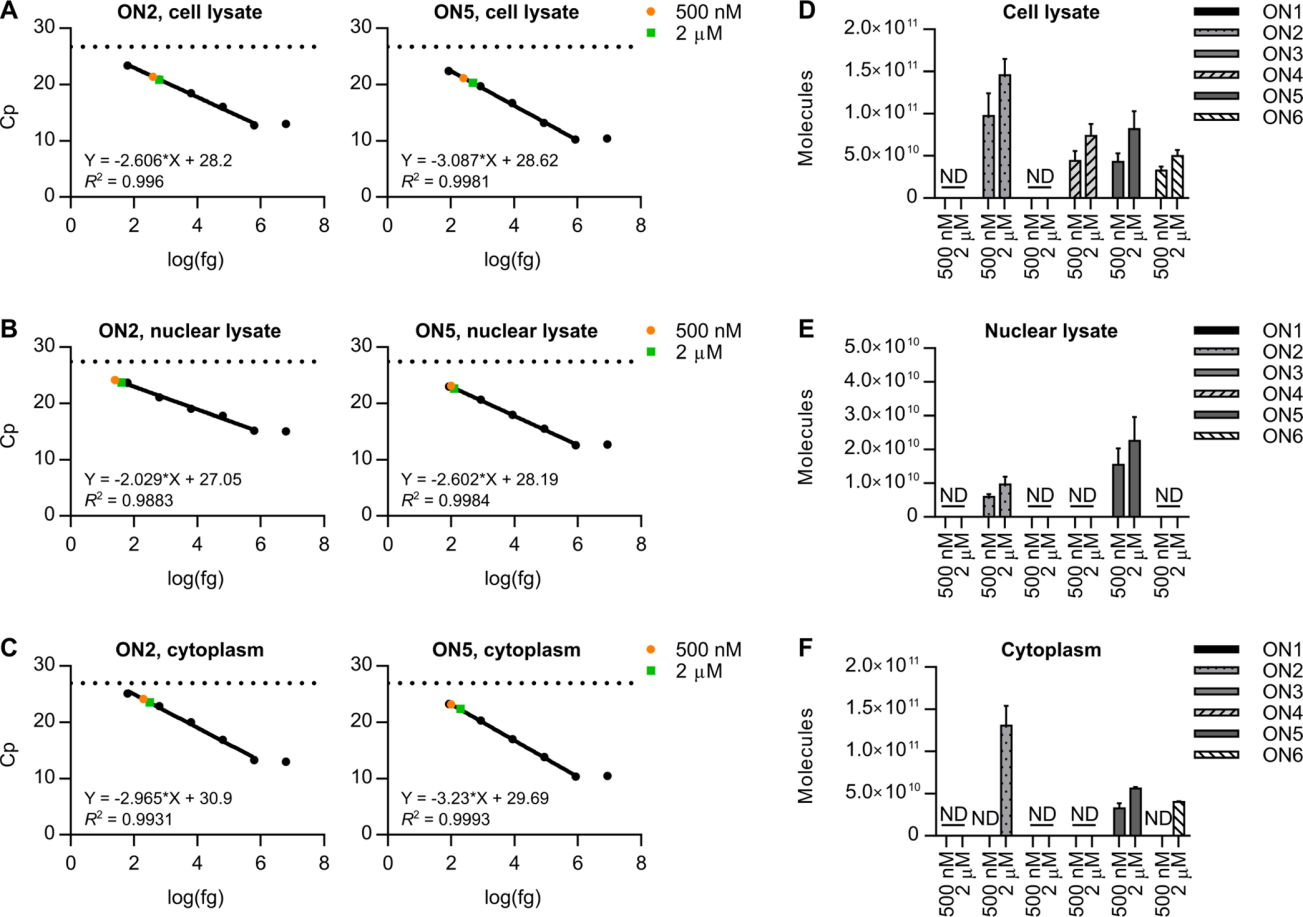
Cellular uptake and subcellular localization of oligonucleotides. (A–C) Linear fit CL-qPCR calibration curves for selected oligonucleotides in HEL (A) cell lysate, (B) nuclear lysate, and (C) cytoplasm. The Cp values measured in each compartment following free uptake for 24 h are overlaid. Data are mean Cp values ± SD for three biological replicates (*n* = 3). (D–F) Oligonucleotides detected in HEL (D) cell lysate, (E) nuclear lysate, and (F) cytoplasm following free uptake for 24 h. Data are mean molecules ± standard error of the mean (SEM) for three biological replicates (*n* = 3). ND, not detected.

Next, we measured the cellular uptake and subcellular localization of the oligonucleotides using CL-qPCR. For our experiments, we used the HEL cell line, which are erythroblast cells isolated from the bone marrow of a patient with erythroleukemia.^54^ Pendergraff et al. were unable to detect DNA/LNA PS ASOs in the nucleus of HeLa cells treated under free uptake conditions at 200 nM even for 72 h.^34^ Consistent with these results, Deprey et al. showed that the nuclear penetration of MOE PS ASOs requires approximately 600 nM and 24 h.^55^ We treated HEL cells with ON1–6 under free uptake conditions at either 500 nM or 2 μM. At 24 h, the cells were lysed or fractionated into subcellular compartments (nucleus, cytoplasm) using the Rapid, Efficient, And Practical (REAP) method^48^ with minor changes. Namely, prior to lysis of the cellular membrane, the cells were washed with PBS and 0.1 mg/mL heparin to remove any oligonucleotides bound to the cellular membrane but not internalized within the cells. Then, we performed CL-qPCR on HEL cell lysate, nuclear lysate, and cytoplasm (Figures 3A–C, S7–S9) and quantified the molecules in each compartment (Figures 3D–F, S10, Tables S1–3). Notably, the oligonucleotides with PO backbones were not detected in any compartment, suggesting that these oligonucleotides are not taken up by cells and/or are degraded by nucleases in the extracellular or intracellular environment.

In cell lysate, we detected an average of 9.73 × 10^10^ and 1.46 × 10^11^ ON2 molecules and 4.31 × 10^10^ and 8.17 × 10^10^ ON5 molecules following free uptake at 500 nM and 2 μM, respectively (Table S1). Thus, up to 0.06% of all ON2 molecules and 0.03% of all ON5 molecules applied to the cells were inside the cells after 24 h (Table S1). Assuming a cell number doubling rate of 24 h,^54,56^ we calculated an average of 846,000 and 1,270,000 ON2 molecules per cell and 375,000 and 710,000 ON5 molecules per cell following free uptake at the 500 nM and 2 μM concentrations, respectively (Table S1). In nuclear lysate, we detected an average of 5.93 × 10^9^ and 9.70 × 10^9^ ON2 molecules and 1.54 × 10^10^ and 2.25 × 10^10^ ON5 molecules following free uptake at 500 nM and 2 μM, respectively (Table S2). Thus, up to 7% of all ON2 molecules and 36% of all ON5 molecules inside the cells could be accounted for in the nuclear compartment after 24 h (Table S2). These values are consistent with previous reports placing between 4 and 25% of total cell-associated PS ASOs in the nucleus following free uptake.^15,33^ Again, assuming a doubling rate of 24 h,^54,56^ we calculated an average of 52,000 and 84,000 ON2 molecules per nucleus and 134,000 and 196,000 ON5 molecules per nucleus following free uptake at the 500 nM and 2 μM concentrations, respectively (Table S2). These values are consistent with the average 122,000 to 210,000 DNA/LNA PS ASOs per nucleus determined by quantitative fluorescence imaging following free uptake in MCF-7 cells at 5 μM for 24 to 72 h.^33^ The values are also consistent with the average 30,000 to 308,000 DNA/LNA PS ASOs per nucleus determined by subcellular fractionation, nucleus counting, and sandwich enzyme-linked immunosorbent assay (ELISA) following free uptake in HeLa cells at 1 to 15 μM for 72 h.^34^ Data for the cytoplasmic compartment are reported in Table S3.

To account for variation in terms of the number of cells or nuclei recovered across treatments and to confirm subcellular fractionation, we measured 18S genomic DNA (gDNA) as previously described^40^ as well as the cytoplasmic marker hypoxanthine phosphoribosyltransferase (HPRT1; Figure S11). 18S gDNA signals were similar in the cell and nuclear lysate across the different treatments (Figure S11A,B), indicating a similar recovery. Additionally, 18S gDNA signals were relatively stronger in nuclear fractions than in cytoplasmic fractions (Figure S11B), and HPRT1 signals were relatively stronger in cytoplasmic fractions than in nuclear fractions (Figure S11C), confirming successful subcellular fractionation. Normalized cellular uptake and nuclear localization data are listed in Figure S12.

### Conjugation of NLS Peptides to a Lead Oligonucleotide

**Figure 4:**
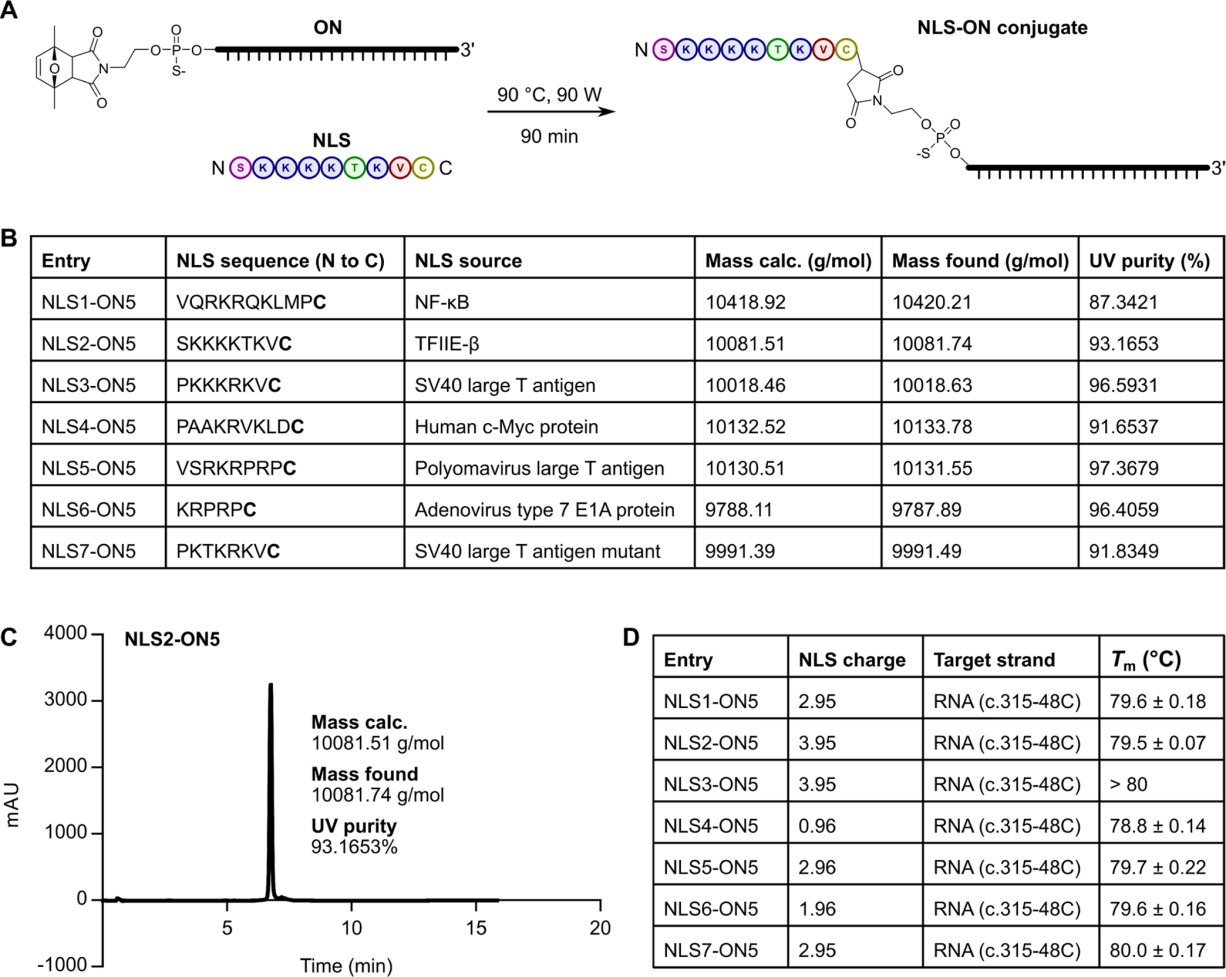
Preparation and characterization of NLS peptide–oligonucleotide conjugates. (A) Conjugate preparation scheme. Reactions were performed postsynthetically and in solution under microwave irradiation at 90 °C and 90 W for 90 min using one mol equivalent of 5′-capped-maleimide-modified oligonucleotide and six mol equiv of peptide in 8 mM TEAA buffer, pH 6.8. (B) Table of conjugates prepared for this study. Peptides were purchased from GenScript (Piscataway, New Jersey) with N-terminal acetylation. A C-terminal C amino acid (bold) was added to each peptide sequence for the thiol–maleimide conjugation. The mass of the 5′-capped-maleimide-modified oligonucleotide was calculated using the Oligowizard Nucleic Acid Calculator, an online tool available at http://oligowizard.com/. The mass of each peptide was provided by GenScript. (C) Representative LC–MS chromatogram. In parts (B) and (C), UV purity is expressed as the percent area under the peak. (D) *T*_m_s for the conjugates paired with a 22-nt RNA representing the c.315-48C FECH pre-mRNA. NLS charge refers to the peptide’s predicted net charge at pH 7.0 and was calculated using the Bachem peptide calculator, an online tool available at https://www.bachem.com/knowledge-center/peptide-calculator/. The upper baseline for NLS3-ON5 and the RNA target was too short to fit; therefore, a *T*_m_ of >80 °C was estimated from the maximum of the first derivative of the raw data.

To access NLS peptide–oligonucleotide conjugates, we employed a postsynthetic, solution phase thiol–maleimide conjugation protocol recently exploited by our laboratory to generate a variety of cationic peptide–oligonucleotide conjugates (Figure 4A).^47^ We selected the NLS sequences in Figure 4B. NLS1–6 derive from various sources (Figure 4B).^42,43^ NLS7 is a negative control molecule that shares its sequence with NLS3, except NLS7 bears a single K to T amino acid substitution that abolishes its nuclear localization (Figure 4B).^42^ As a reactive tag for conjugation, we added a C-terminal C amino acid to each NLS sequence (Figure 4B). The C residue provides the nucleophile that reacts with the electron-deficient carbon–carbon double bond of the maleimide.^57^ As a lead oligonucleotide, we selected the c.315-48C-targeting MOE PS ON5, as this molecule displayed a higher RNA target-binding affinity (Figure 1C) and trended toward higher nuclear accumulation (Figures 3E, S10, Table S2) relative to the DNA/LNA PS ON2. For conjugation, we prepared a 5′-capped-maleimide-modified ON5 using a commercially available building block as previously described.^47^ Conjugate masses and purities are listed in Figure 4B. A representative LC–MS chromatogram is provided in Figure 4C; all chromatograms are provided in Figure S13.

To determine the RNA target-binding affinities of the NLS peptide–oligonucleotide conjugates, we performed UV melting using the same 22-nt RNA target described above. Melting data are provided in Figure S14. The conjugates’ *T*_m_s were similar to one another and the parent SSO. Specifically, the conjugates’ *T*_m_s spanned the range 78.8 to >80 °C (Figure 4D), while the *T*_m_ of ON5 was 79.0 °C (Figure 1C). Notably, random error in *T*_m_ is usually between 1 and 2 °C.^58^ Cationic peptide–oligonucleotide conjugates are expected to have higher *T*_m_s than their parent oligonucleotides, presumably due to charge neutralization of the phosphodiester backbone.^59^ Although we did not observe an increase in *T*_m_ for any conjugate paired with the RNA target relative to the parent SSO paired with the RNA target, we note that our peptides are relatively short, ranging in length from six to 11 amino acids, and weakly charged, with predicted net charges at pH 7.0 in the range 0.96 to 3.95 (Figure 4D).^47,59^ We concluded that the selected peptides, when covalently conjugated to ON5, do not alter its affinity for its RNA target, at least under these conditions.

### Quantification of Conjugates in Cells and Subcellular Compartments Following Free Uptake

**Figure 5:**
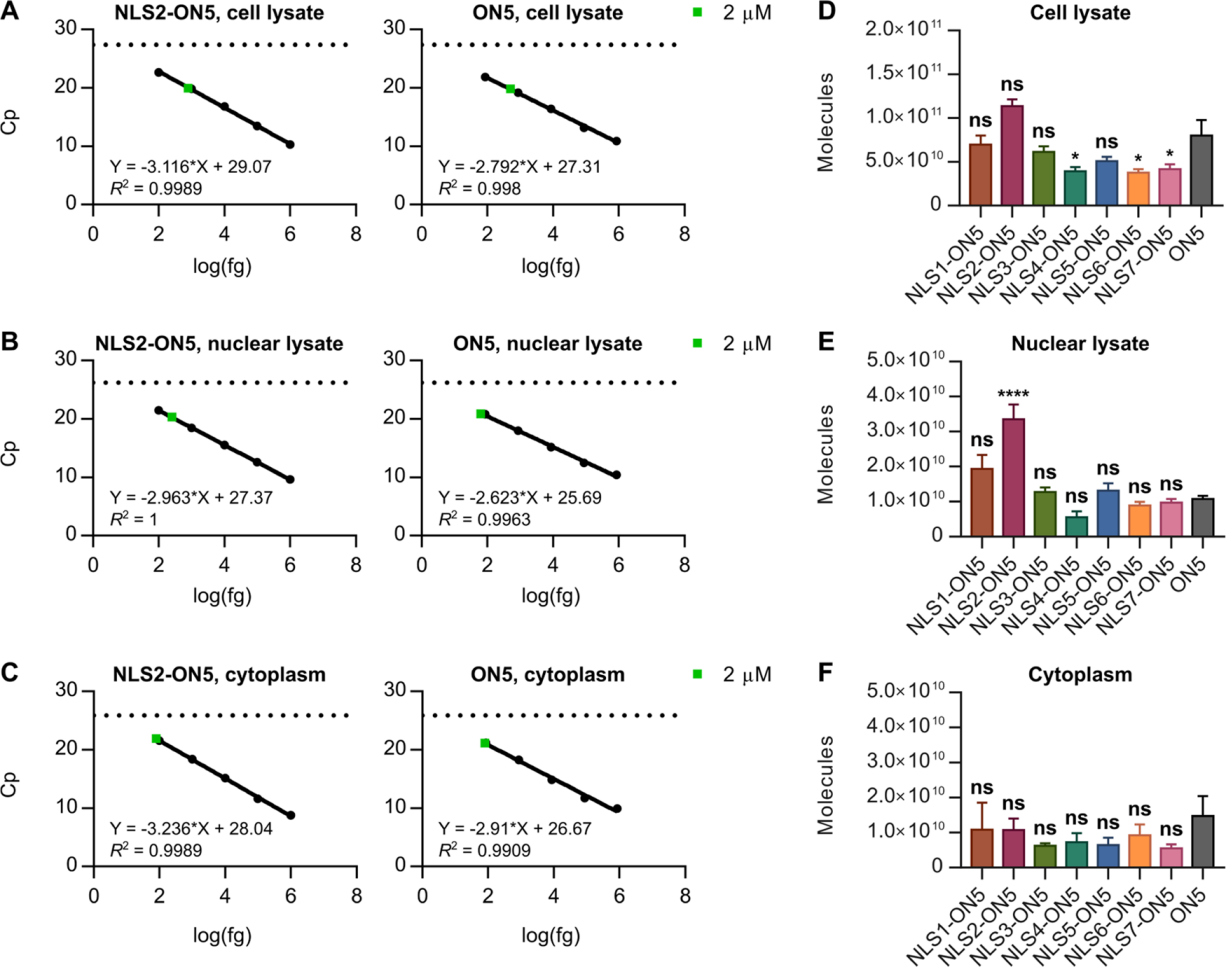
Cellular uptake and subcellular localization of conjugates. (A–C) Linear fit CL-qPCR calibration curves for a selected conjugate and ON5 in HEL (A) cell lysate, (B) nuclear lysate, and (C) cytoplasm. The Cp values measured in each compartment following free uptake at 2 μM for 24 h are overlaid. Data are mean Cp values ± SD for three biological replicates (*n* = 3). (D–F) Oligonucleotide conjugates detected in the HEL (D) cell lysate, (E) nuclear lysate, and (F) cytoplasm following free uptake at 2 μM for 24 h. HEL cell and nuclear lysate data are mean molecules ± SEM for three biological replicates (*n* = 3). HEL cytoplasm data are mean molecules ± SEM for two or three biological replicates (*n* = 2 or *n* = 3). Statistics are one-way analysis of variance (ANOVA) with Dunnett’s multiple comparisons test against ON5, α = 0.05: ns, not significant; * *P* ≤ 0.05; ** *P* ≤ 0.01; *** *P* ≤ 0.001; and **** *P* ≤ 0.0001.

Next, we measured the cellular uptake and subcellular localization of the conjugates using CL-qPCR. We first confirmed the conjugates’ compatibility with the technique (Figure S15). Then, we treated HEL cells with the conjugates under free uptake conditions at 2 μM. We included ON5 in our treatments for reference. At 24 h, the cells were lysed or fractionated as described above. We performed CL-qPCR on HEL cell lysate, nuclear lysate, and cytoplasm (Figures 5A–C, S16–S18) and quantified the molecules in each compartment (Figures 5D–F, S19, Tables S4–S6). Interestingly, we detected less NLS4-ON5, NLS6-ON5, and NLS7-ON5 than ON5 in cell lysate (*P* ≤ 0.05; Figures 5D, S19), indicating that NLS4, NLS6, and NLS7 hinder the cellular uptake of ON5. None of the conjugates was better taken up by cells relative to ON5, although on average, we detected more NLS2-ON5 molecules than ON5 molecules in cell lysate (Table S4). In nuclear lysate, we detected an average of 3.35 × 10^10^ NLS2-ON5 molecules and 1.08 × 10^10^ ON5 molecules, or significantly more NLS2-ON5 conjugate (*P* ≤ 0.0001; Figures 5E, S19). Assuming a cell number doubling rate of 24 h,^54,56^ we calculated an average of 291,000 NLS2-ON5 molecules per nucleus and 94,000 ON5 molecules per nucleus, or approximately 3-fold more NLS2-ON5 than ON5 (Table S5). Data for the cytoplasmic compartment are reported in Table S6. As controls, we also measured 18S gDNA^40^ and HPRT1 as described above (Figure S20). Normalized cellular uptake and nuclear localization data are listed in Figure S21.

**Figure 6:**
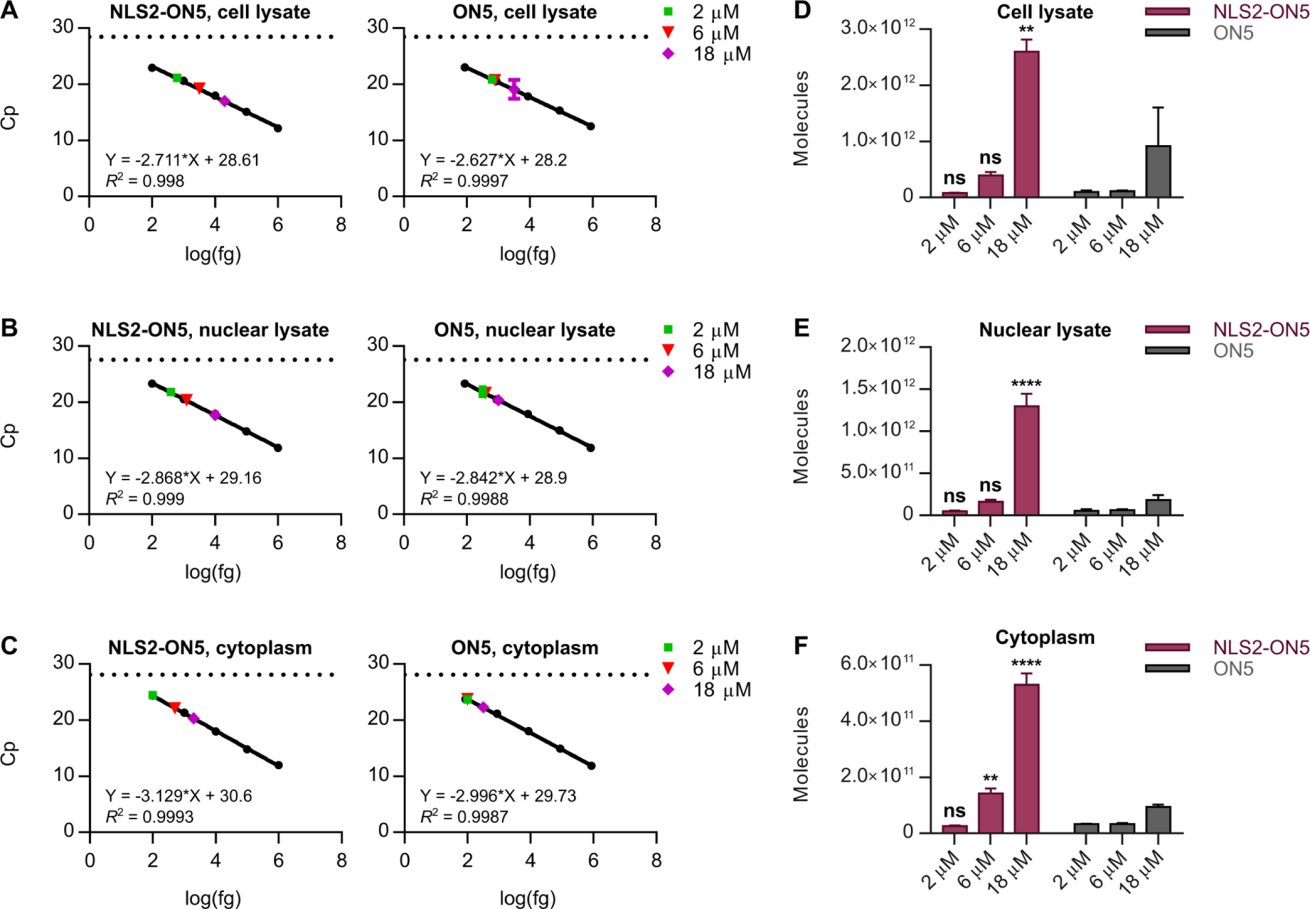
Cellular uptake and subcellular localization of a lead conjugate. (A–C) Linear fit CL-qPCR calibration curves for NLS2-ON5 and ON5 in HEL (A) cell lysate, (B) nuclear lysate, and (C) cytoplasm. The Cp values measured in each compartment following free uptake for 24 h are overlaid. Data are mean Cp values ± SD for three biological replicates (*n* = 3). (D–F) Oligonucleotide conjugates detected in HEL (D) cell lysate, (E) nuclear lysate, and (F) cytoplasm following free uptake for 24 h. Data are mean molecules ± SEM for three biological replicates (*n* = 3). Statistics are two-way ANOVA with Sidak’s multiple comparisons test against ON5, α = 0.05: ns, not significant; * *P* ≤ 0.05; ** *P* ≤ 0.01; *** *P* ≤ 0.001; and **** *P* ≤ 0.0001.

To confirm its improved nuclear localization properties, we treated HEL cells with NLS2-ON5 under free uptake conditions at 2, 6, or 18 μM. Again, we included ON5 in our treatments for reference. Interestingly, we noted that the cell culture medium became turbid upon the addition of 18 μM NLS2-ON5, but not ON5, to the cells; after 24 h, the medium remained turbid. At 24 h, the cells were lysed or fractionated as described above. We performed CL-qPCR on HEL cell lysate, nuclear lysate, and cytoplasm (Figures 6A–C, S22) and quantified the molecules in each compartment (Figures 6D–F, S23, Tables S7–S9). In cell lysate, we detected an average of 7.82 × 10^10^, 3.92 × 10^11^, and 2.60 × 10^12^ NLS2-ON5 molecules and 1.02 × 10^11^, 1.11 × 10^11^, and 9.18 × 10^11^ ON5 molecules following free uptake at 2, 6, and 18 μM, respectively (Table S7). Thus, up to 0.04% of all NLS2-ON5 molecules and 0.02% of all ON5 molecules applied to the cells were inside the cells after 24 h (Table S7). Assuming a cell number doubling rate of 24 h,^54,56^ we calculated an average of 680,000, 3,409,000, and 22,609,000 NLS2-ON5 molecules per cell and 887,000, 965,000, and 7,983,000 ON5 molecules per cell following free uptake at the 2, 6, and 18 μM concentrations, respectively (Table S7). In nuclear lysate, we detected an average of 4.94 × 10^10^, 1.61 × 10^11^, and 1.30 × 10^12^ NLS2-ON5 molecules and 5.42 × 10^10^, 6.13 × 10^10^, and 1.84 × 10^11^ ON5 molecules following free uptake at 2, 6, and 18 μM, respectively (Table S8). Thus, up to 63% of all NLS2-ON5 molecules and 55% of all ON5 molecules inside the cells could be accounted for in the nuclear compartment after 24 h (Table S8). Assuming a doubling rate of 24 h,^54,56^ we calculated an average of 430,000, 1,400,000, and 11,304,000 NLS2-ON5 molecules per nucleus and 471,000, 533,000, and 1,600,000 ON5 molecules per nucleus following free uptake at the 2, 6, and 18 μM concentrations, respectively (Table S8). Data for the cytoplasmic compartment are reported in Table S9.

Based on the change in appearance of the cell culture medium upon the addition of 18 μM NLS2-ON5, we speculate that NLS2-ON5 might form aggregates or nanoparticles that facilitate its entry into cells. This phenomenon has been previously reported for cationic peptide–oligonucleotide conjugates^59,60^ and is mediated by scavenger receptors.^60,61^ As controls, we measured 18S gDNA^40^ and HPRT1 as described above (Figure S24). Intriguingly, we observed increased 18S gDNA signals in the cell and nuclear lysate for NLS2-ON5 at 18 μM (Figure S24). At this time, this result remains unexplained, although we speculate that it may be related to the possible formation of aggregates or nanoparticles by NLS2-ON5, as noted above. Normalized cellular uptake and nuclear localization data are listed in Figure S25.

### Generation of FECH Minigenes and FECH-3-C-5 Stable Cell Lines

To investigate the uptake–activity relationship of our peptide–oligonucleotide conjugates, a cellular system modeling the SNP-mediated FECH splicing defect was required. Prior attempts to generate a FECH splicing deficient K562 cell line via gene editing had yielded a FECH knockout line with limited viability.^62^ And while a minigene system for the EPP-associated splicing defect has been described before,^63^ this system requires transient transfection of the plasmid, which would likely interfere with membrane integrity and alter the uptake and localization characteristics of our compounds. We therefore generated a new FECH splicing defect minigene in a vector capable of stable integration in order to circumvent the need for transient transfection. To this end, genomic regions of the wild-type FECH gene spanning exons 3–5 were cloned into the pCI-neo vector (Figure S26A). The splicing defect minigene harboring the c.315-48C SNP was successfully generated by site-directed mutagenesis (Figure S26A). The functionality of the FECH-C minigene plasmid was confirmed by transient transfection into 293T cells, followed by a splice-switching assay (data not shown).

Using the newly generated FECH-C plasmid, two cell lines modeling the EPP-associated splicing defect were successfully derived from human embryonic kidney 293 cells^64^ and K562 cells (Figure S26B,C). K562 cells are lymphoblast cells isolated from the bone marrow of a chronic myelogenous leukemia patient.^65^ Our K562 FECH-3-C-5 cells produce high amounts of the 63-nt longer aberrant FECH transcript (Figure S26D) and show no attenuation in growth under normal culturing conditions. Our 293 FECH-3-C-5 cells produce roughly equal amounts of the correct and aberrant FECH transcripts (Figure S26D). It is worth noting that we do not expect the ratios of the two transcripts to reflect the proportions seen in EPP patients. With the construct under the control of the constitutive CMV promoter, the aberrant product will be produced at hyperphysiological rates and could overwhelm the nonsense-mediated decay machinery. In fact, K562 FECH-3-C-5 cells produce 4-fold more correct transcript and approximately 400-fold more aberrant transcript than the parental K562 cells (data not shown). In addition to the expected correct and aberrant transcripts, the 293 FECH-3-C-5 cells also show higher molecular weight products with a low abundance. These may be caused by integration events in which the plasmid was linearized within the minigene region. These artifacts, however, do not interfere with the quantification of splice-switching activity and would likely be removed by clonal selection of the batch culture. In 293 FECH-3-C-5 cells, we confirmed that our SSOs targeting the EPP-associated c.315-48C polymorphism have splice-switching activity, while the negative control ON7 was inactive (Figure S26E). The effects of gymnotic treatment with ON5 and ON7 on K562 FECH-3-C-5 cell viability were determined during preliminary dose-finding (data not shown) via trypan blue staining in a Countess 3 Automated Cell Counter (Invitrogen). No reduction in viability was observed for treatments below 6 μM after 48 h. While a reduction in viability to 55% was observed at 18 μM after 48 h, no significant difference in viability was detected between ON5 and ON7 at any concentration tested, indicating the absence of sequence-specific toxicity.

Attempts to generate a FECH splicing defect model cell line in HEL cells were not successful owing to poor viability following plasmid transfection (data not shown).

### *In Vitro* Splice-Switching Activities of the Conjugates

**Figure 7:**
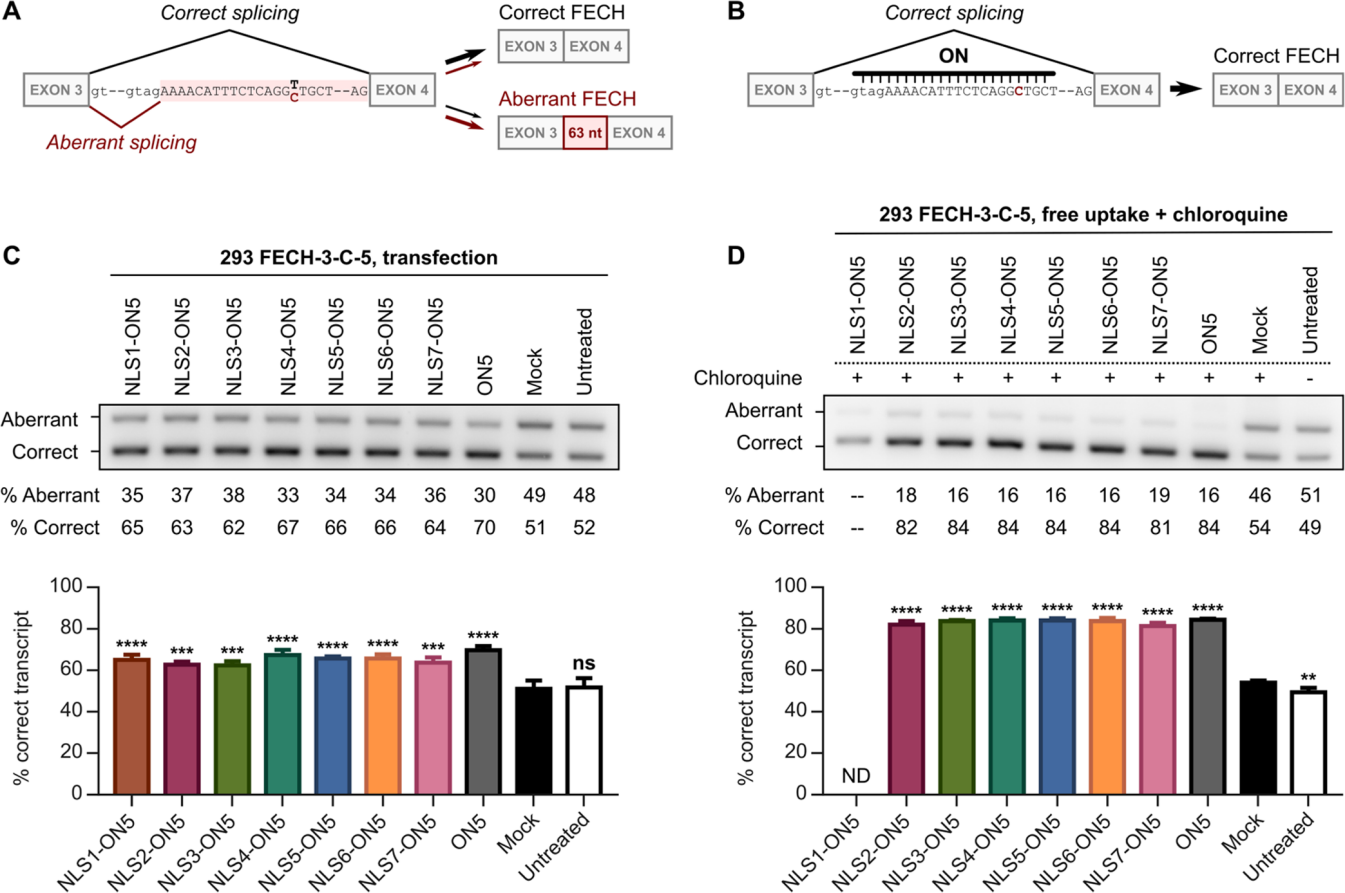
*In vitro* splice-switching activities of conjugates. (A) The production of correct and aberrant FECH transcripts from c.315–48T and c.315-48C FECH pre-mRNA. (B) The principle of splice correction by an SSO. (C) Splice-switching activities of conjugates and parent SSO following transfection in 293 FECH-3-C-5 cells. Transfections were performed using Lipofectamine 2000 and 10 nM conjugate or oligonucleotide for 48 h. Mock was treated with Lipofectamine alone. (D) Splice-switching activities of conjugates and parent SSO following free uptake in 293 FECH-3-C-5 cells and treatment with chloroquine. Treatments were performed using 500 nM conjugate or oligonucleotide for 24 h, followed by 60 μM chloroquine for 24 h. Mock was treated with chloroquine alone. ND, not determined. The semiquantitation of bands for NLS1-ON5 was not possible owing to a limited amount of RNA recovered from the cells treated with this compound. In (C) and (D), a representative gel image is shown at the top, and semiquantitation is plotted below. Data are mean percent (%) correct transcript of total transcript (i.e., aberrant + correct) ± SD for three biological replicates (*n* = 3). Statistics are one-way ANOVA with Dunnett’s multiple comparisons test against mock, α = 0.05: ns, not significant; * *P* ≤ 0.05; ** *P* ≤ 0.01; *** *P* ≤ 0.001; and **** *P* ≤ 0.0001.

Next, we measured the splice-switching activity of the lead conjugate *in vitro*. The production of correct and aberrant transcripts from FECH pre-mRNA is illustrated in Figure 7A, and the principle of splice correction by an SSO is illustrated in Figure 7B. We started by treating K562 FECH-3-C-5 cells with NLS2-ON5 under free uptake conditions at 6 μM. As a positive control for splice correction,^36^ we included ON5 in our treatments. At 48 h, the cells were lysed, and RNA was extracted, reverse transcribed, and amplified by PCR. The PCR products were then separated on an agarose gel and visualized (Figure S27A). As expected, treatment with ON5 shifted the ratio toward the correct transcript (Figure S27A). However, treatment with NLS2-ON5 did not (Figure S27A). We repeated the assay in 293 FECH-3-C-5 cells and obtained the same result (Figure S27B). To determine whether any conjugate in our library has activity under free uptake conditions, we treated K562 FECH-3-C-5 cells with the conjugates under free uptake conditions at 6 μM for 48 h, when the cells were processed as described above. Surprisingly, under these conditions, all conjugates were inactive (Figure S28).

Because peptide–oligonucleotide conjugates and their parent oligonucleotides can have distinct pathways of cellular uptake and intracellular trafficking,^66^ our next step was to control for uptake and trafficking by transfecting the conjugates into 293 FECH-3-C-5 cells. For our experiments, we used the cationic lipid-based transfection reagent Lipofectamine 2000, which achieves delivery of exogenous DNA and RNA into cells and largely avoids the entrapment and metabolic degradation of nucleic acid payloads within acidic/digestive lysosomal compartments.^67^ Indeed, the endosomal escape and nuclear accumulation of oligonucleotides delivered into cells by Lipofectamine 2000 occurs within 5 min of treatment.^68^ At 48 h post-transfection, the cells were processed as described above. A representative gel image is shown in Figure 7C; all gel images are provided in Figures S29–S31. Under these conditions, all conjugates were active, yielding similar or slightly less amounts of the correct transcript relative to the parent SSO (Figures 7C, S29–S31).

Having established that the conjugates are capable of splice correction provided uptake and trafficking are controlled for, we suspected that the conjugates are trapped in endosomes following free uptake. To test this hypothesis, we started by treating 293 FECH-3-C-5 cells with NLS2-ON5 under free uptake conditions at 500 nM, 2 μM, or 6 μM. After 24 h, the cells were washed with PBS and treated with complete medium containing 60 μM chloroquine, an endolytic small molecule.^45^ As a positive control for splice correction, we treated cells with ON5 at 6 μM for 24 h followed by complete medium for 24 h (i.e., no chloroquine). After 24 h of exposure to chloroquine or the medium alone, the cells were washed with PBS and processed as described above. In the cells treated with lead conjugate and chloroquine, we observed near complete splice correction at all concentrations tested (Figure S32), indicating that the major barrier to the activity of NLS2-ON5 is endosomal entrapment. We then repeated the assay for all of the conjugates in our library as well as ON5 at the 500 nM concentration. A representative gel image is shown in Figure 7D; all gel images are provided in Figures S33–S35. Under these conditions, all conjugates were active and yielded similar amounts of the correct transcript as the parent SSO (Figure 7D), indicating that the major barrier to the activity of the peptide–oligonucleotide conjugates is endosomal entrapment. Notably, under these conditions, the parent SSO ON5 also displayed a level of splice-switching activity not previously observed under free uptake conditions, which is consistent with some trapping of this molecule in the endolysosomal pathway following free uptake (Figure 7D).

## Conclusions

In this study, we aimed to identify peptides that improve the nuclear localization and biological activity of splice-switching ASOs. As model sequences, we selected SSOs previously developed to treat EPP.^35,36^ To quantify the oligonucleotides in cells and subcellular compartments following free uptake, we employed CL-qPCR,^36,40,41^ a technique we established as superior to Splint ligation-qPCR^49^ at detecting an ASO with MOE PS chemistry (Figure 2C,D). Using CL-qPCR, we detected approximately 50,000 to 200,000 ASO molecules per nucleus following free uptake at low micromolar concentrations for 24 h (Table S2), which is consistent with previous reports.^33,34^ Then, we covalently conjugated different NLS peptides to a lead MOE PS oligonucleotide using thiol–maleimide chemistry.^44,47^ Using CL-qPCR, we identified one conjugate with better nuclear accumulation relative to the parent SSO (Figures 5E, 6E). However, in contrast to the parent, unconjugated SSO, the conjugates were inactive at splice correction under free uptake conditions *in vitro*. We acknowledge an apparent tension between the enrichment of our lead peptide–oligonucleotide conjugate in the nuclear compartment and its inactivity following free uptake, which we attribute to endosomal entrapment. We reconcile these observations by speculating that our nuclear fractions are contaminated by the perinucleus, a membraneless organelle that extends a few micrometers from the nucleus.^69^ The perinucleus contains 15 to 18% of the total proteins of the mammalian cell and is a site for the efficient maturation of endosomes.^69,70^ Previous reports have shown that ASOs^32^ and ASO-loaded endosomes^71^ localize to the perinuclear region. Future studies may elaborate on our findings by employing a higher-resolution subcellular fractionation protocol to dissect the cell into nucleus, perinucleus, and cytosol.^72^

In our UV melting studies, conjugates paired with an RNA target displayed *T*_m_s that were similar to the *T*_m_ of the parent SSO paired with the same RNA target (Figures 1C, 4D). However, we cannot exclude the possibility that in the context of a cell, electrostatic interactions between the net positively charged peptide and negatively charged oligonucleotide backbone reduce the accessibility of the oligonucleotide to its RNA target and thus hamper the splice-switching activity of the conjugates. Nevertheless, we were able to confer splice-switching activity on the conjugates by transfecting them into cells (Figure 7C). Additionally, treating the cells into which the conjugates had been freely taken up with chloroquine, an endolytic small molecule,^45^ resulted in robust splice-switching activity (Figure 7D). Our results identify the major barrier to the activity of the peptide–oligonucleotide conjugates as endosomal entrapment and may help explain our earlier observation that a conjugated peptide enhances the accumulation, but not the activity, of the same MOE PS SSO in the bone marrow.^36^ In the oligonucleotide therapeutics field, endosomal escape is seen as a long-standing, rate-limiting, and recalcitrant problem.^46^ Put simply in the words of Dowdy et al., endosomal escape is the delivery problem.^73^ ASOs are too large, hydrophilic, and/or charged to passively diffuse across the cellular membrane; instead, they are taken up by endocytosis.^24^ However, endosomes also comprise a lipid bilayer, and they are estimated to retain 99% of RNA therapeutics, with less than 1% entering the cytoplasm.^46^ Today, the mechanism by which oligonucleotides escape the endosome remains unclear. A favored hypothesis is that escape from endocytic compartments occurs at sites of membrane fission and fusion through breaches in the lipid bilayer.^74^ Undoubtedly, a more detailed understanding of the dynamic intracellular trafficking pathway is important to overcoming this key constraint. Although endolytic agents typified by chloroquine achieve endosomal rupture, such agents are active in target and nontarget cells alike, resulting in a level of cytotoxicity unacceptable for applications *in vivo*.^46^ Moving forward, the development of new strategies to enhance endosomal escape, for example, through the use of small molecules that disrupt endolysosomal trafficking,^75^ will prove critical to unlocking the potential of therapeutic oligonucleotides.

Overall, our findings are of interest to many groups working to identify ligands that can enhance the activity of therapeutic oligonucleotides *in vivo*. As a class, peptides are attractive candidates because of their receptor-binding properties. Additionally, the relatively straightforward solid-phase synthesis of peptide–oligonucleotide conjugates is an attractive feature in comparison to that of carbohydrates such as *N*-acetylgalactosamine^76^ ligands or mannose^77^ units. However, to date, few studies have compared the antisense activity of peptide–oligonucleotide conjugates with the parent oligonucleotide itself.^78^ The work presented here helps to fill this gap in knowledge and identifies a potential bottleneck in the progression of peptide–oligonucleotide conjugates from the bench into the clinic.

## Supporting Information Available

LC–MS chromatograms for oligonucleotides; melting data for oligonucleotides; LC–MS chromatograms for chemical ligators; linear fit CL-qPCR calibration curves for oligonucleotides in water using BPS ligator (C); linear fit CL-qPCR calibration curves for oligonucleotides in water using BPS ligator (T); comparison of linear fit CL-qPCR calibration curves for oligonucleotides in water using the different BPS ligators; linear fit CL-qPCR calibration curves for oligonucleotides in HEL cell lysate; linear fit CL-qPCR calibration curves for oligonucleotides in HEL nuclear lysate; linear fit CL-qPCR calibration curves for oligonucleotides in HEL cytoplasm; oligonucleotides detected following free uptake for 24 h; oligonucleotides detected in HEL cell lysate following free uptake for 24 h; oligonucleotides detected in HEL nuclear lysate following free uptake for 24 h; oligonucleotides detected in HEL cytoplasm following free uptake for 24 h; quantification of 18S gDNA and HPRT1 following free uptake of the oligonucleotides in HEL cells for 24 h; femtograms (fg) oligonucleotide per unit 18S gDNA detected following free uptake in HEL cells for 24 h; LC–MS chromatograms for conjugates; melting data for conjugates; linear fit CL-qPCR calibration curves for conjugates in water; linear fit CL-qPCR calibration curves for conjugates in HEL cell lysate; linear fit CL-qPCR calibration curves for conjugates in HEL nuclear lysate; linear fit CL-qPCR calibration curves for conjugates in HEL cytoplasm; oligonucleotide conjugates detected following free uptake at 2 μM for 24 h; oligonucleotide conjugates detected in HEL cell lysate following free uptake at 2 μM for 24 h; oligonucleotide conjugates detected in HEL nuclear lysate following free uptake at 2 μM for 24 h; oligonucleotide conjugates detected in HEL cytoplasm following free uptake at 2 μM for 24 h; quantification of 18S gDNA and HPRT1 following free uptake of the conjugates in HEL cells at 2 μM for 24 h; femtograms (fg) conjugate per unit 18S gDNA detected following free uptake of the conjugates in HEL cells at 2 μM for 24 h; linear fit CL-qPCR calibration curves for the lead conjugate; lead oligonucleotide conjugate detected following free uptake of the conjugate in HEL cells for 24 h; lead oligonucleotide conjugate detected in HEL cell lysate following free uptake of the conjugate in HEL cells for 24 h; lead oligonucleotide conjugate detected in HEL nuclear lysate following free uptake of the conjugate in HEL cells for 24 h; lead oligonucleotide conjugate detected in HEL cytoplasm following free uptake of the conjugate in HEL cells for 24 h; quantification of 18S gDNA and HPRT1 following free uptake of the lead conjugate in HEL cells for 24 h; femtograms (fg) lead conjugate per unit 18S gDNA detected following free uptake of the conjugate in HEL cells for 24 h; generation of FECH minigenes and FECH-3-C-5 stable cell lines; splice-switching activities of the lead conjugate and parent SSO following free uptake; splice-switching activities of all conjugates and parent SSO following free uptake; splice-switching activities of all conjugates and parent SSO following transfection, replicate 1; splice-switching activities of all conjugates and parent SSO following transfection, replicate 2; splice-switching activities of all conjugates and parent SSO following transfection, replicate 3; splice-switching activity of the lead conjugate following free uptake and treatment with chloroquine; splice-switching activities of all conjugates and parent SSO following free uptake and treatment with chloroquine, replicate 1; splice-switching activities of all conjugates and parent SSO following free uptake and treatment with chloroquine, replicate 2; splice-switching activities of all conjugates and parent SSO following free uptake and treatment with chloroquine, replicate 3 (PDF)
