# Supplementary material for: Peptide Conjugates of a 2′-O-Methoxyethyl Phosphorothioate Splice-Switching Oligonucleotide Show Increased Entrapment in Endosomes
Source: ACS Omega. 2023 Oct 17;8(43):40463–81. doi: 10.1021/acsomega.3c05144 (PMC10620785; doi:10.1021/acsomega.3c05144)
Supplement: Supplementary file 1 — ao3c05144_si_001.pdf [file ao3c05144_si_001.pdf]

Supporting Information for

Peptide conjugates of a 2'-*O*-methoxyethyl phosphorothioate splice-switching oligonucleotide show increased entrapment in endosomes

Alyssa C. Hill[1]*, J. Philipp Becker[1], Daria Slominski[1], François Halloy[1], Christoffer Søndergaard[2], Jacob Ravn[2], and Jonathan Hall[1]*

[1] Institute of Pharmaceutical Sciences, Department of Chemistry and Applied Biosciences, Eidgenössische Technische Hochschule Zürich (ETH Zürich), Switzerland

[2] Roche Innovation Center Copenhagen (RICC), Hørsholm, Denmark

* Co-corresponding authors

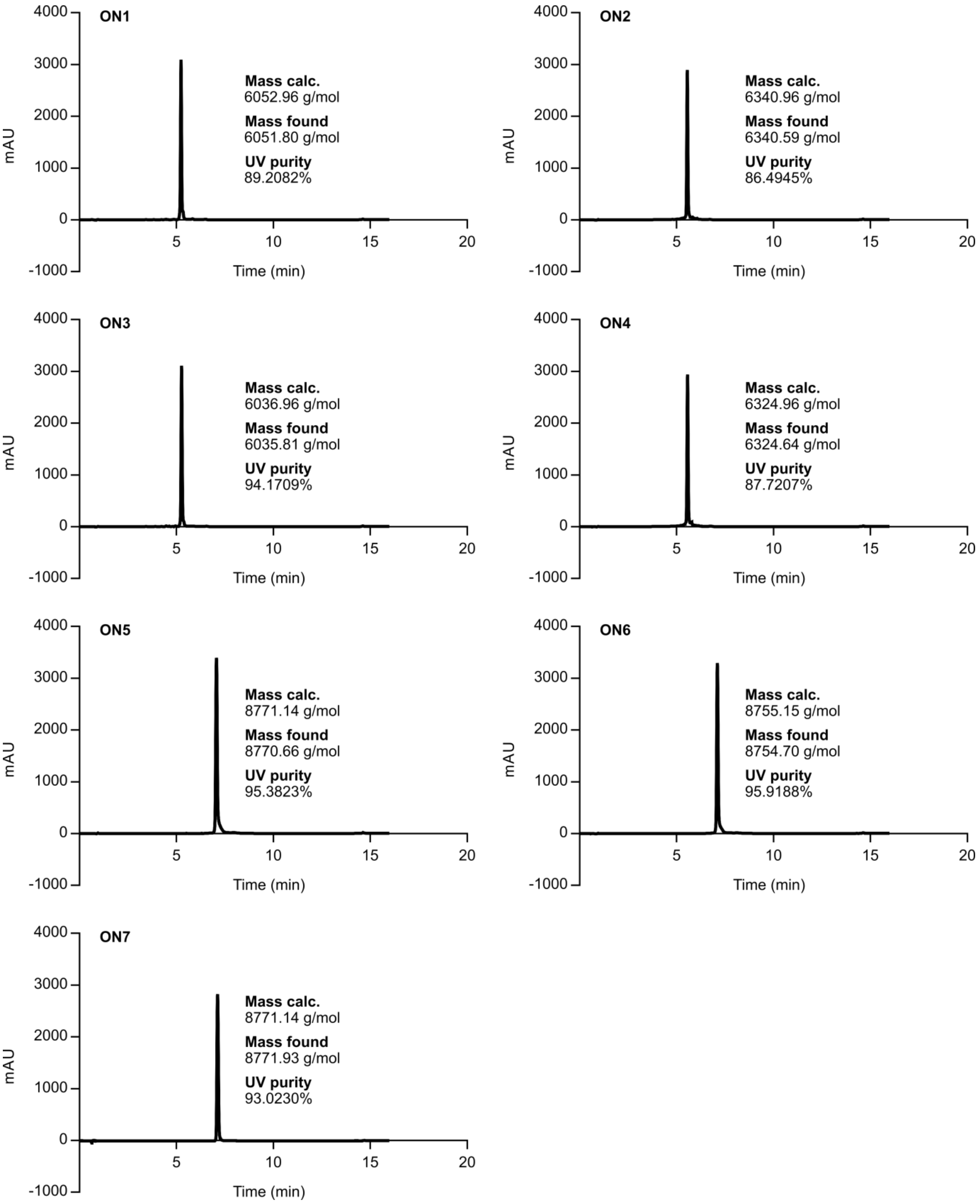

Figure S1. LC–MS chromatograms for oligonucleotides. ON, oligonucleotide. Masses were calculated using the Oligowizard Nucleic Acid Calculator, an online tool available at http://oligowizard.com/. UV purity is expressed as percent area under the peak.

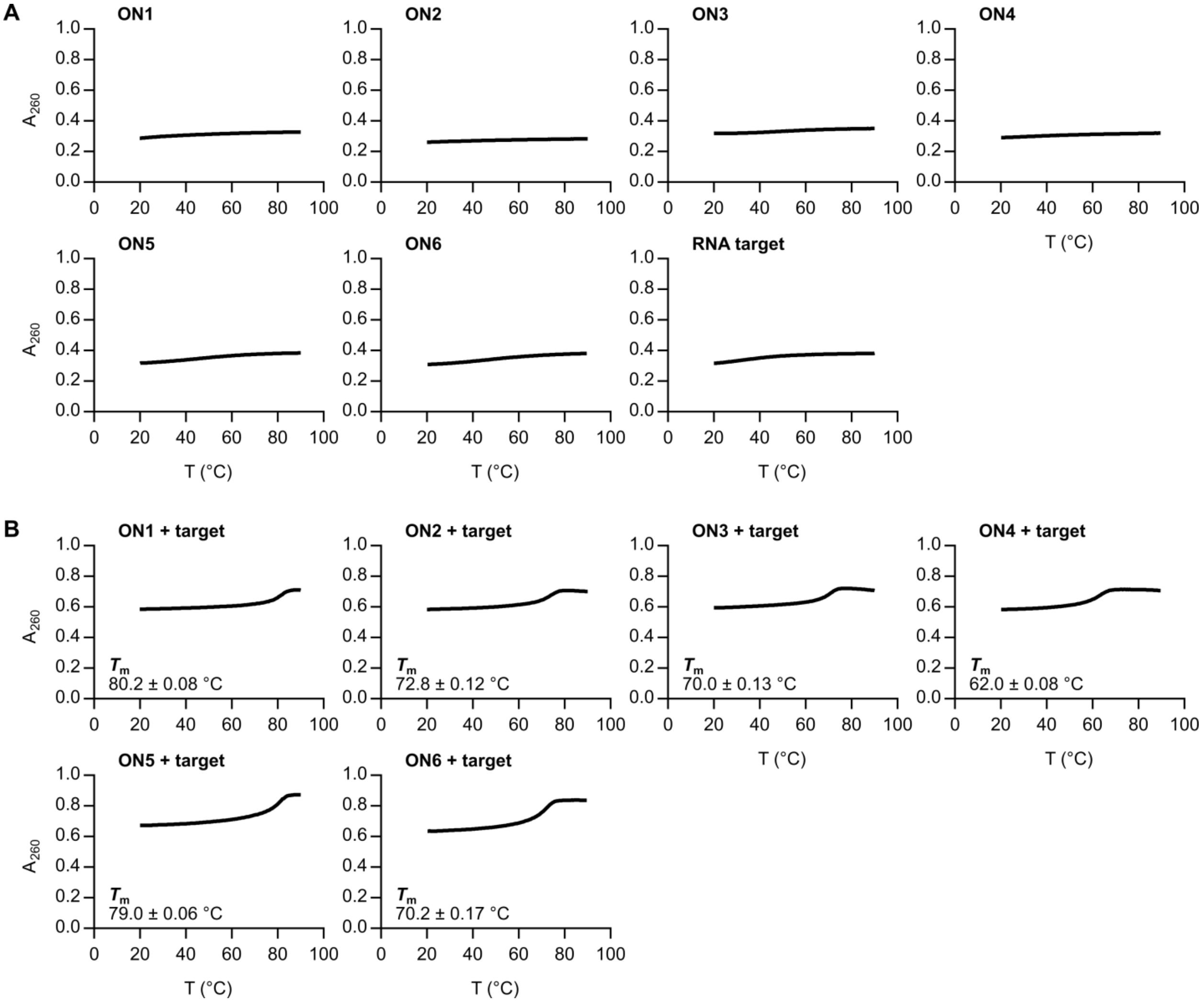

Figure S2. Melting data for oligonucleotides. (A) Representative dataset for single strands in 100 mM NaCl, 10 mM phosphate, 0.1 mM Na_2EDTA, pH 7.0. (B) Representative dataset for the oligonucleotides paired with a 22-nt RNA representing the c.315-48C FECH pre-mRNA in the same buffer. In (B), T_ms were determined from first derivative analyses of nonlinear fit melting curves. Data are mean ± SD for three technical replicates. T_m, melting temperature.

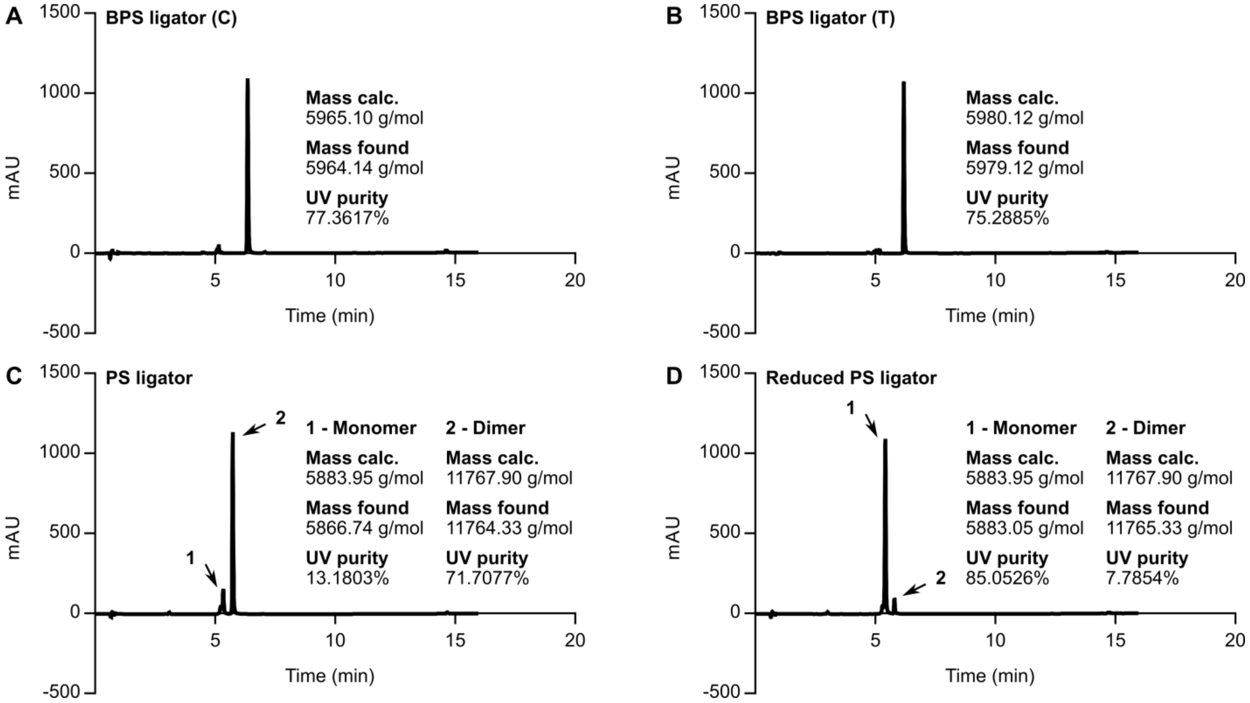

Figure S3. LC-MS chromatograms for chemical ligators. (A) LC-MS chromatogram for the BPS ligator (C). (B) LC-MS chromatogram for the BPS ligator (T). (C) LC-MS chromatogram for the PS ligator. The PS ligator bears a 3' terminal PS group that enables dimerization. To prevent spontaneous sulfur-oxygen exchange, the PS ligator was stored as a dimer. Immediately prior to use, an aliquot of the PS ligator was reduced by adding Tris(carboxyethyl)phosphine hydrochloride (TCEP HCl; Fluorochem M02624) or Bond-Breaker TCEP Solution, Neutral pH (Thermo Scientific 77720) to a final concentration of 50 µM. (D) LC-MS chromatogram for the reduced PS ligator. In (A) through (D), masses were calculated using the Oligowizard Nucleic Acid Calculator, an online tool available at http://oligowizard.com/. UV purity is expressed as percent area under the peak. BPS, biphenylsulfonyl; PS, phosphorothioate.

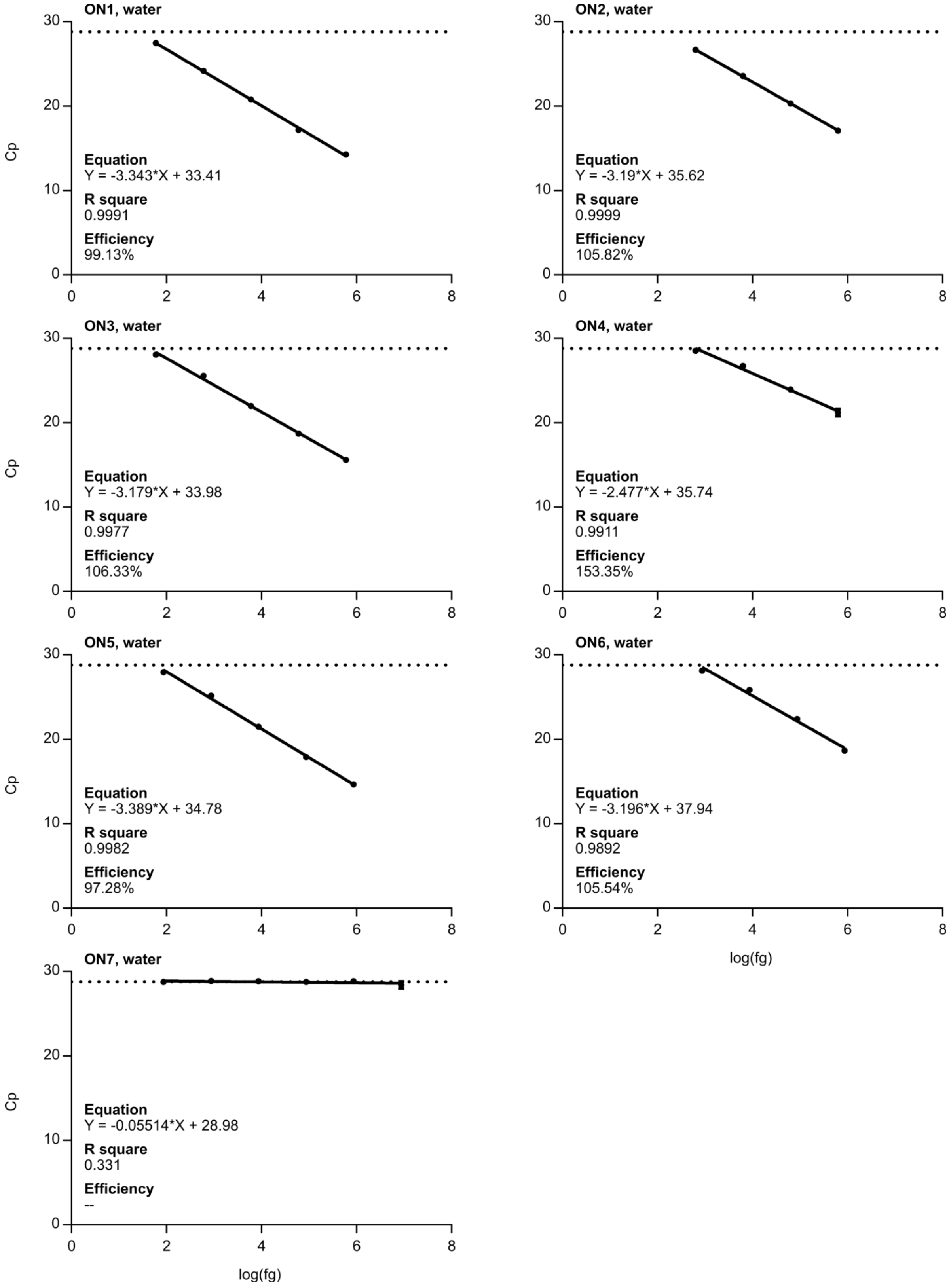

Figure S4. Linear fit CL-qPCR calibration curves for oligonucleotides in water using BPS ligator (C). Data are mean Cp values ± SD for three technical replicates. Efficiencies were calculated using the ThermoFisher Scientific qPCR Efficiency Calculator.

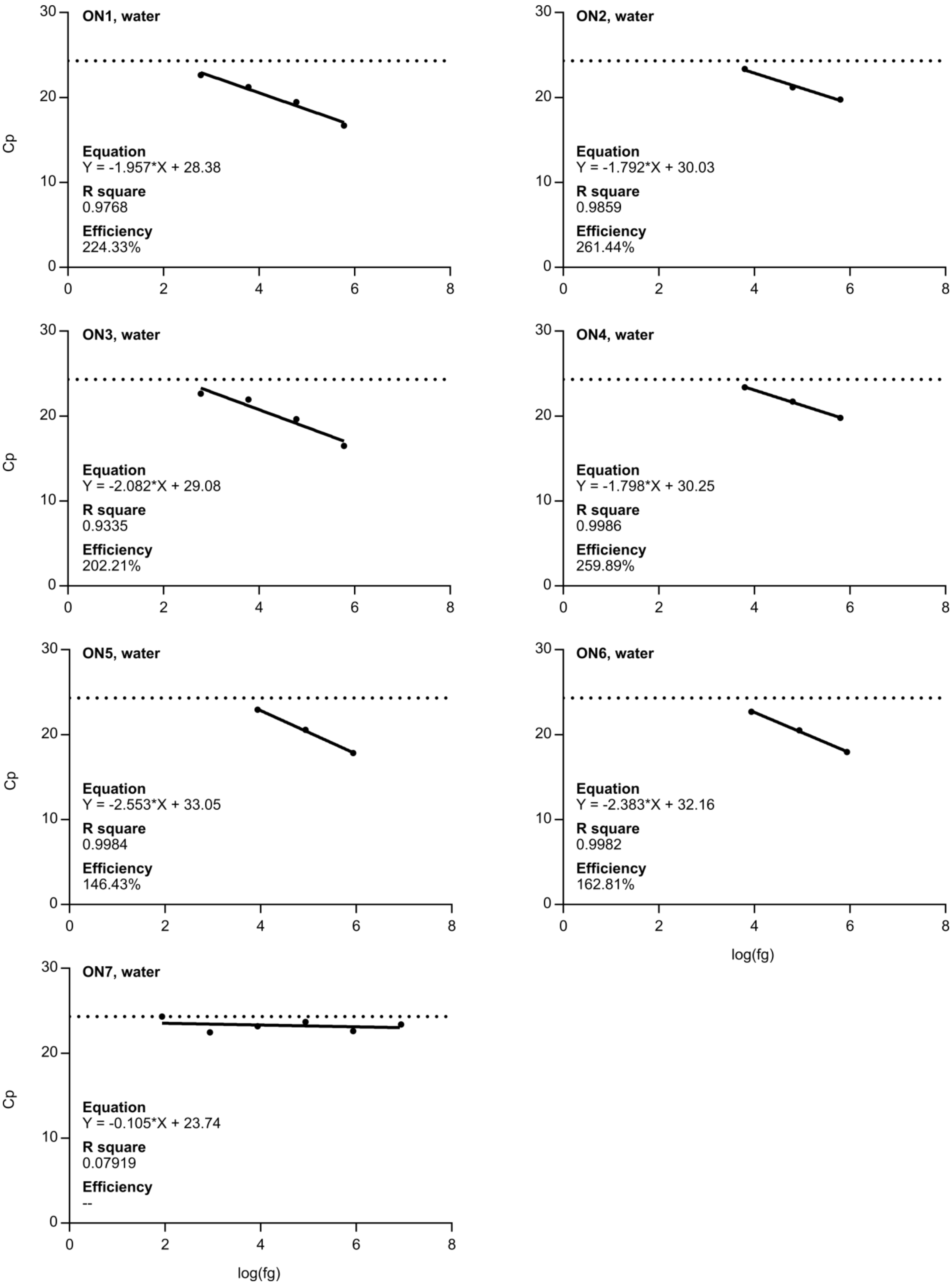

Figure S5. Linear fit CL-qPCR calibration curves for oligonucleotides in water using BPS ligator (T). Data are mean Cp values ± SD for three technical replicates. Efficiencies were calculated using the ThermoFisher Scientific qPCR Efficiency Calculator.

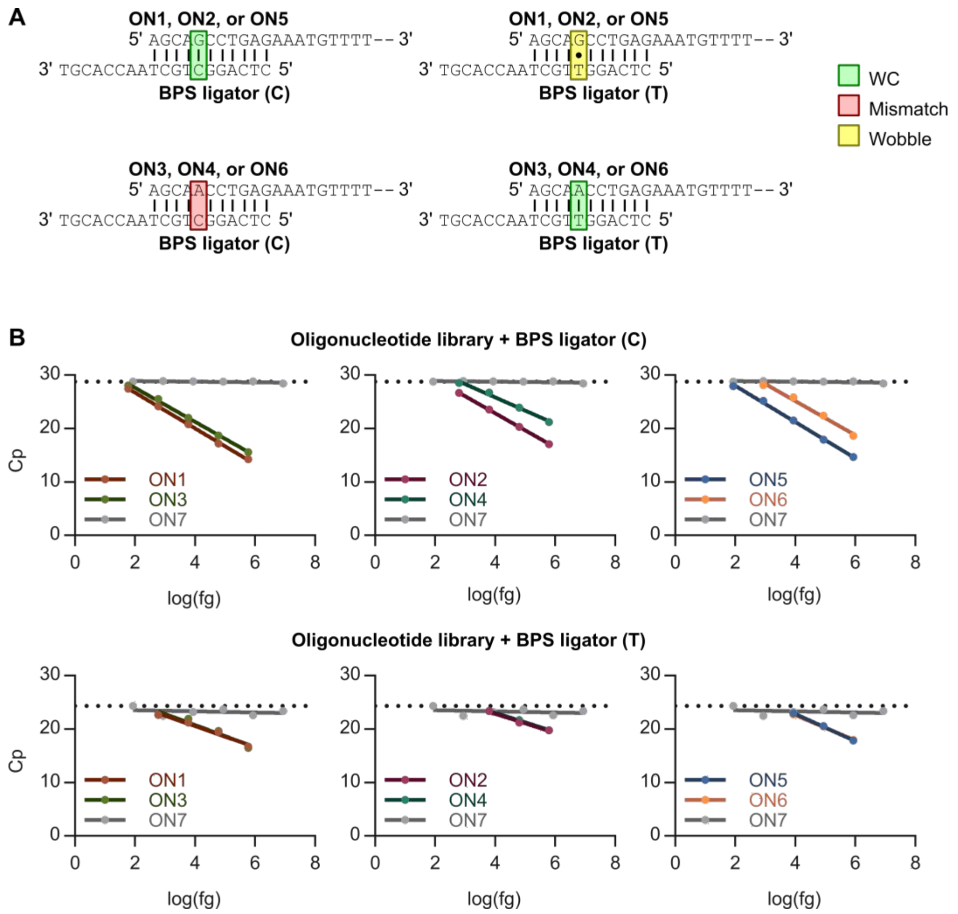

Figure S6. Comparison of linear fit CL-qPCR calibration curves for oligonucleotides in water using the different BPS ligators. (A) Base pairing interactions between the oligonucleotides and the different BPS ligators. (B) Linear fit CL-qPCR calibration curves for oligonucleotides in water using the different BPS ligators. In (A), lines are Watson-Crick base pairs and dots are wobble base pairs. WC, Watson-Crick. In (B), data are mean Cp values ± SD for three technical replicates and the same as those presented in Figures S4 and S5.

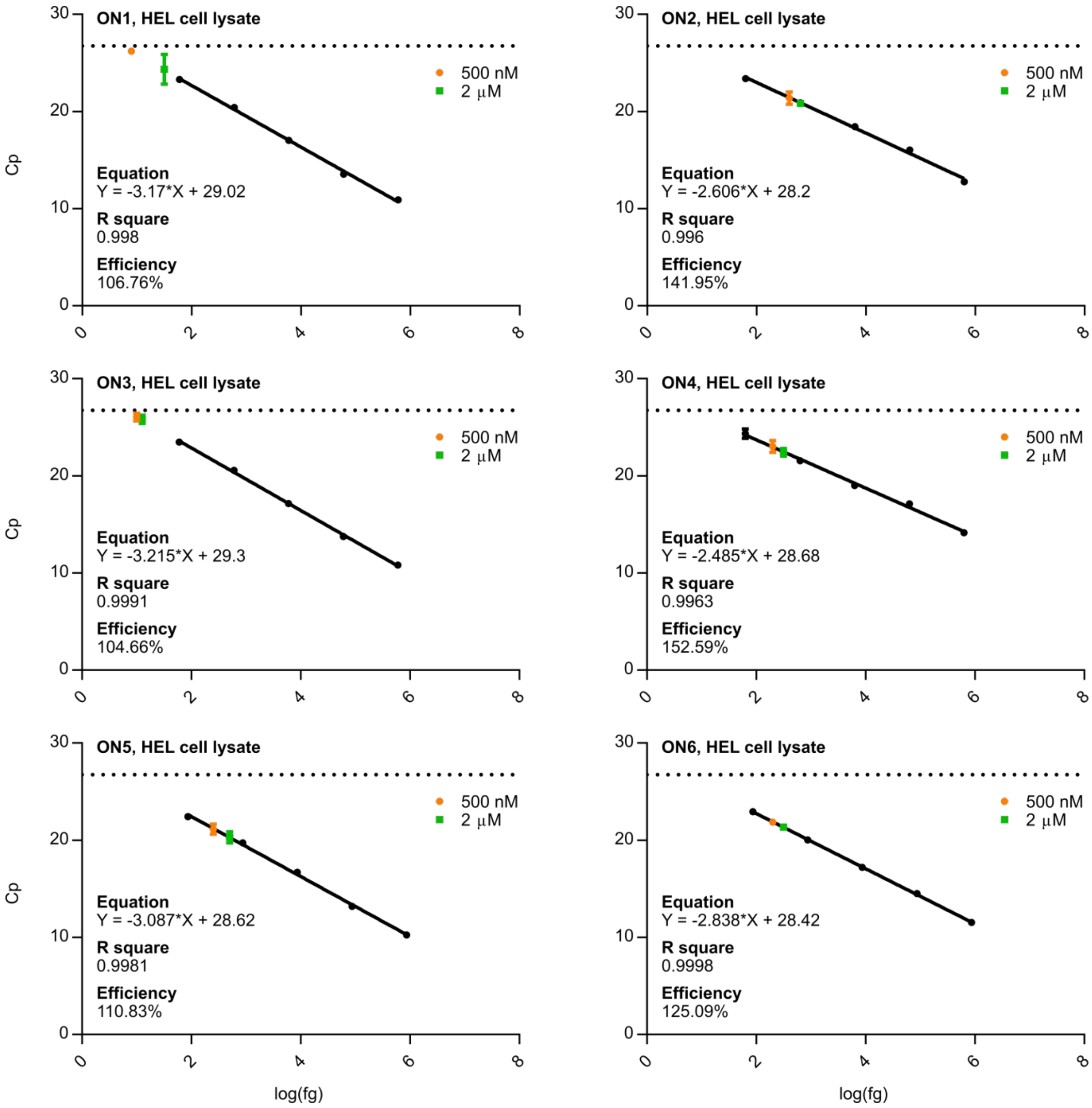

Figure S7. Linear fit CL-qPCR calibration curves for oligonucleotides in HEL cell lysate. The Cp values measured in HEL cell lysates following free uptake for 24 h are overlaid. Data are mean Cp values ± SD for three biological replicates (n=3). Efficiencies were calculated using the ThermoFisher Scientific qPCR Efficiency Calculator.

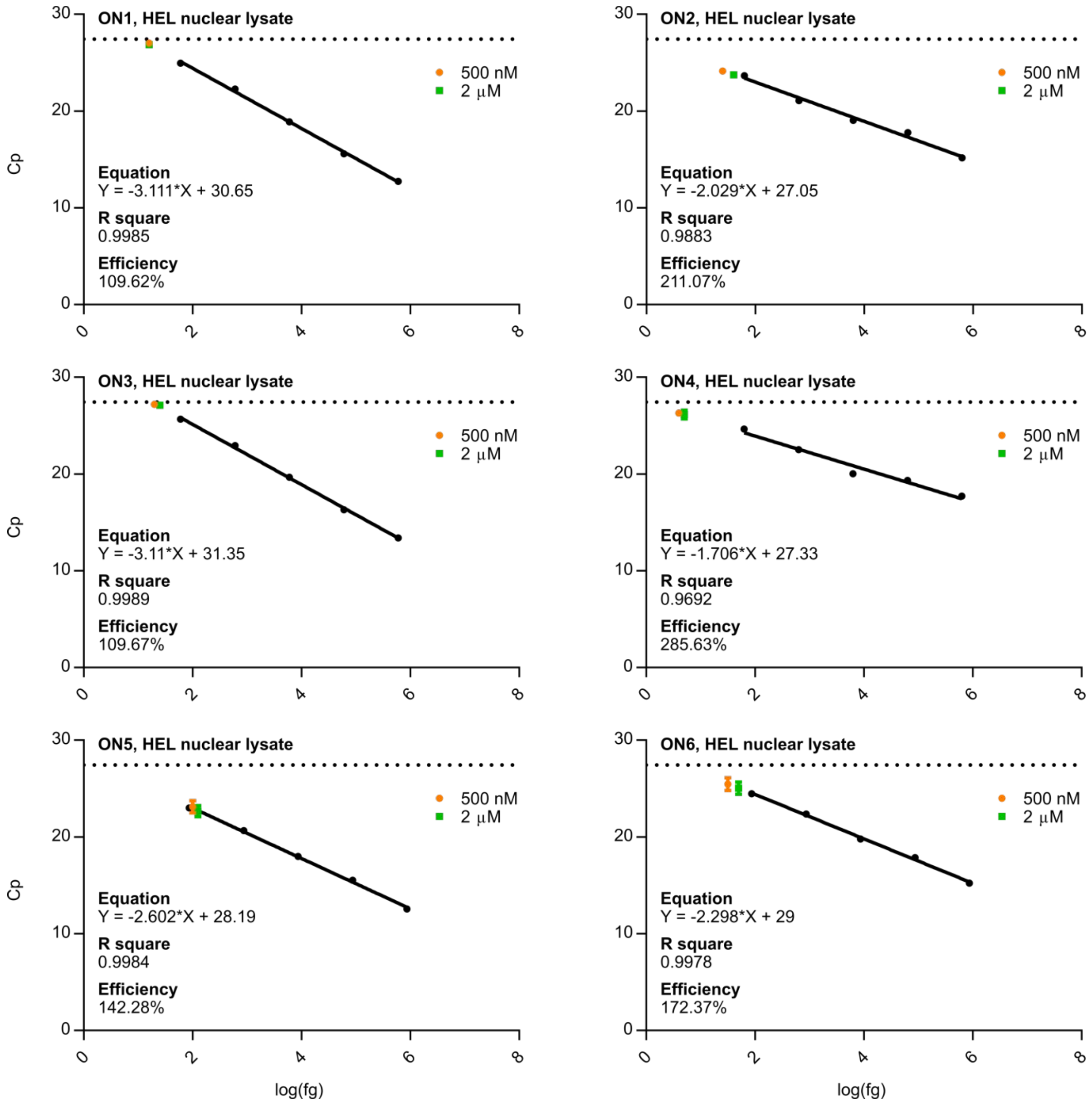

Figure S8. Linear fit CL-qPCR calibration curves for oligonucleotides in HEL nuclear lysate. The Cp values measured in HEL nuclear lysates following free uptake for 24 h are overlaid. Data are mean Cp values ± SD for three biological replicates (n=3). Efficiencies were calculated using the ThermoFisher Scientific qPCR Efficiency Calculator.

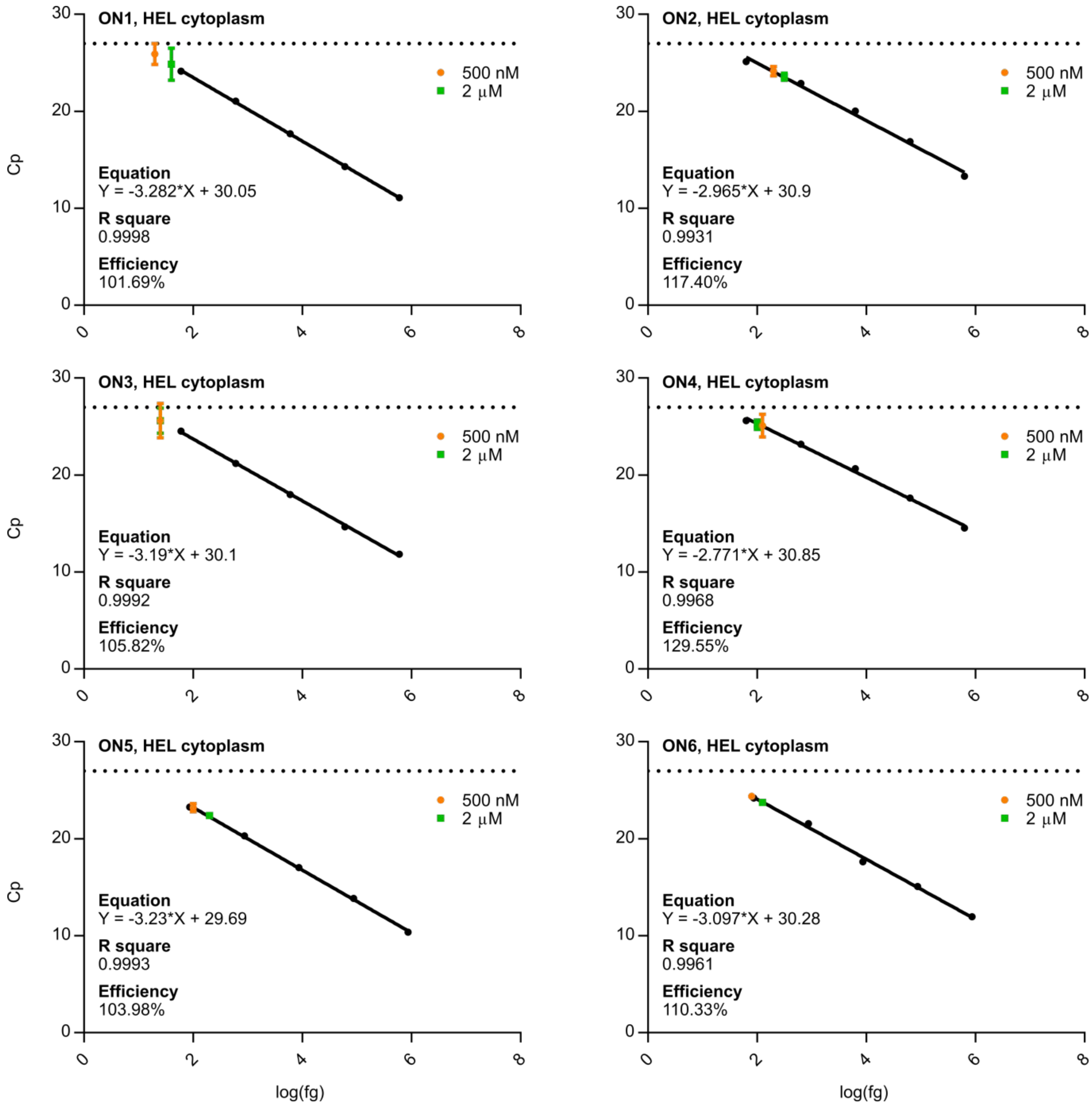

Figure S9. Linear fit CL-qPCR calibration curves for oligonucleotides in HEL cytoplasm. The Cp values measured in HEL cytoplasm following free uptake for 24 h are overlaid. Data are mean Cp values ± SD for three biological replicates (n=3). Efficiencies were calculated using the ThermoFisher Scientific qPCR Efficiency Calculator.

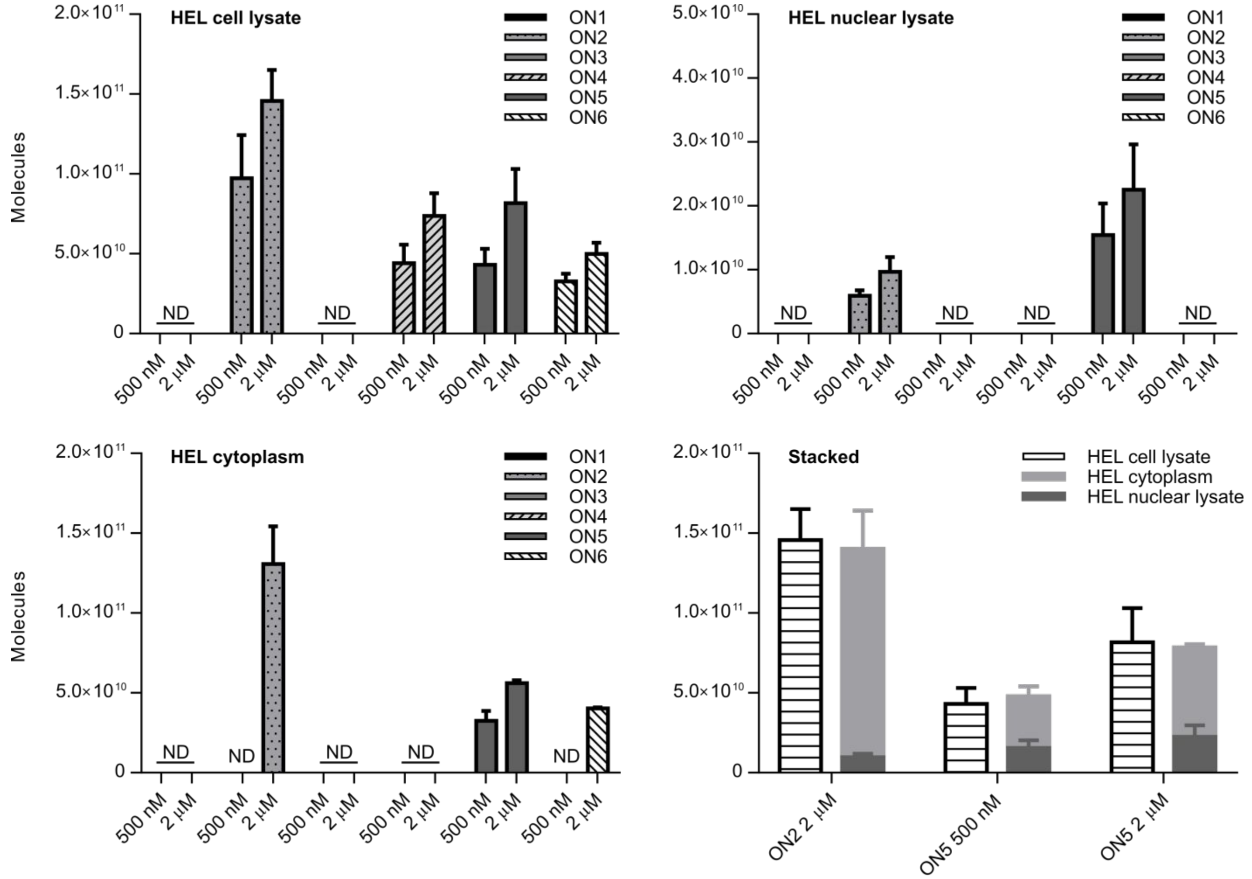

Figure S10. Oligonucleotides detected following free uptake for 24 h. Oligonucleotides detected in HEL cell lysate (*top left*), nuclear lysate (*top right*), and cytoplasm (*bottom left*). A stacked view (*bottom right*) shows the oligonucleotides detected in HEL cell lysate are approximately equal to the sum of the oligonucleotides detected in HEL nuclear lysate and the oligonucleotides detected in HEL cytoplasm. Data are mean molecules ± SEM for three biological replicates (n=3). ND, not detected.

Table S1. Oligonucleotides detected in HEL cell lysate following free uptake for 24 h. Data are mean molecules ± SEM for three biological replicates (n=3) and the same as those presented in Figure S10. ND, not detected. Percent of total molecules in treatment was calculated using mean values and the Avogadro constant. Values were rounded to the nearest hundredth. Average molecules per cell was calculated using 115,000 cells, assuming one doubling. Values were rounded to the nearest thousand.

Oligonucleotide	HEL cell lysate		Percent of total molecules in treatment		Average molecules per cell	
	500 nM	2 µM	500 nM	2 µM	500 nM	2 µM
ON1	ND	ND	--	--	--	--
ON2	9.73E+10 ± 2.69E+10	1.46E+11 ± 1.96E+10	0.06	0.02	846,000	1,270,000
ON3	ND	ND	--	--	--	--
ON4	4.40E+10 ± 1.17E+10	7.37E+10 ± 1.41E+10	0.03	0.01	383,000	641,000
ON5	4.31E+10 ± 1.00E+10	8.17E+10 ± 2.14E+10	0.03	0.01	375,000	710,000
ON6	3.26E+10 ± 4.82E+09	4.99E+10 ± 6.93E+09	0.02	0.01	283,000	434,000

Table S2. Oligonucleotides detected in HEL nuclear lysate following free uptake for 24 h. Data are mean molecules ± SEM for three biological replicates (n=3) and the same as those presented in Figure S10. ND, not detected. Percent of total molecules in cells was calculated using mean values in Table S1. Values were rounded to the nearest percent. Average molecules per nucleus was calculated using 115,000 cells, assuming one doubling. Values were rounded to the nearest thousand.

Oligonucleotide	HEL nuclear lysate		Percent of total molecules in cells		Average molecules per nucleus	
	500 nM	2 µM	500 nM	2 µM	500 nM	2 µM
ON1	ND	ND	--	--	--	--
ON2	5.93E+09 ± 8.55E+08	9.70E+09 ± 2.28E+09	6	7	52,000	84,000
ON3	ND	ND	--	--	--	--
ON4	ND	ND	--	--	--	--
ON5	1.54E+10 ± 4.99E+09	2.25E+10 ± 7.09E+09	36	28	134,000	196,000
ON6	ND	ND	--	--	--	--

Table S3. Oligonucleotides detected in HEL cytoplasm following free uptake for 24 h. Data are mean molecules ± SEM for three biological replicates (n=3) and the same as those presented in Figure S10. ND, not detected. Percent of total molecules in cells was calculated using mean values in Table S1. Values were rounded to the nearest percent. Average molecules per cell was calculated using 115,000 cells, assuming one doubling. Values were rounded to the nearest thousand.

Oligonucleotide	HEL cytoplasm		Percent of total molecules in cells		Average molecules per cell	
	500 nM	2 µM	500 nM	2 µM	500 nM	2 µM
ON1	ND	ND	--	--	--	--
ON2	ND	1.31E+11 ± 2.38E+10	--	90	--	1,139,000
ON3	ND	ND	--	--	--	--
ON4	ND	ND	--	--	--	--
ON5	3.25E+10 ± 6.25E+09	5.60E+10 ± 1.97E+09	75	69	283,000	487,000
ON6	ND	4.02E+10 ± 6.82E+08	--	81	--	350,000

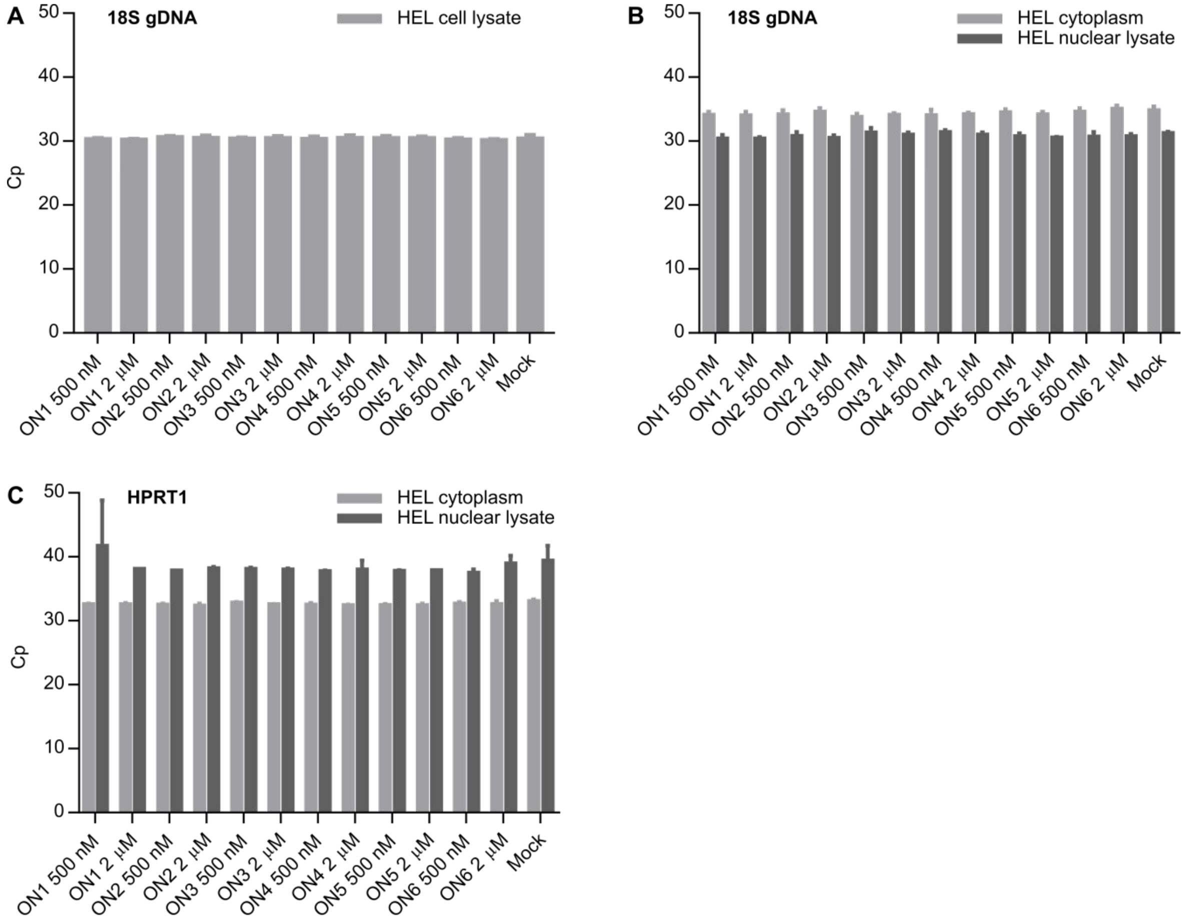

Figure S11. Quantification of 18S gDNA and HPRT1 following free uptake of the oligonucleotides in HEL cells for 24 h. (A) Quantification of 18S gDNA in HEL cell lysate. (B) Quantification of 18S gDNA in HEL cytoplasm and nuclear lysate. (C) Quantification of HPRT1 in HEL cytoplasm and nuclear lysate. In panels (A) and (B), data are mean Cp values ± SD for three biological replicates (n=3). In panel (C), HEL cytoplasm data are mean Cp values ± SD for three biological replicates (n=3). HPRT1 was not always detected in nuclear lysate; therefore, data are single Cp values or mean Cp values ± SD for two or three biological replicates (n=2 or n=3).

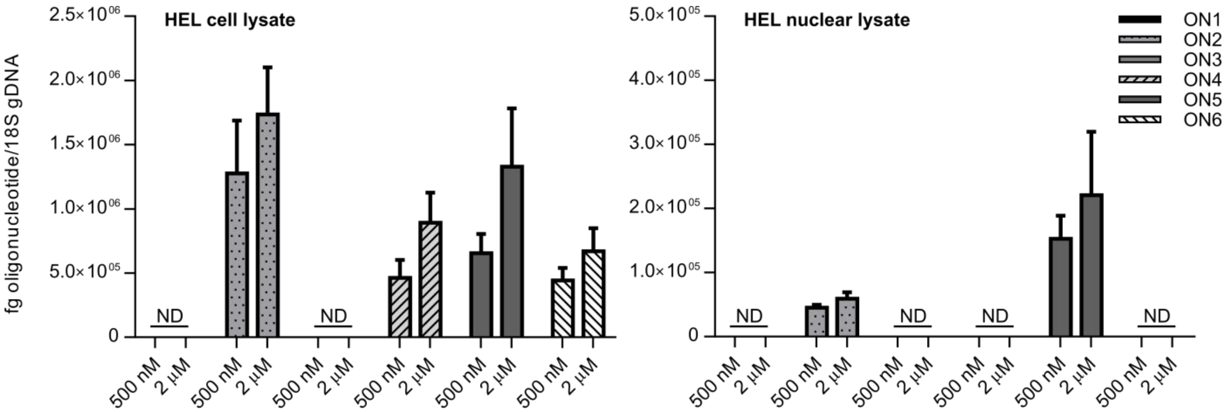

Figure S12. Femtograms (fg) oligonucleotide per unit 18S gDNA detected following free uptake in HEL cells for 24 h. Data are mean fg per unit 18S gDNA ± SEM for three biological replicates (n=3). ND, not detected.

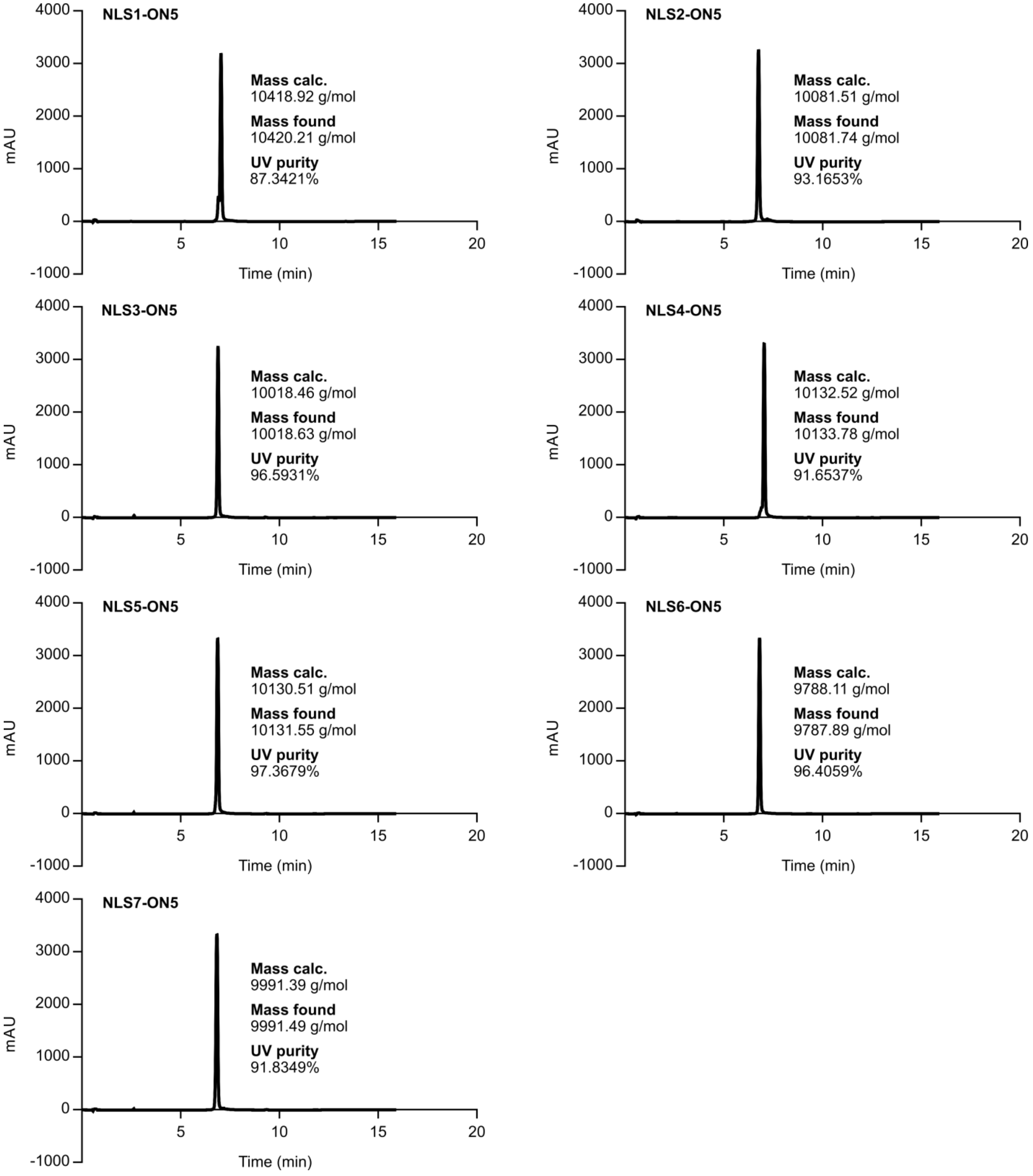

Figure S13. LC-MS chromatograms for conjugates. The mass for the 5'-capped-maleimide-modified oligonucleotide was calculated using the Oligowizard Nucleic Acid Calculator, an online tool available at http://oligowizard.com/. The mass of each peptide was provided by GenScript (Piscataway, New Jersey, U.S.). UV purity is expressed as percent area under the peak.

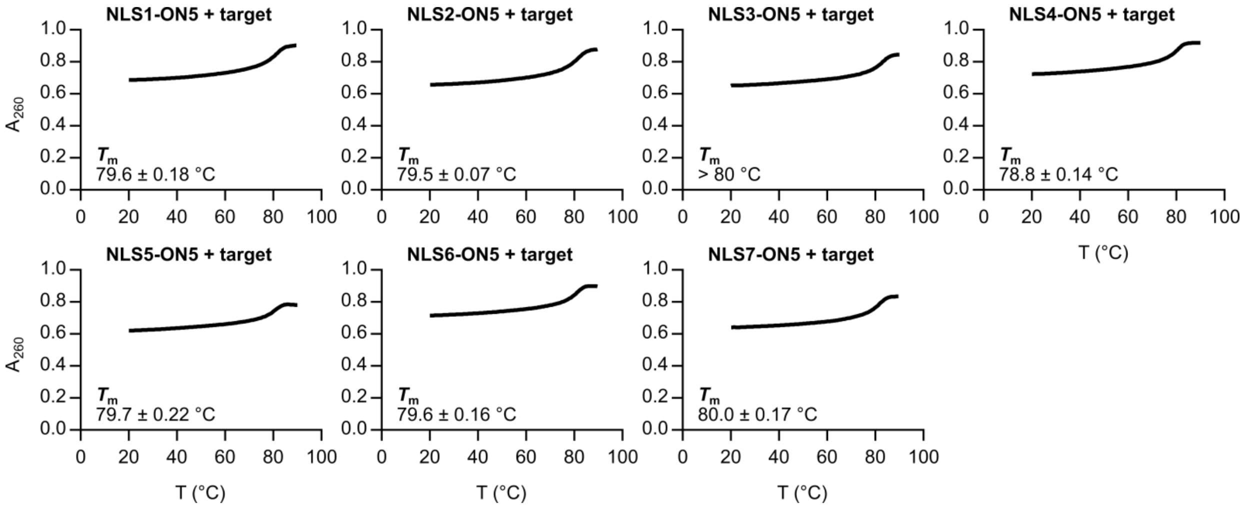

Figure S14. Melting data for conjugates. Representative datasets are shown for the conjugates paired with a 22-nt RNA representing the c.315-48C FECH pre-mRNA in 100 mM NaCl, 10 mM phosphate, 0.1 mM Na_2EDTA, pH 7.0. T_ms were determined from first derivative analyses of nonlinear fit melting curves. T_ms are mean ± SD for three technical replicates. The upper baseline for NLS3-ON5 and the RNA target was too short to fit; therefore, a T_m of > 80 °C was estimated from the maximum of the first derivative of the raw data.

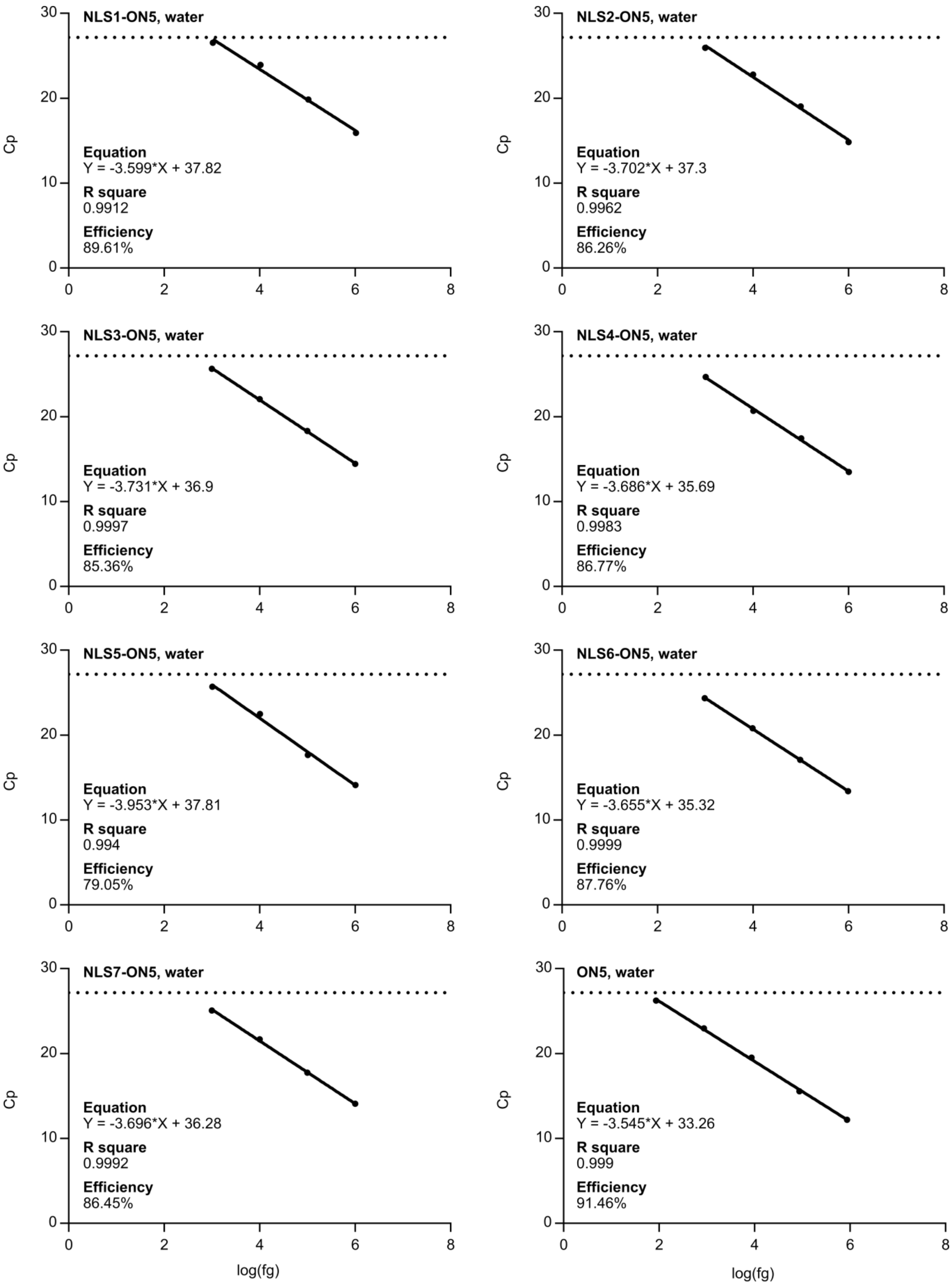

Figure S15. Linear fit CL-qPCR calibration curves for conjugates in water. Data are mean Cp values ± SD for three technical replicates. Efficiencies were calculated using the ThermoFisher Scientific qPCR Efficiency Calculator.

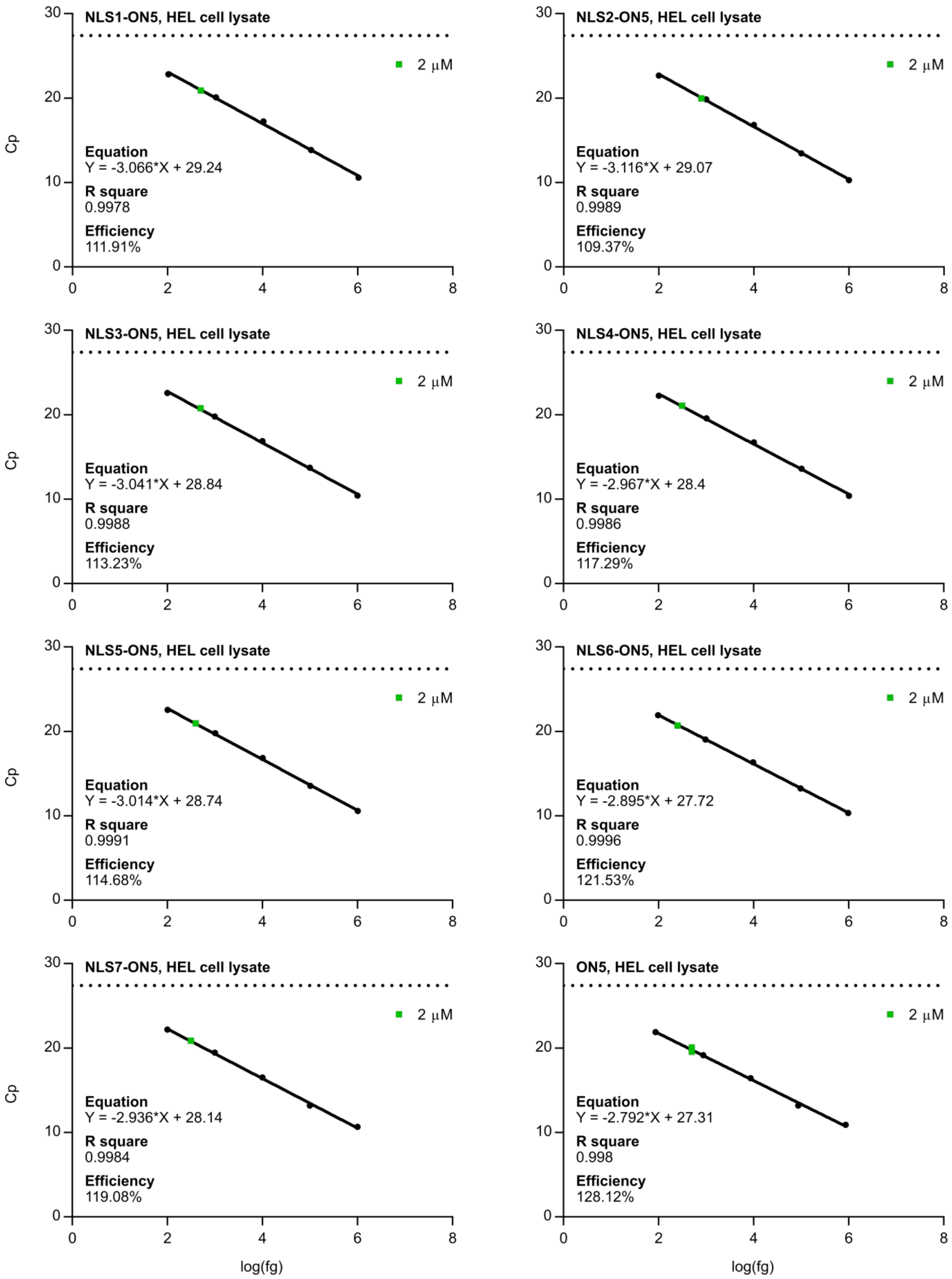

Figure S16. Linear fit CL-qPCR calibration curves for conjugates in HEL cell lysate. The Cp values measured in HEL cell lysates following free uptake for 24 h are overlaid. Data are mean Cp values ± SD for three biological replicates (n=3). Efficiencies were calculated using the ThermoFisher Scientific qPCR Efficiency Calculator.

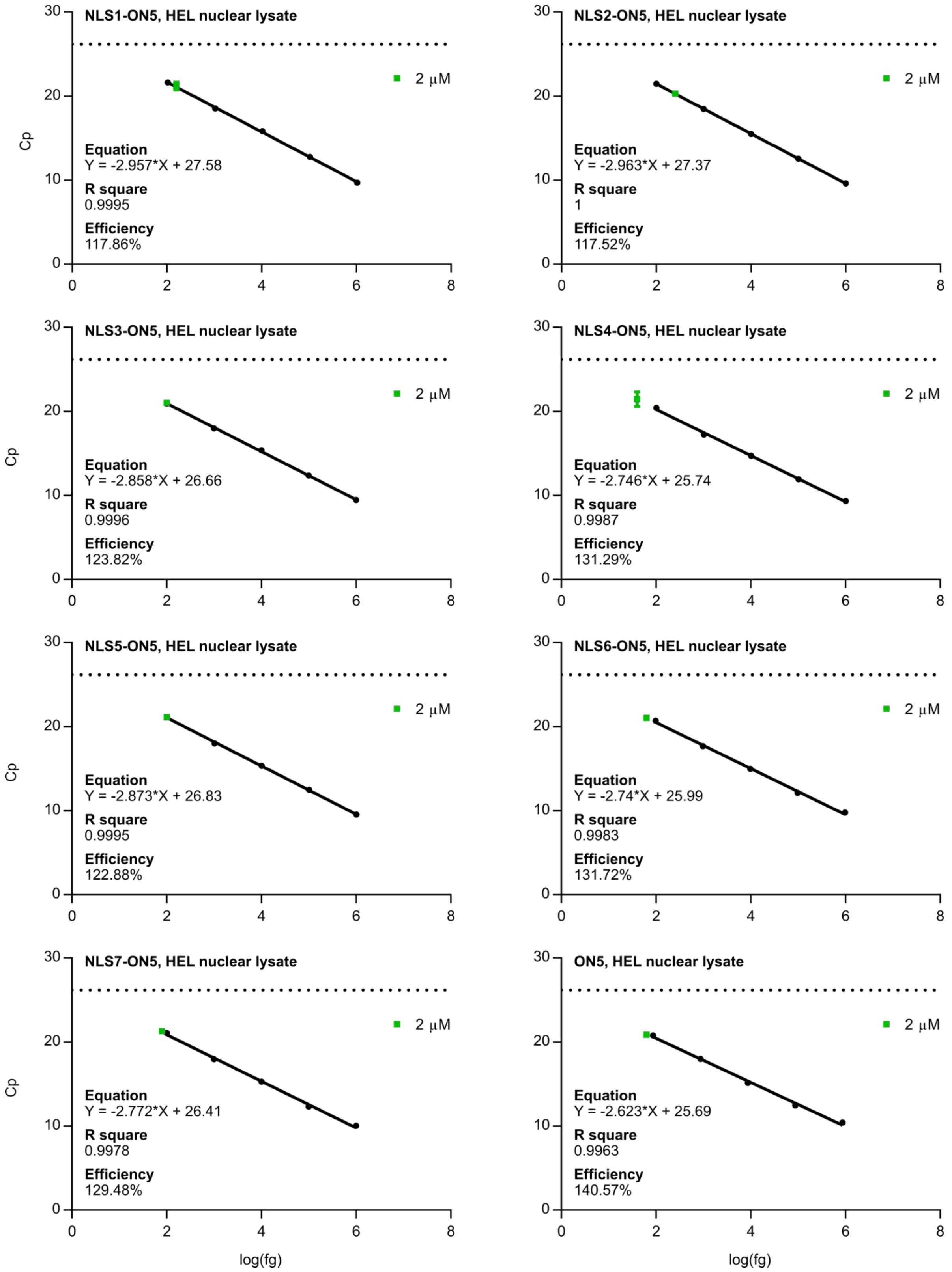

Figure S17. Linear fit CL-qPCR calibration curves for conjugates in HEL nuclear lysate. The Cp values measured in HEL nuclear lysates following free uptake for 24 h are overlaid. Data are mean Cp values ± SD for three biological replicates (n=3). Efficiencies were calculated using the ThermoFisher Scientific qPCR Efficiency Calculator.

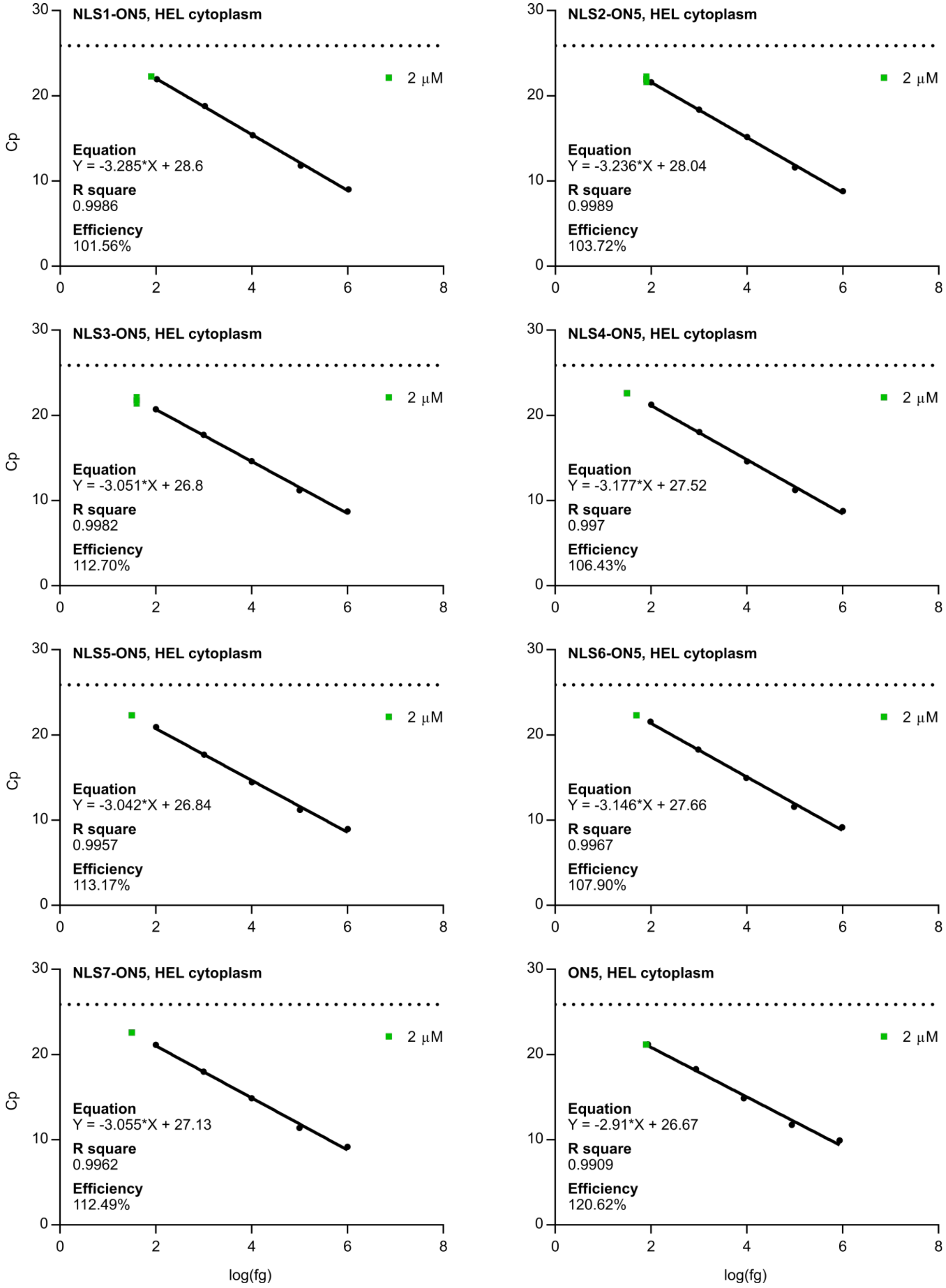

Figure S18. Linear fit CL-qPCR calibration curves for conjugates in HEL cytoplasm. The Cp values measured in HEL cytoplasm following free uptake for 24 h are overlaid. Data are mean Cp values ± SD for two or three biological replicates (n=2 or n=3). Efficiencies were calculated using the ThermoFisher Scientific qPCR Efficiency Calculator.

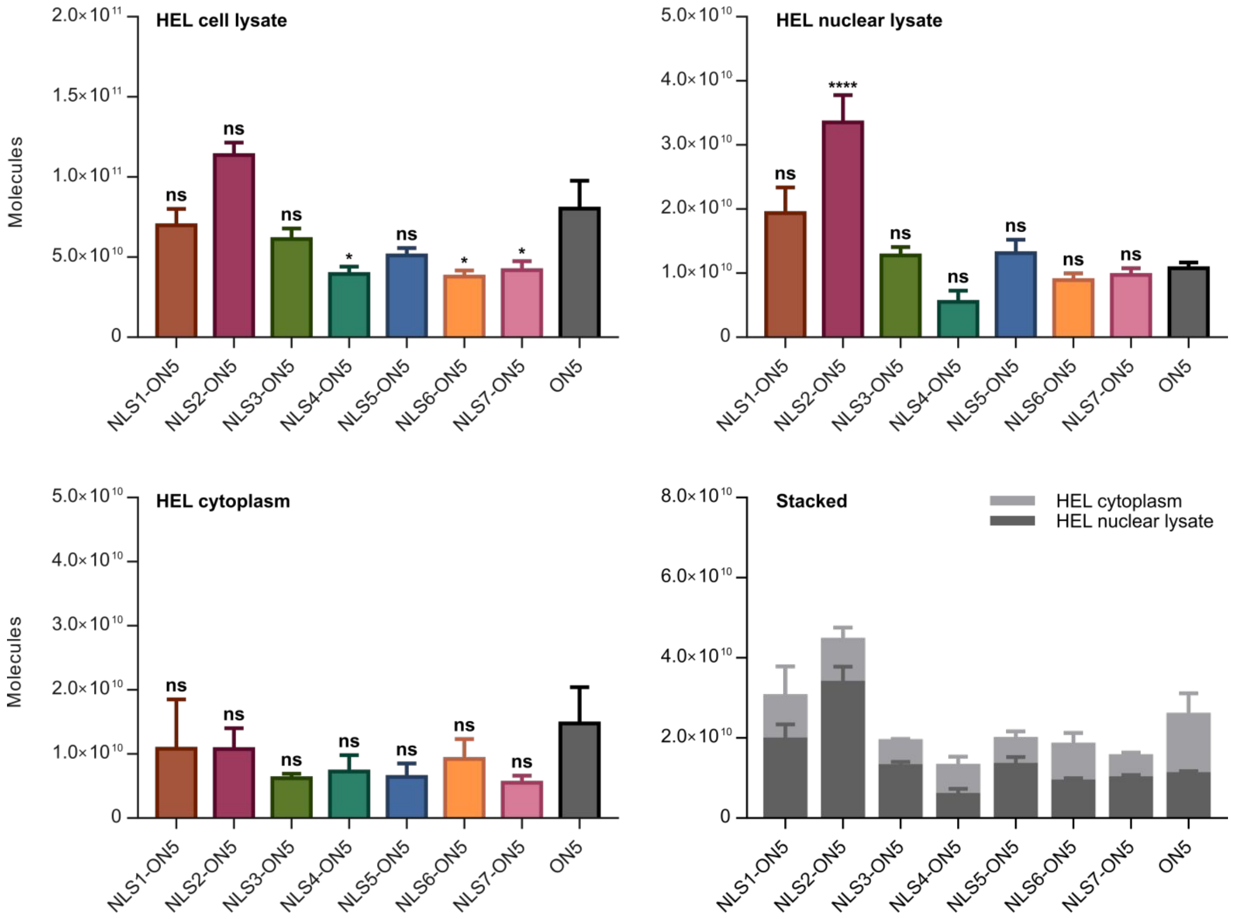

Figure S19. Oligonucleotide conjugates detected following free uptake at 2 µM for 24 h. Oligonucleotide conjugates detected in HEL cell lysate (*top left*), nuclear lysate (*top right*), and cytoplasm (*bottom left*). A stacked view (*bottom right*) shows differences in subcellular localization for the conjugates. HEL cell and nuclear lysate data are mean molecules ± SEM for three biological replicates (n=3). HEL cytoplasm data are mean molecules ± SEM for two or three biological replicates (n=2 or n=3). Statistics are one-way ANOVA with Dunnett's multiple comparisons test against ON5, α=0.05: ns, not significant; * P ≤ 0.05; ** P ≤ 0.01; *** P ≤ 0.001; **** P ≤ 0.0001.

Table S4. Oligonucleotide conjugates detected in HEL cell lysate following free uptake at 2 µM for 24 h. Data are mean molecules ± SEM for three biological replicates (n=3) and the same as those presented in Figure S19. Percent of total molecules in treatment was calculated using mean values and the Avogadro constant. Values were rounded to the nearest hundredth. Average molecules per cell was calculated using 115,000 cells, assuming one doubling. Values were rounded to the nearest thousand.

Conjugate	HEL cell lysate	Percent of total molecules in treatment	Average molecules per cell
NLS1-ON5	7.00E+10 ± 1.00E+10	0.01	607,000
NLS2-ON5	1.14E+11 ± 7.91E+09	0.02	991,000
NLS3-ON5	6.12E+10 ± 6.70E+09	0.01	532,000
NLS4-ON5	3.95E+10 ± 4.50E+09	0.01	343,000
NLS5-ON5	5.10E+10 ± 4.75E+09	0.01	443,000
NLS6-ON5	3.79E+10 ± 3.84E+09	0.01	330,000
NLS7-ON5	4.18E+10 ± 5.68E+09	0.01	363,000
ON5	8.01E+10 ± 1.73E+10	0.01	697,000

Table S5. Oligonucleotide conjugates detected in HEL nuclear lysate following free uptake at 2 µM for 24 h. Data are mean molecules ± SEM for three biological replicates (n=3) and the same as those presented in Figure S19. Percent of total molecules in cells was calculated using mean values in Table S4. Values were rounded to the nearest percent. Average molecules per nucleus was calculated using 115,000 cells, assuming one doubling. Values were rounded to the nearest thousand.

Conjugate	HEL nuclear lysate	Percent of total molecules in cells	Average molecules per nucleus
NLS1-ON5	1.94E+10 ± 4.00E+09	28	169,000
NLS2-ON5	3.35E+10 ± 4.23E+09	29	291,000
NLS3-ON5	1.28E+10 ± 1.28E+09	21	111,000
NLS4-ON5	5.54E+09 ± 1.75E+09	14	48,000
NLS5-ON5	1.31E+10 ± 2.13E+09	26	114,000
NLS6-ON5	8.91E+09 ± 1.06E+09	24	77,000
NLS7-ON5	9.75E+09 ± 1.06E+09	23	85,000
ON5	1.08E+10 ± 9.37E+08	13	94,000

Table S6. Oligonucleotide conjugates detected in HEL cytoplasm following free uptake at 2 µM for 24 h. Data are mean molecules ± SEM for two or three biological replicates (n=2 or n=3) and the same as those presented in Figure S19. Percent of total molecules in cells was calculated using mean values in Table S4. Values were rounded to the nearest percent. Average molecules per cell was calculated using 115,000 cells, assuming one doubling. Values were rounded to the nearest thousand.

Conjugate	HEL cytoplasm	Percent of total molecules in cells	Average molecules per cell
NLS1-ON5	1.09E+10 ± 7.68E+09	16	95,000
NLS2-ON5	1.08E+10 ± 3.21E+09	9	94,000
NLS3-ON5	6.21E+09 ± 7.00E+08	10	54,000
NLS4-ON5	7.26E+09 ± 2.54E+09	18	63,000
NLS5-ON5	6.45E+09 ± 2.07E+09	13	56,000
NLS6-ON5	9.24E+09 ± 3.13E+09	24	80,000
NLS7-ON5	5.50E+09 ± 1.10E+09	13	48,000
ON5	1.47E+10 ± 5.66E+09	18	128,000

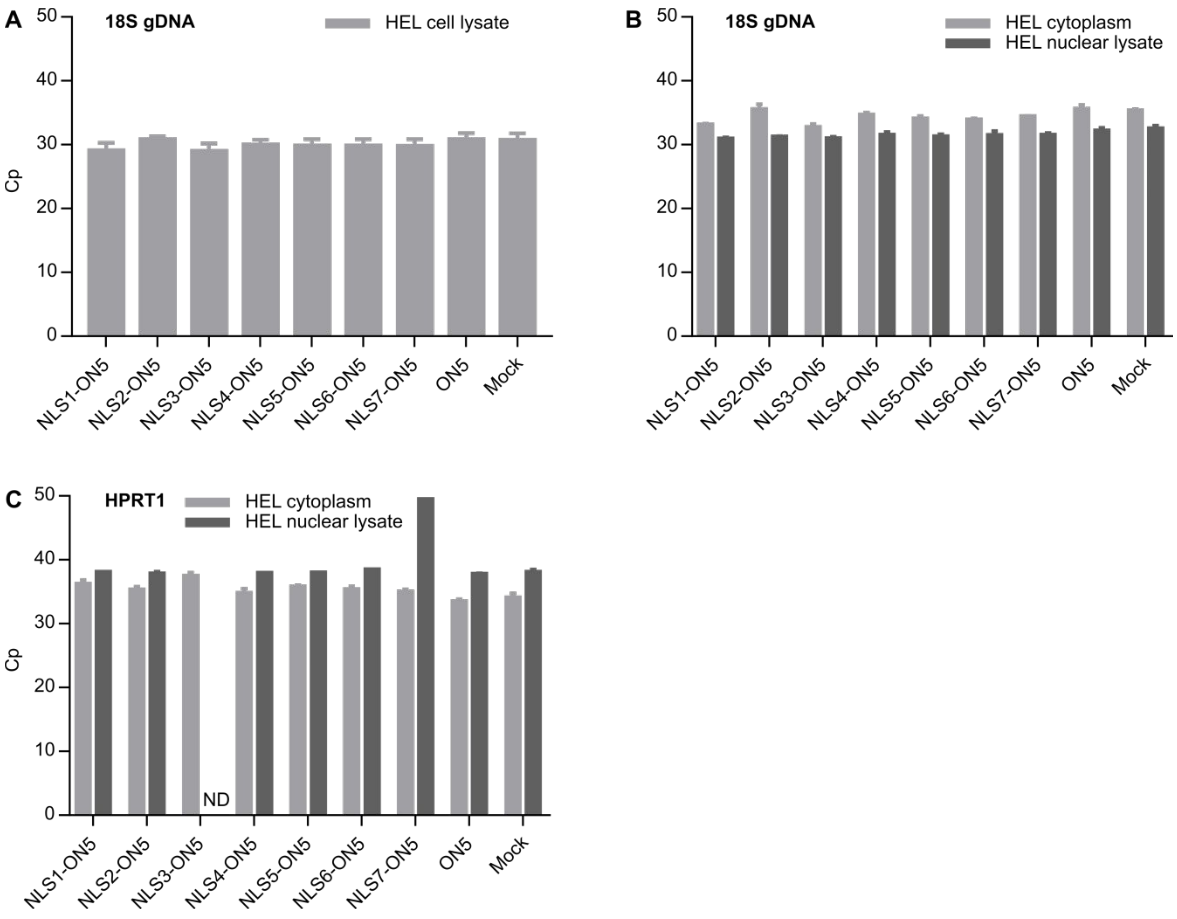

Figure S20. Quantification of 18S gDNA and HPRT1 following free uptake of the conjugates in HEL cells at 2 µM for 24 h. (A) Quantification of 18S gDNA in HEL cell lysate. (B) Quantification of 18S gDNA in HEL cytoplasm and nuclear lysate. (C) Quantification of HPRT1 in HEL cytoplasm and nuclear lysate. In panels (A) and (B), data are mean Cp values ± SD for three biological replicates (n=3). In panel (C), HEL cytoplasm data are mean Cp values ± SD for three biological replicates (n=3). HPRT1 was not always detected in nuclear lysate; therefore, data are single Cp values or mean Cp values ± SD for two biological replicates (n=2). ND, not detected.

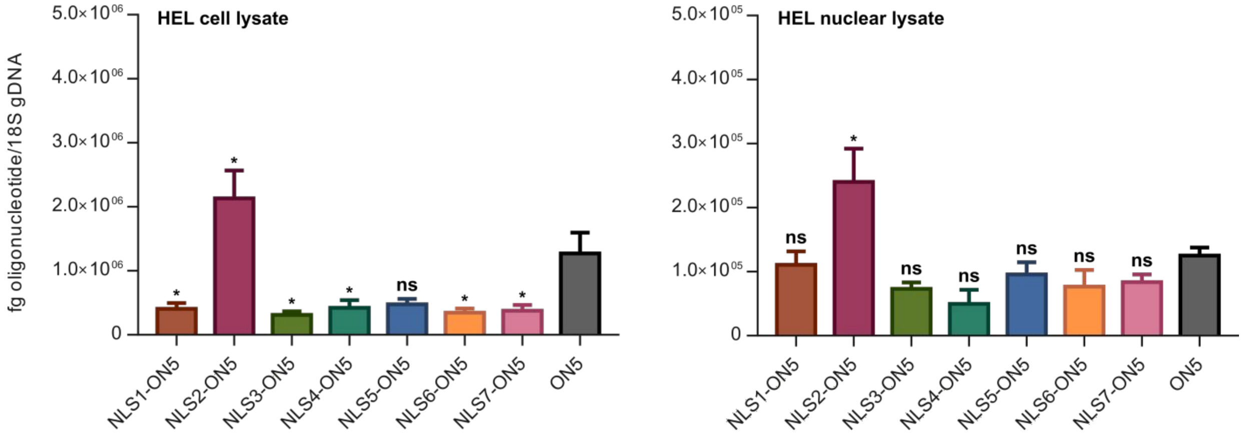

Figure S21. Femtograms (fg) conjugate per unit 18S gDNA detected following free uptake of the conjugates in HEL cells at 2 µM for 24 h. Data are mean fg per unit 18S gDNA ± SEM for three biological replicates (n=3). Statistics are one-way ANOVA with Dunnett's multiple comparisons test against ON5, α=0.05: ns, not significant; * P ≤ 0.05; ** P ≤ 0.01; *** P ≤ 0.001; **** P ≤ 0.0001.

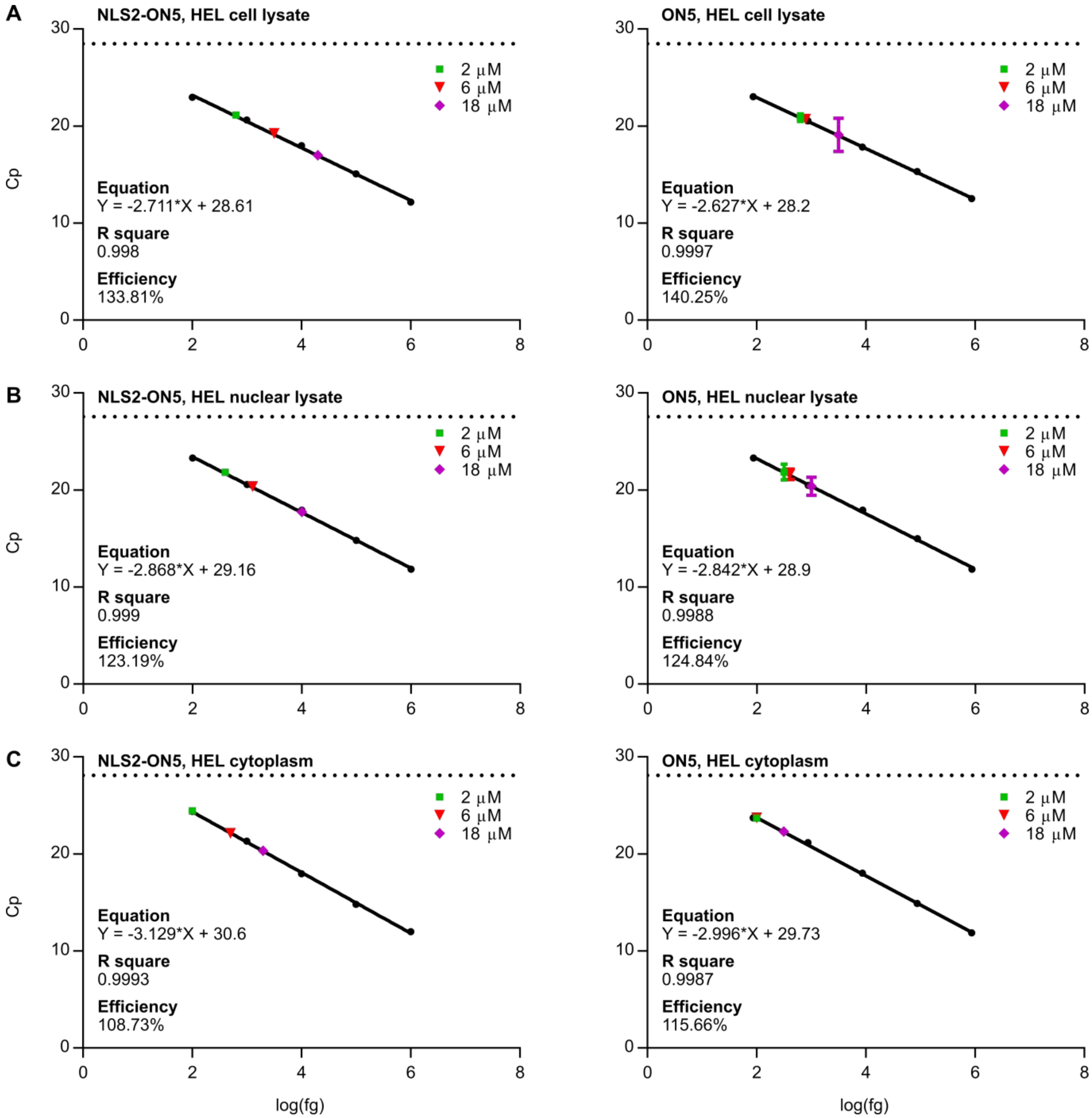

Figure S22. Linear fit CL-qPCR calibration curves for the lead conjugate. (A) Linear fit CL-qPCR calibration curve for the lead conjugate in HEL cell lysate. (B) Linear fit CL-qPCR calibration curve for the lead conjugate in HEL nuclear lysate. (C) Linear fit CL-qPCR calibration curve for the lead conjugate in HEL cytoplasm. The Cp values measured in each compartment following free uptake of the conjugate in HEL cells for 24 h are overlaid. Data are mean Cp values ± SD for three biological replicates (n=3). Efficiencies were calculated using the ThermoFisher Scientific qPCR Efficiency Calculator.

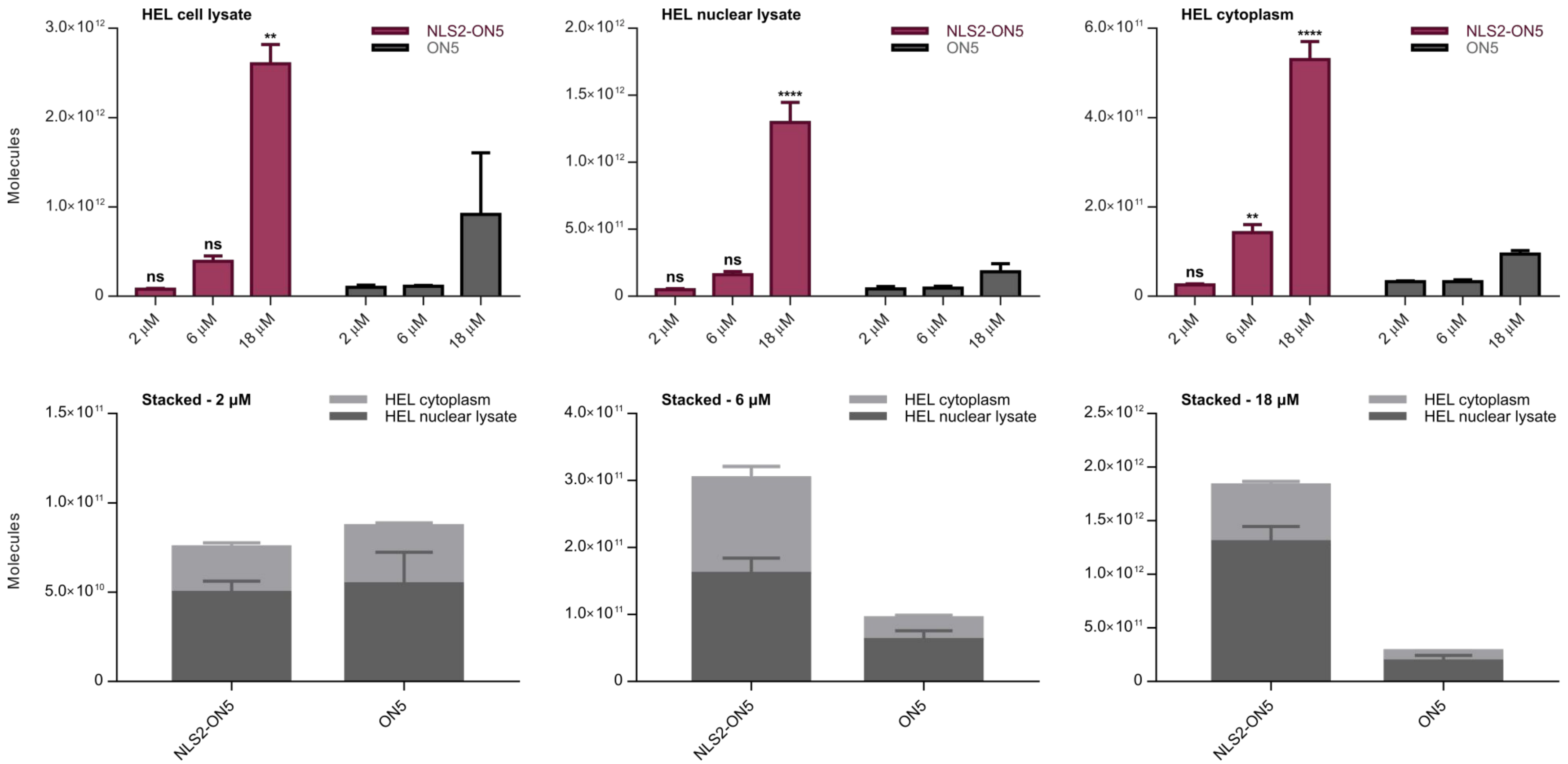

Figure S23. Lead oligonucleotide conjugate detected following free uptake of the conjugate in HEL cells for 24 h. Statistics are two-way ANOVA with Sidak's multiple comparisons test against ON5, α=0.05: ns, not significant; * P ≤ 0.05, ** P ≤ 0.01, *** P ≤ 0.001, **** P ≤ 0.0001. Stacked views show differences in subcellular localization for the conjugate at different concentrations. Data are mean molecules ± SEM for three biological replicates (n=3).

Table S7. Lead oligonucleotide conjugate detected in HEL cell lysate following free uptake of the conjugate in HEL cells for 24 h. Data are mean molecules ± SEM for three biological replicates (n=3) and the same as those presented in Figure S23. Percent of total molecules in treatment was calculated using mean values and the Avogadro constant. Values were rounded to the nearest hundredth. Average molecules per cell was calculated using 115,000 cells, assuming one doubling. Values were rounded to the nearest thousand.

Conjugate	HEL cell lysate			Percent of total molecules in treatment			Average molecules per cell		
	2 µM	6 µM	18 µM	2 µM	6 µM	18 µM	2 µM	6 µM	18 µM
NLS2-ON5	7.82E+10 ± 9.43E+09	3.92E+11 ± 6.01E+10	2.60E+12 ± 2.13E+11	0.01	0.02	0.04	680,000	3,409,000	22,609,000
ON5	1.02E+11 ± 2.22E+10	1.11E+11 ± 8.61E+09	9.18E+11 ± 6.91E+11	0.02	0.01	0.02	887,000	965,000	7,983,000

Table S8. Lead oligonucleotide conjugate detected in HEL nuclear lysate following free uptake of the conjugate in HEL cells for 24 h. Data are mean molecules ± SEM for three biological replicates (n=3) and the same as those presented in Figure S23. Percent of total molecules in cells was calculated using mean values in Table S7. Values were rounded to the nearest percent. Average molecules per nucleus was calculated using 115,000 cells, assuming one doubling. Values were rounded to the nearest thousand.

Conjugate	HEL nuclear lysate			Percent of total molecules in cells			Average molecules per nucleus		
	2 µM	6 µM	18 µM	2 µM	6 µM	18 µM	2 µM	6 µM	18 µM
NLS2-ON5	4.94E+10 ± 6.75E+09	1.61E+11 ± 2.38E+10	1.30E+12 ± 1.50E+11	63	41	50	430,000	1,400,000	11,304,000
ON5	5.42E+10 ± 1.81E+10	6.13E+10 ± 1.46E+10	1.84E+11 ± 5.92E+10	53	55	20	471,000	533,000	1,600,000

Table S9. Lead oligonucleotide conjugate detected in HEL cytoplasm following free uptake of the conjugate in HEL cells for 24 h. Data are mean molecules ± SEM for three biological replicates (n=3) and the same as those presented in Figure S23. Percent of total molecules in cells was calculated using mean values in Table S7. Values were rounded to the nearest percent. Average molecules per cell was calculated using 115,000 cells, assuming one doubling. Values were rounded to the nearest thousand.

Conjugate	HEL cytoplasm			Percent of total molecules in cells			Average molecules per cell		
	2 µM	6 µM	18 µM	2 µM	6 µM	18 µM	2 µM	6 µM	18 µM
NLS2-ON5	2.55E+10 ± 2.70E+09	1.42E+11 ± 1.85E+10	5.30E+11 ± 4.03E+10	33	36	20	222,000	1,235,000	4,609,000
ON5	3.26E+10 ± 1.89E+09	3.26E+10 ± 4.57E+09	9.45E+10 ± 7.85E+09	32	29	10	283,000	283,000	822,000

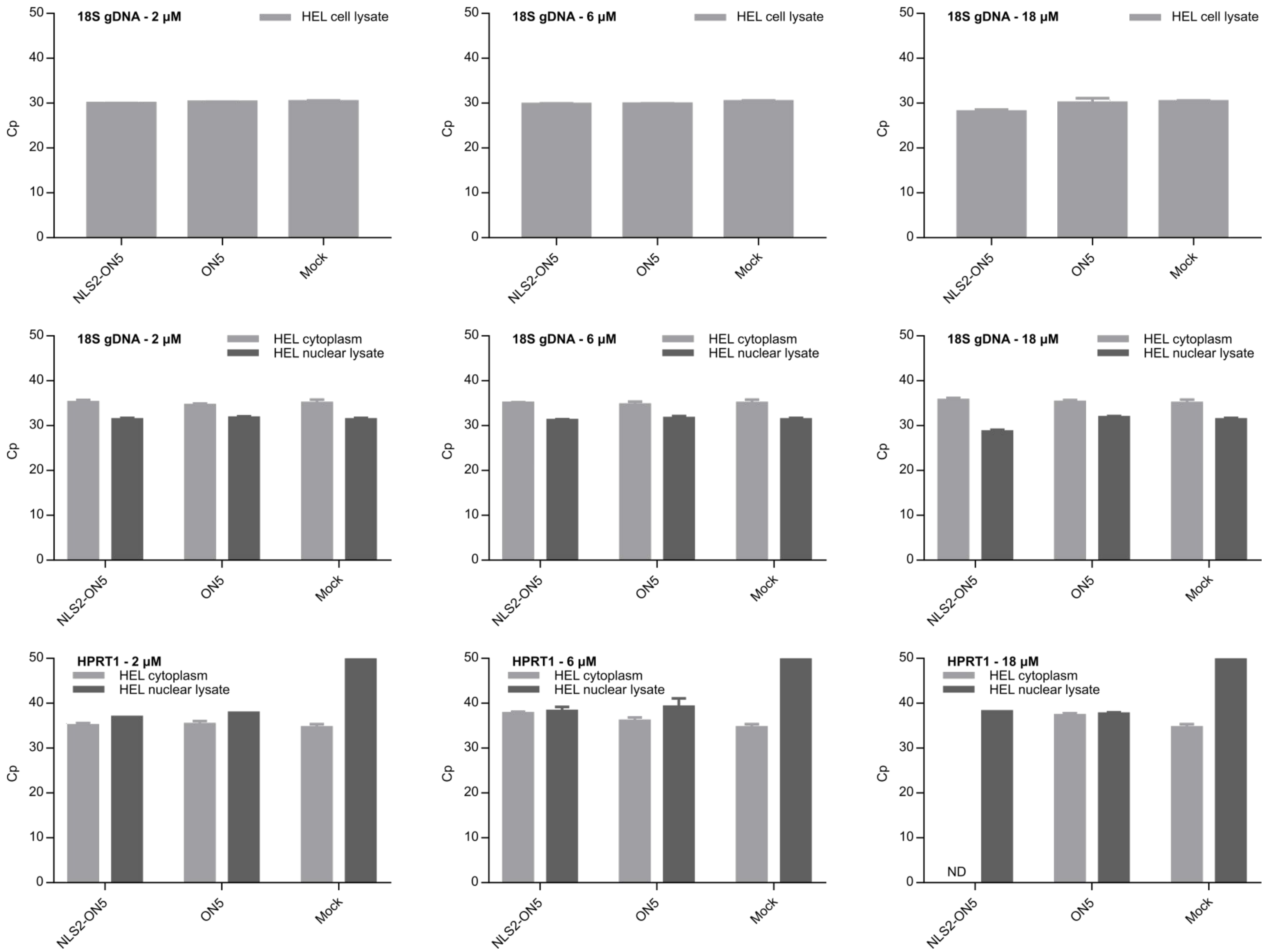

Figure S24. Quantification of 18S gDNA and HPRT1 following free uptake of the lead conjugate in HEL cells for 24 h. 18S gDNA data are mean Cp values ± SD for three biological replicates (n=3). HPRT1 was not always detected in nuclear lysate; therefore, data are single Cp values or mean Cp values ± SD for two biological replicates (n=2). ND, not detected.

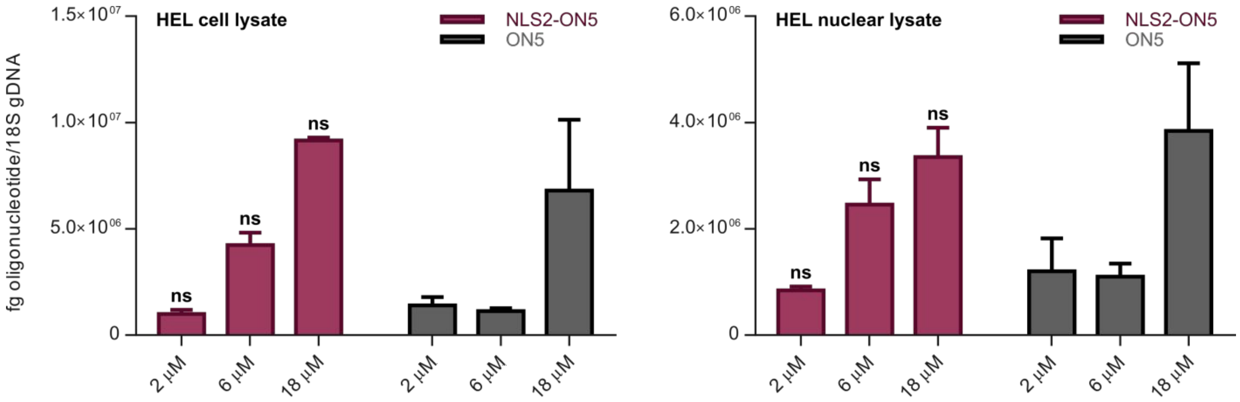

Figure S25. Femtograms (fg) lead conjugate per unit 18S gDNA detected following free uptake of the conjugate in HEL cells for 24 h. Data are mean fg per unit 18S gDNA ± SEM for three biological replicates (n=3). Statistics are two-way ANOVA with Sidak's multiple comparisons test against ON5, α=0.05: ns, not significant; * P ≤ 0.05; ** P ≤ 0.01; *** P ≤ 0.001; **** P ≤ 0.0001.

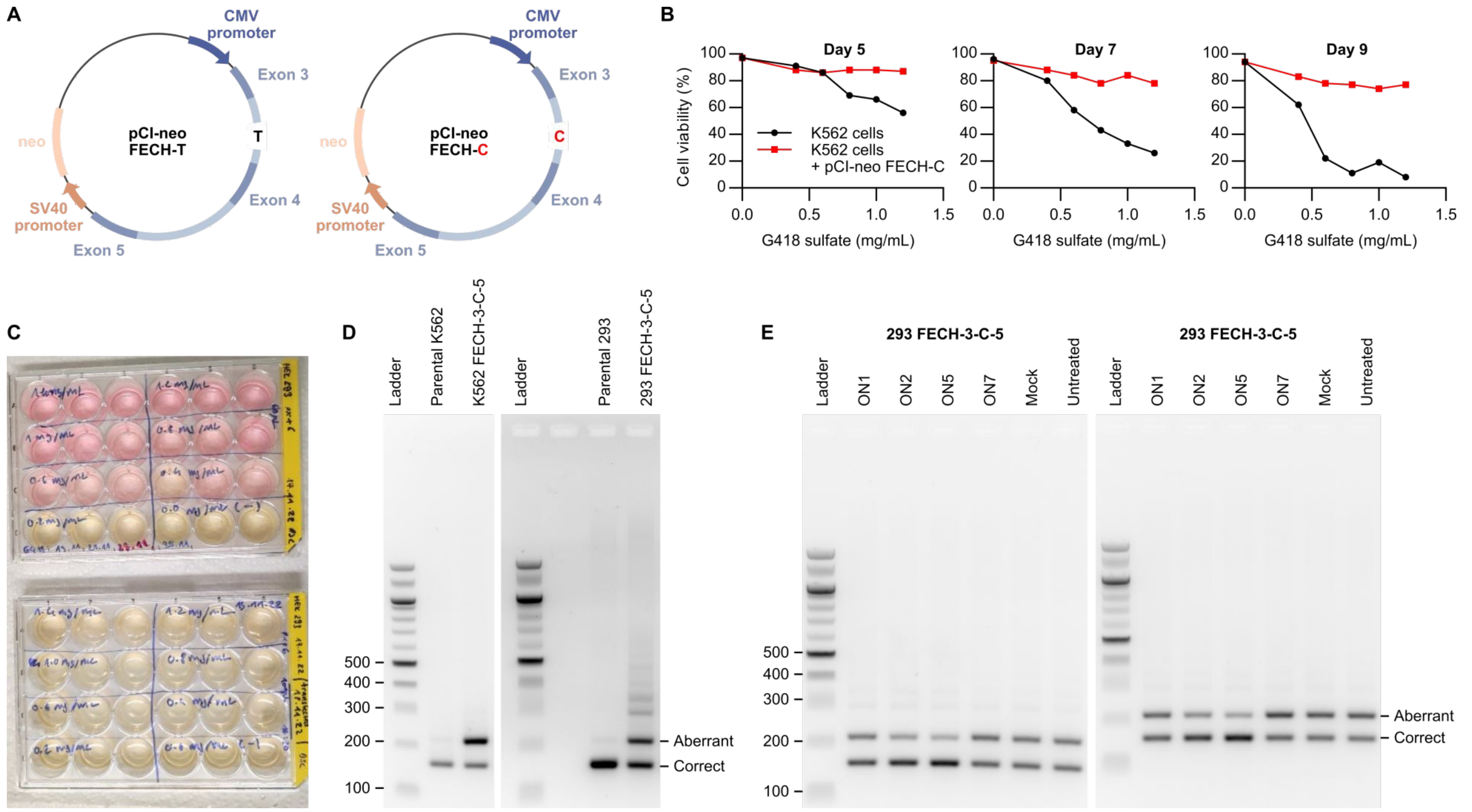

Figure S26. Generation of FECH minigenes and FECH-3-C-5 stable cell lines. (A) Schematic of FECH minigenes. *Left*, A FECH-T minigene with exons 3 to 5 was generated from K562 genomic DNA and cloned into a pCI-neo vector under the control of a constitutive CMV promoter. *Right*, A FECH-C minigene was generated by site-directed mutagenesis of the FECH-T minigene. (B) G418 selection in K562 cells. K562 cells were sensitive to 0.4 mg/mL G418, and most cells died at 0.6 mg/mL G418, after nine days. K562 cells transfected with the FECH-C minigene plasmid were resistant to G418 at all concentrations tested over time. Data are percent cell viability for one technical replicate. (C) G418 selection in 293 cells. *Top*, 293 cells after nine days' treatment with G418 at different concentrations. *Bottom*, 293 cells transfected with the FECH-C minigene plasmid after nine days' treatment with G418 at the same concentrations. (D) Splicing of FECH pre-mRNA in the FECH-3-C-5 stable cell lines. *Left*, K562 FECH-3-C-5 cells produce high amounts of the 63-nt longer aberrant FECH transcript. *Right*, 293 FECH-3-C-5 cells produce roughly equal amounts of the correct and aberrant FECH transcripts. (E) Splice-switching activities of selected SSOs following transfection in 293 FECH-3-C-5 cells. Transfections were performed using Lipofectamine 2000 and 10 nM oligonucleotide for 48 h. Mock was treated with Lipofectamine alone. *Left*, replicate 1. *Right*, replicate 2. In (D) and (E), the ladder was 5 µL Quick-Load Purple 100 bp DNA Ladder (New England BioLabs N0551S).

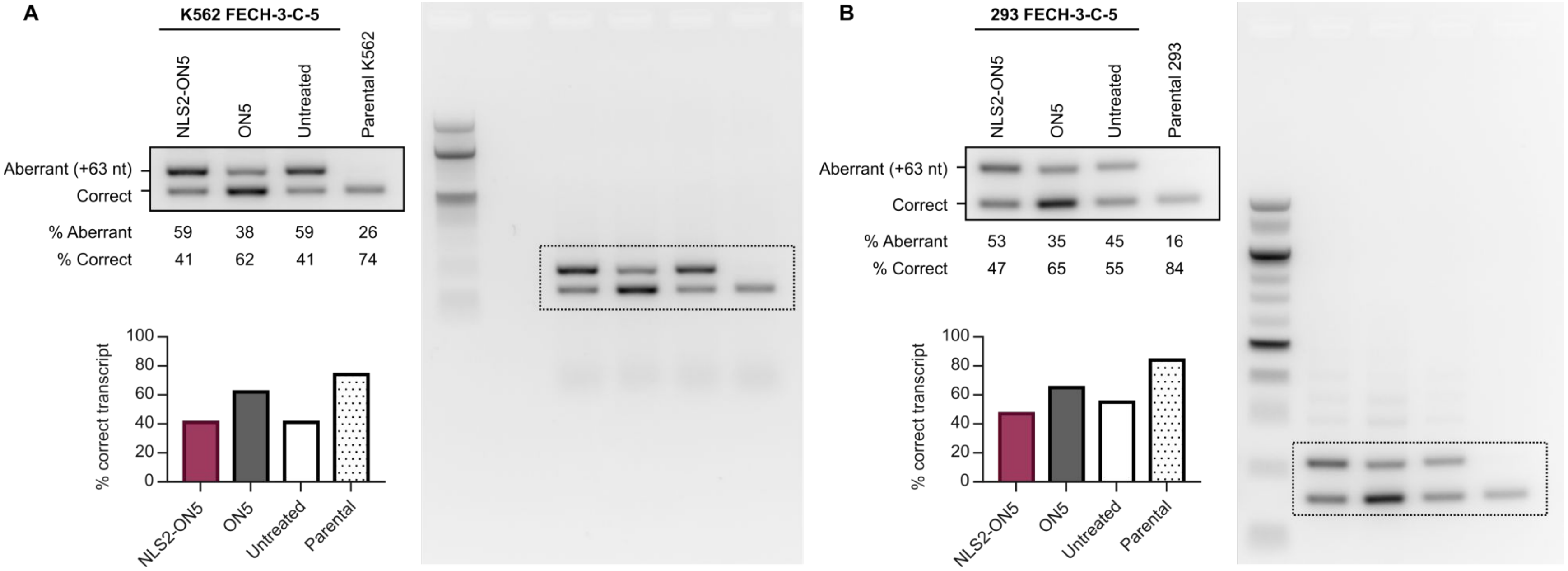

Figure S27. Splice-switching activities of the lead conjugate and parent SSO following free uptake. (A) Semi-quantitation (*left*) and raw image (*right*) of an agarose gel separating reverse transcribed and PCR amplified FECH transcripts extracted from K562 FECH-3-C-5 cells. (B) Semi-quantitation (*left*) and raw image (*right*) of an agarose gel separating reverse transcribed and PCR amplified FECH transcripts extracted from 293 FECH-3-C-5 cells. In (A) and (B), cells were treated with conjugate or oligonucleotide under free uptake conditions at 6 µM for 48 h. Untreated parental cell lines were included for reference. Percent (%) refers to percentage of total (*i.e.*, aberrant + correct) FECH transcript. The ladder was 5 µL Quick-Load Purple 100 bp DNA Ladder (New England BioLabs N0551S).

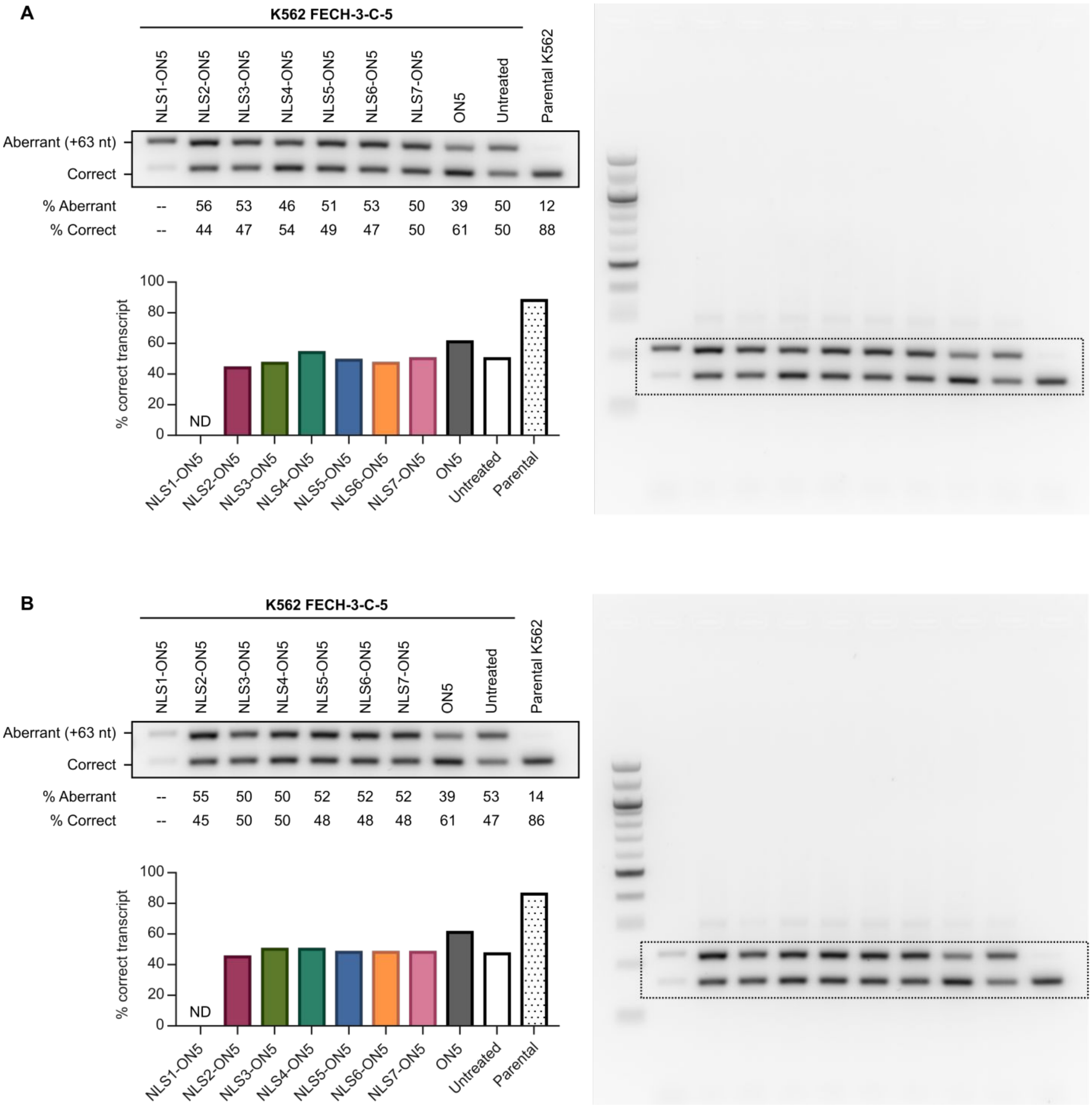

Figure S28. Splice-switching activities of all conjugates and parent SSO following free uptake. Panels (A) and (B) are replicate experiments. Each panel shows a semi-quantitation (*left*) and raw image (*right*) of an agarose gel separating reverse transcribed and PCR amplified FECH transcripts extracted from K562 FECH-3-C-5 cells. Cells were treated with conjugate or oligonucleotide under free uptake conditions at 6 µM for 48 h. Untreated parental cell lines were included for reference. Percent (%) refers to percentage of total (*i.e.*, aberrant + correct) FECH transcript. ND, not determined. The semi-quantitation of bands for NLS1-ON5 was not possible owing to a limited amount of RNA recovered from the cells treated with this compound. The ladder was 5 µL Quick-Load Purple 100 bp DNA Ladder (New England BioLabs N0551S).

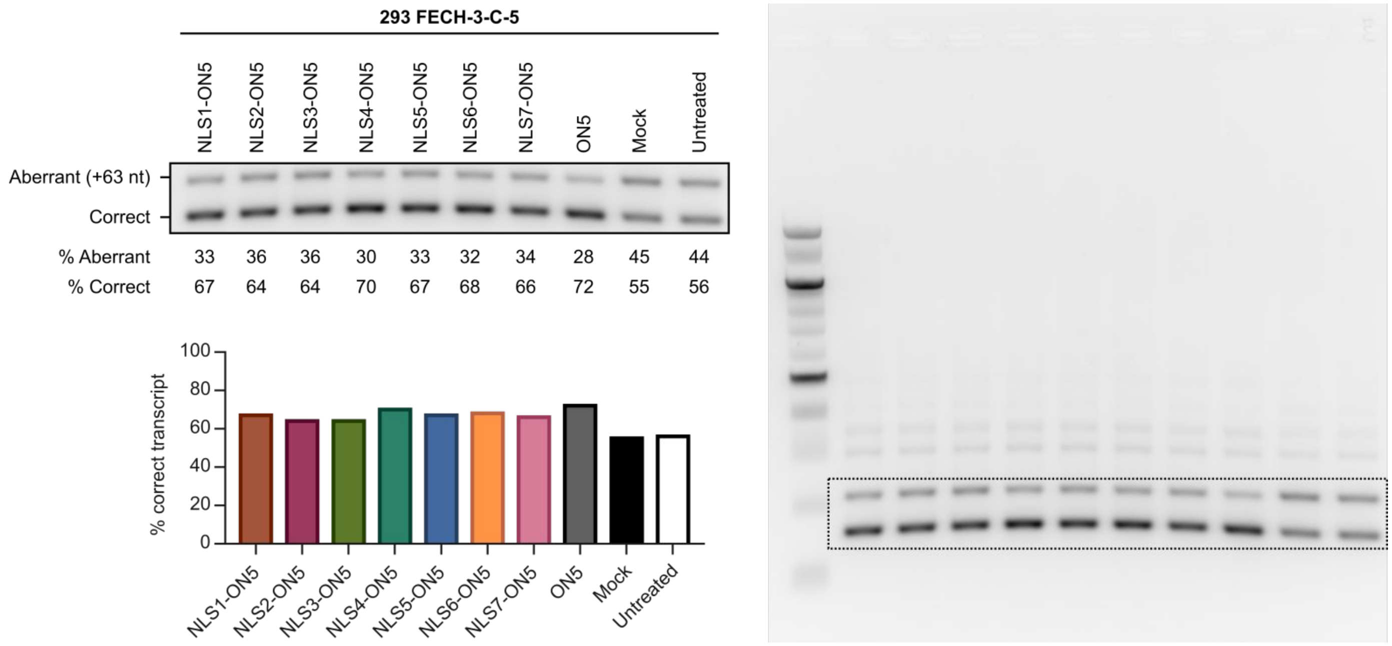

Figure S29. Splice-switching activities of all conjugates and parent SSO following transfection, replicate 1. Semi-quantitation (*left*) and raw image (*right*) of an agarose gel separating reverse transcribed and PCR amplified FECH transcripts extracted from 293 FECH-3-C-5 cells. Transfections were performed using Lipofectamine 2000 and 10 nM conjugate or oligonucleotide for 48 h. Mock was treated with Lipofectamine alone. Percent (%) refers to percentage of total (*i.e.*, aberrant + correct) FECH transcript. The ladder was 5 µL Quick-Load Purple 100 bp DNA Ladder (New England BioLabs N0551S).

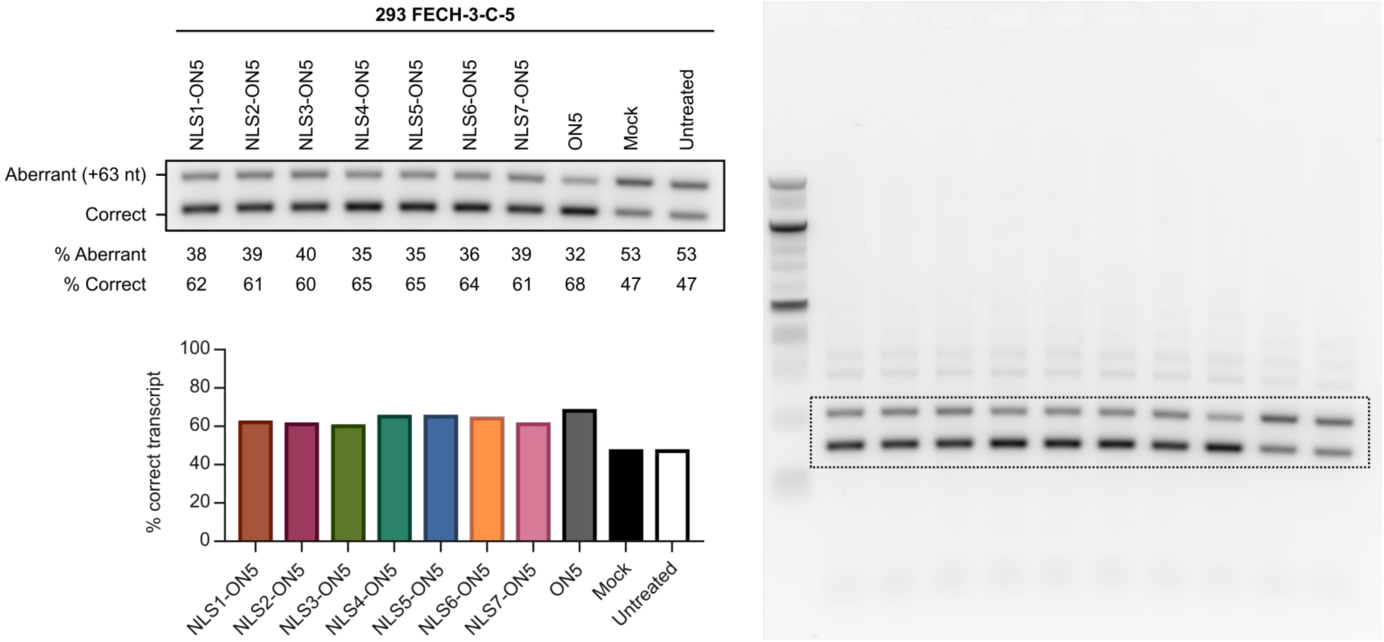

Figure S30. Splice-switching activities of all conjugates and parent SSO following transfection, replicate 2. Semi-quantitation (*left*) and raw image (*right*) of an agarose gel separating reverse transcribed and PCR amplified FECH transcripts extracted from 293 FECH-3-C-5 cells. Transfections were performed using Lipofectamine 2000 and 10 nM conjugate or oligonucleotide for 48 h. Mock was treated with Lipofectamine alone. Percent (%) refers to percentage of total (*i.e.*, aberrant + correct) FECH transcript. The ladder was 5 µL Quick-Load Purple 100 bp DNA Ladder (New England BioLabs N0551S).

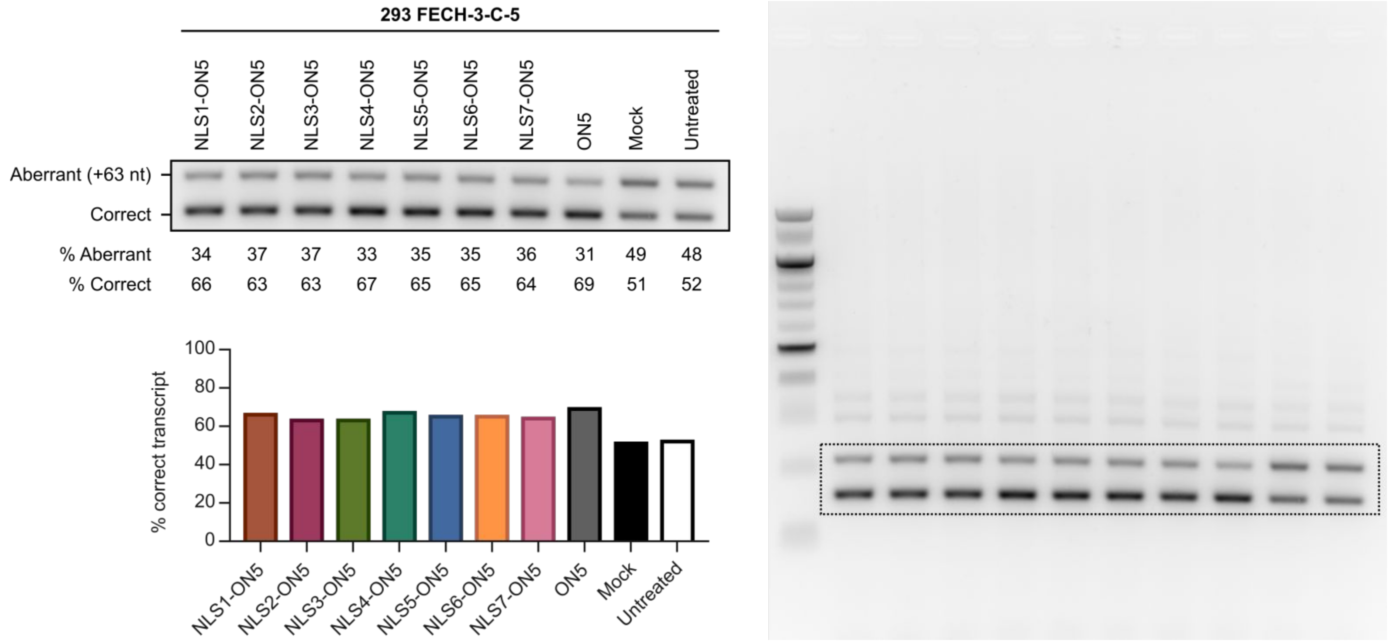

Figure S31. Splice-switching activities of all conjugates and parent SSO following transfection, replicate 3. Semi-quantitation (*left*) and raw image (*right*) of an agarose gel separating reverse transcribed and PCR amplified FECH transcripts extracted from 293 FECH-3-C-5 cells. Transfections were performed using Lipofectamine 2000 and 10 nM conjugate or oligonucleotide for 48 h. Mock was treated with Lipofectamine alone. Percent (%) refers to percentage of total (*i.e.*, aberrant + correct) FECH transcript. The ladder was 5 µL Quick-Load Purple 100 bp DNA Ladder (New England BioLabs N0551S).

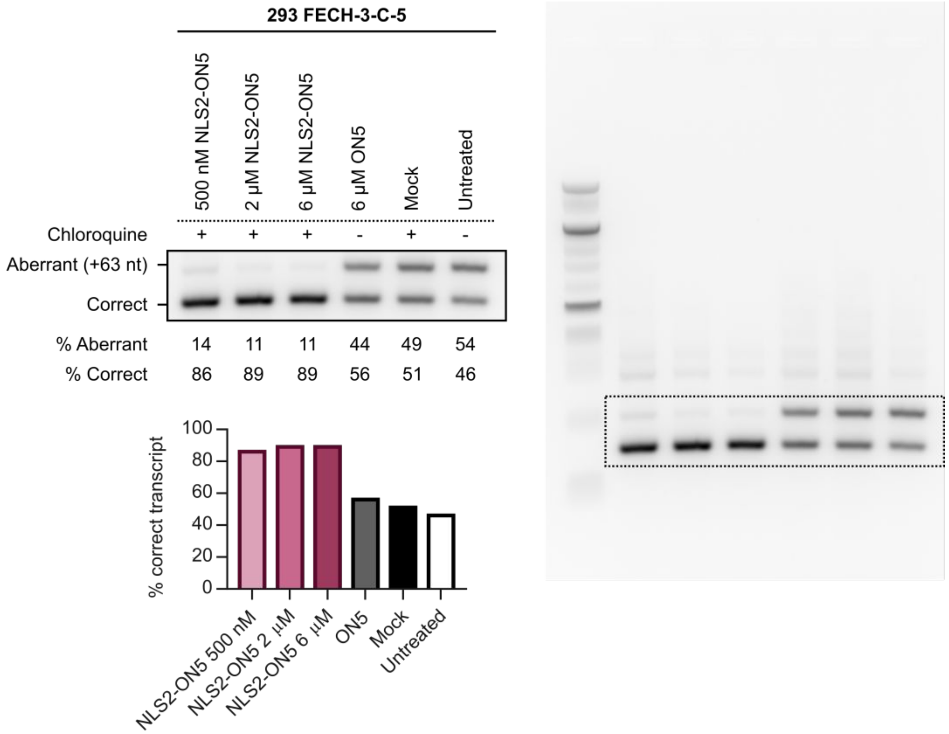

Figure S32. Splice-switching activity of the lead conjugate following free uptake and treatment with chloroquine. Semi-quantitation (*left*) and raw image (*right*) of an agarose gel separating reverse transcribed and PCR amplified FECH transcripts extracted from 293 FECH-3-C-5 cells. Cells were treated with conjugate under free uptake conditions at 500 nM, 2 µM, or 6 µM for 24 h followed by 60 µM chloroquine for 24 h. ON5 was a positive control for splice correction. Mock was treated with chloroquine alone. Percent (%) refers to percentage of total (*i.e.*, aberrant + correct) FECH transcript. The ladder was 5 µL Quick-Load Purple 100 bp DNA Ladder (New England BioLabs N0551S).

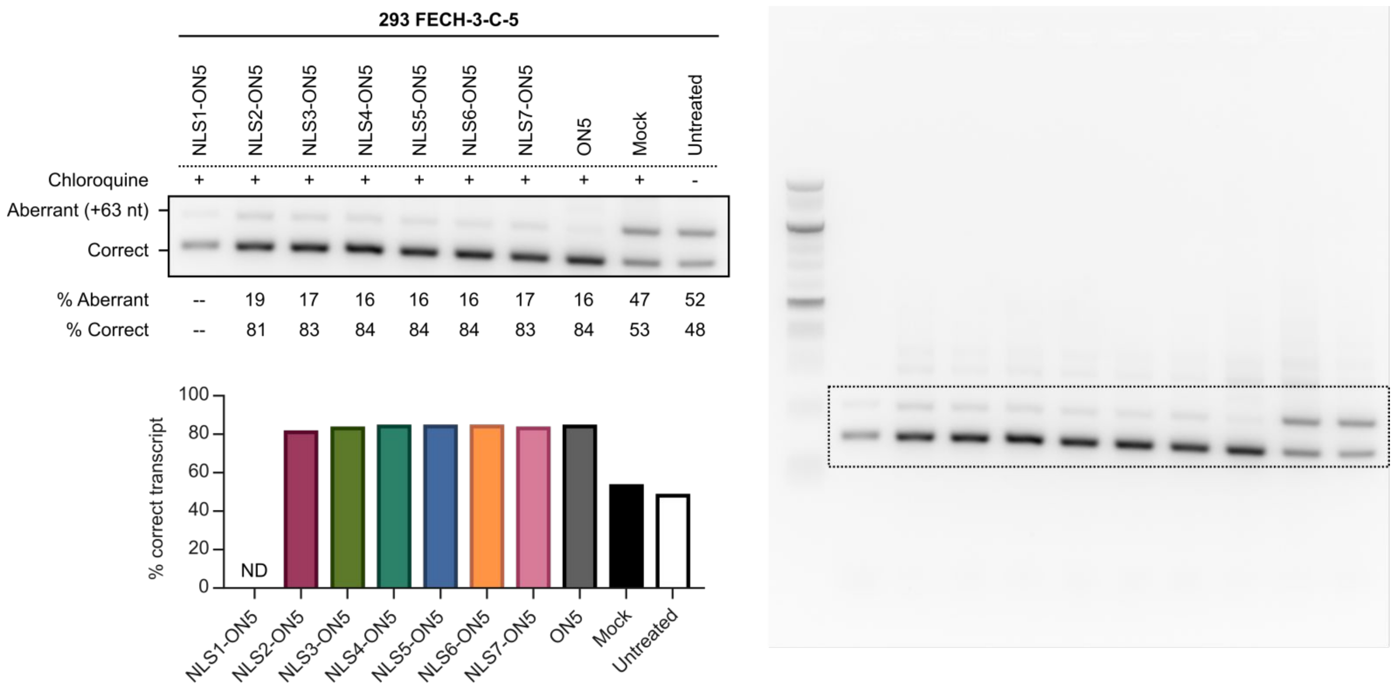

Figure S33. Splice-switching activities of all conjugates and parent SSO following free uptake and treatment with chloroquine, replicate 1. Semi-quantitation (left) and raw image (right) of an agarose gel separating reverse transcribed and PCR amplified FECH transcripts extracted from 293 FECH-3-C-5 cells. Cells were treated with conjugate or oligonucleotide under free uptake conditions at 500 nM for 24 h followed by 60 µM chloroquine for 24 h. Mock was treated with chloroquine alone. Percent (%) refers to percentage of total (*i.e.*, aberrant + correct) FECH transcript. ND, not determined. The semi-quantitation of bands for NLS1-ON5 was not possible owing to a limited amount of RNA recovered from the cells treated with this compound. The ladder was 5 µL Quick-Load Purple 100 bp DNA Ladder (New England BioLabs N0551S).

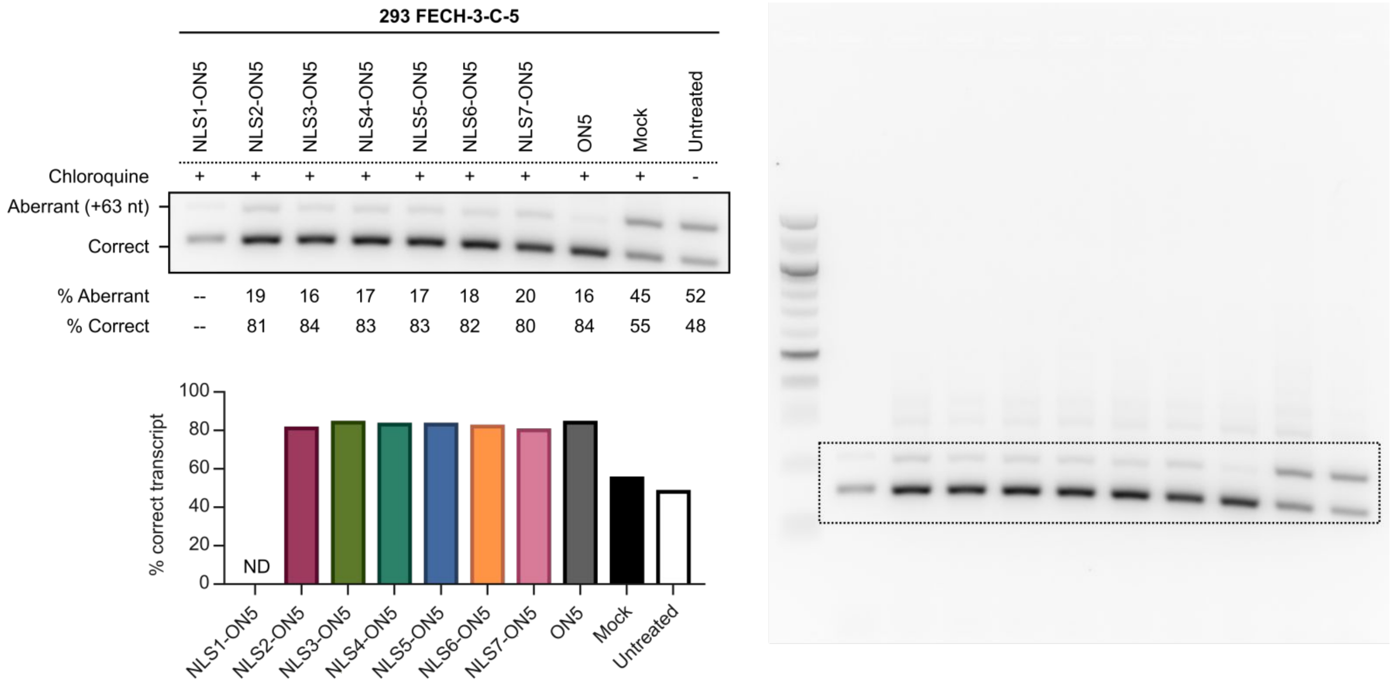

Figure S34. Splice-switching activities of all conjugates and parent SSO following free uptake and treatment with chloroquine, replicate 2. Semi-quantitation (left) and raw image (right) of an agarose gel separating reverse transcribed and PCR amplified FECH transcripts extracted from 293 FECH-3-C-5 cells. Cells were treated with conjugate or oligonucleotide under free uptake conditions at 500 nM for 24 h followed by 60 µM chloroquine for 24 h. Mock was treated with chloroquine alone. Percent (%) refers to percentage of total (*i.e.*, aberrant + correct) FECH transcript. ND, not determined. The semi-quantitation of bands for NLS1-ON5 was not possible owing to a limited amount of RNA recovered from the cells treated with this compound. The ladder was 5 µL Quick-Load Purple 100 bp DNA Ladder (New England BioLabs N0551S).

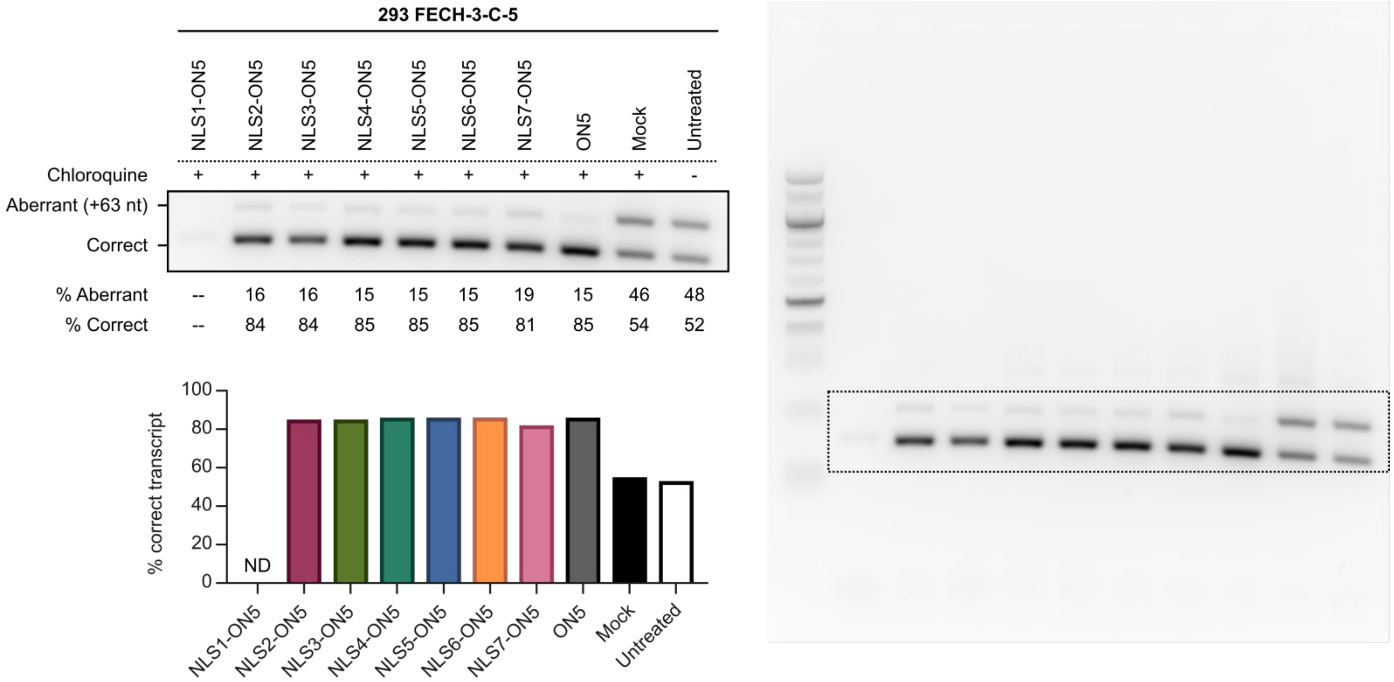

Figure S35. Splice-switching activities of all conjugates and parent SSO following free uptake and treatment with chloroquine, replicate 3. Semi-quantitation (left) and raw image (right) of an agarose gel separating reverse transcribed and PCR amplified FECH transcripts extracted from 293 FECH-3-C-5 cells. Cells were treated with conjugate or oligonucleotide under free uptake conditions at 500 nM for 24 h followed by 60 µM chloroquine for 24 h. Mock was treated with chloroquine alone. Percent (%) refers to percentage of total (*i.e.*, aberrant + correct) FECH transcript. ND, not determined. The semi-quantitation of bands for NLS1-ON5 was not possible owing to a limited amount of RNA recovered from the cells treated with this compound. The ladder was 5 µL Quick-Load Purple 100 bp DNA Ladder (New England BioLabs N0551S).
